# Supplementary figures and images for: miR-34a-5p inhibits the malignant progression of KSHV-infected SH-SY5Y cells by targeting c-fos
Source: PeerJ. 2022 Apr 15;10:e13233. doi: 10.7717/peerj.13233 (PMC9014853; doi:10.7717/peerj.13233)

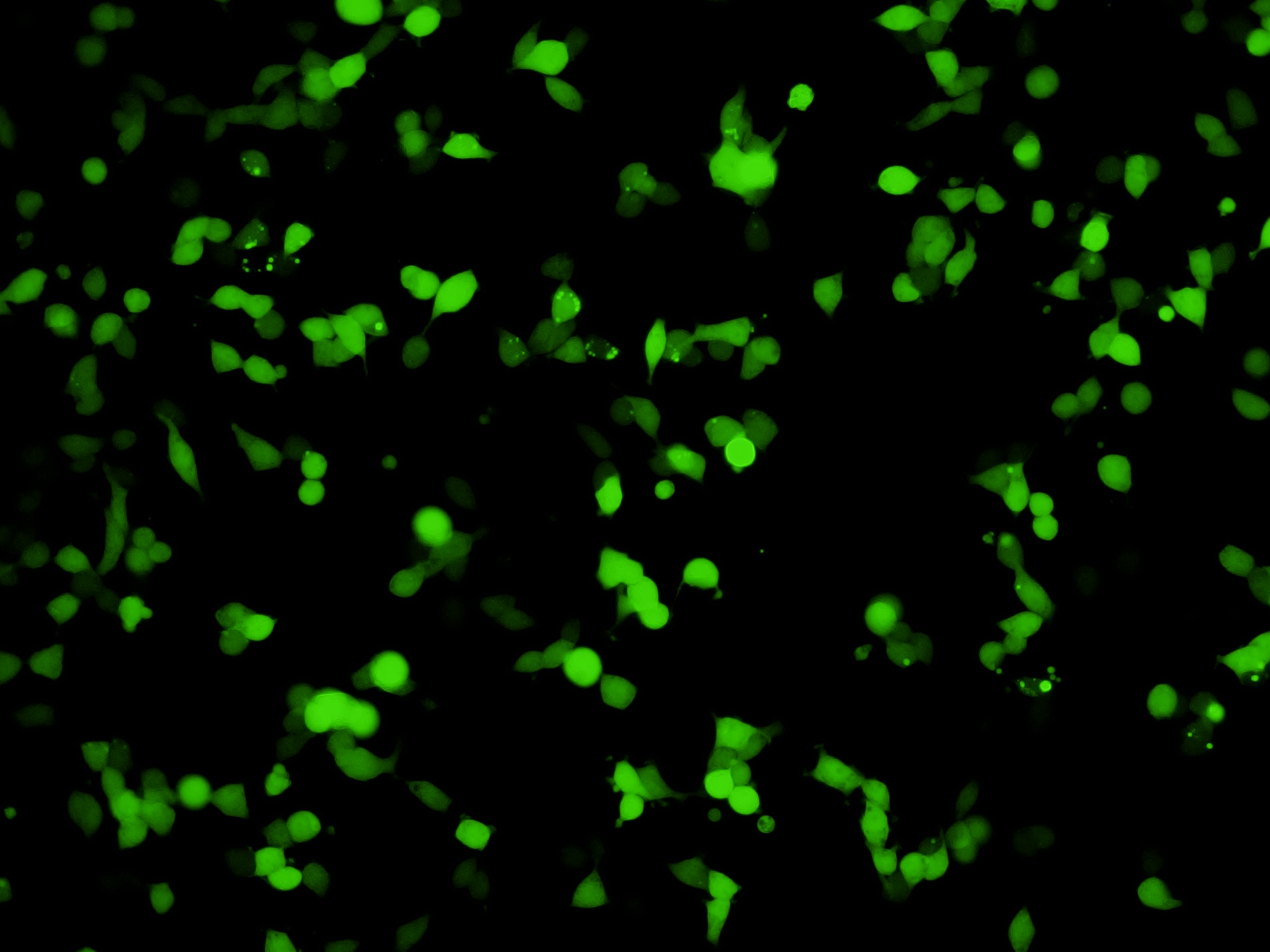

Supplement: Supplemental Information 1 [file peerj-10-13233-s001.zip › figure1/figure 1A/GFP(200X).tif]

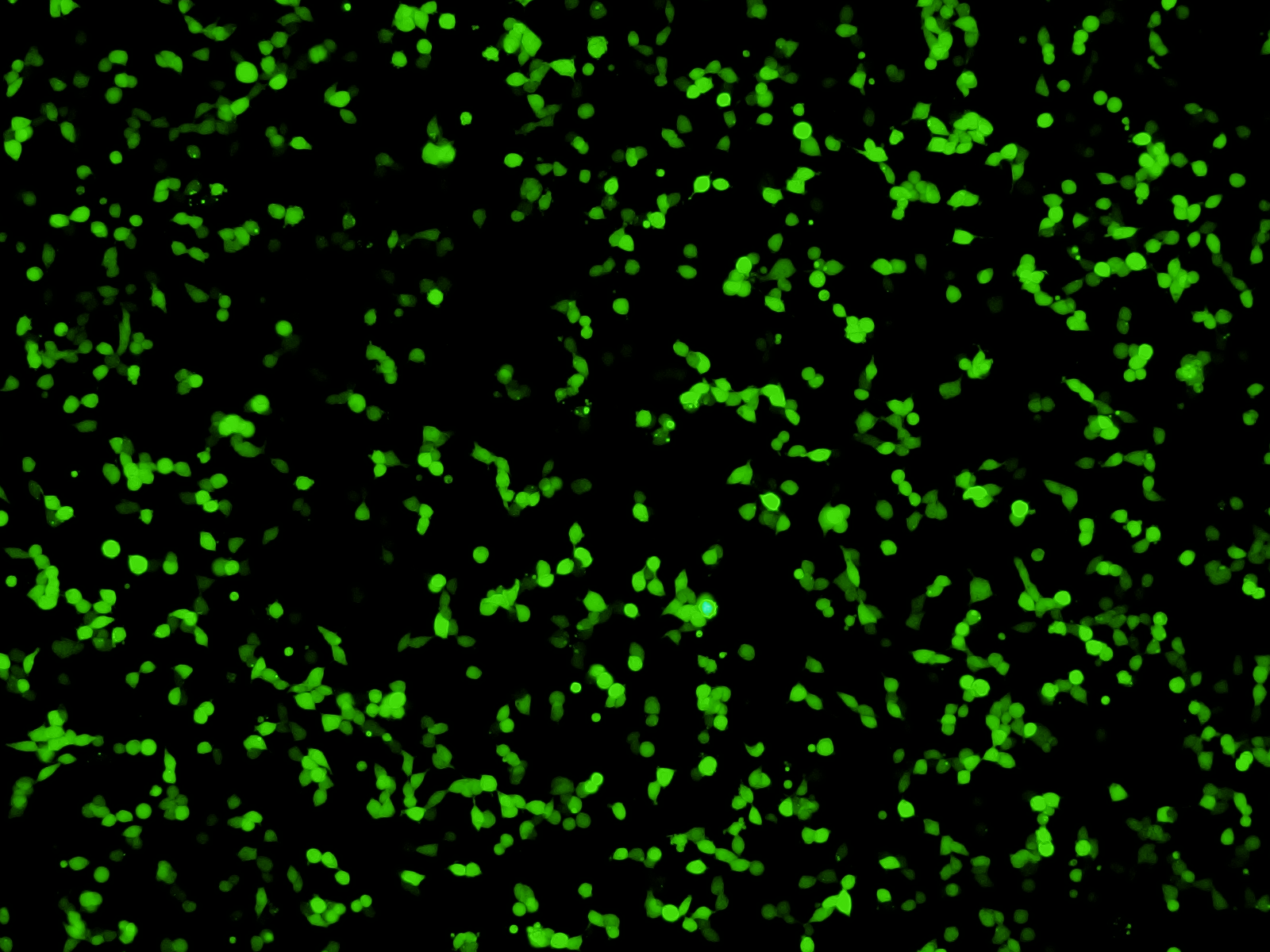

Supplement: Supplemental Information 1 [file peerj-10-13233-s001.zip › figure1/figure 1A/GFP(40X).tif]

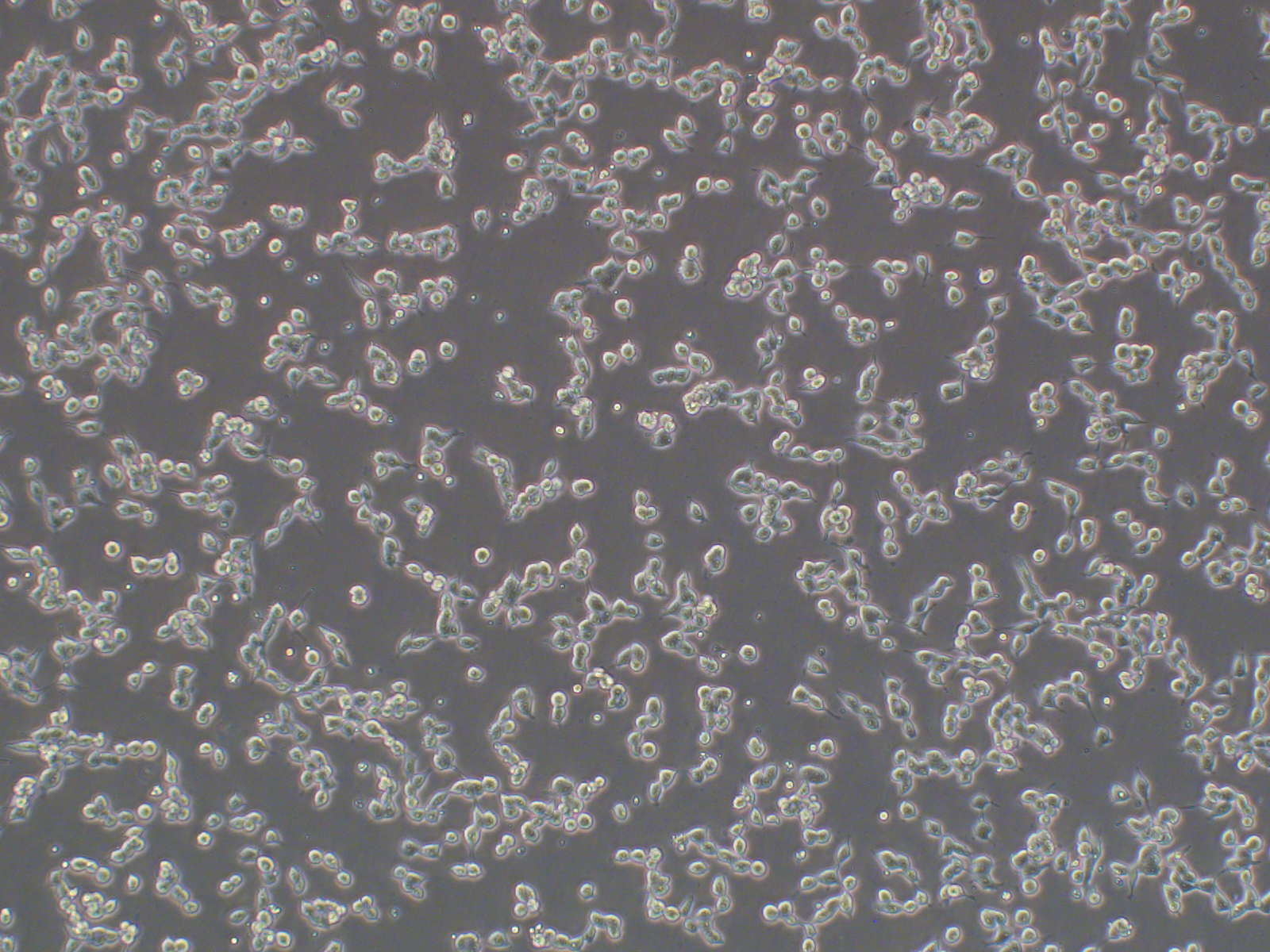

Supplement: Supplemental Information 1 [file peerj-10-13233-s001.zip › figure1/figure 1A/light(40X).tif]

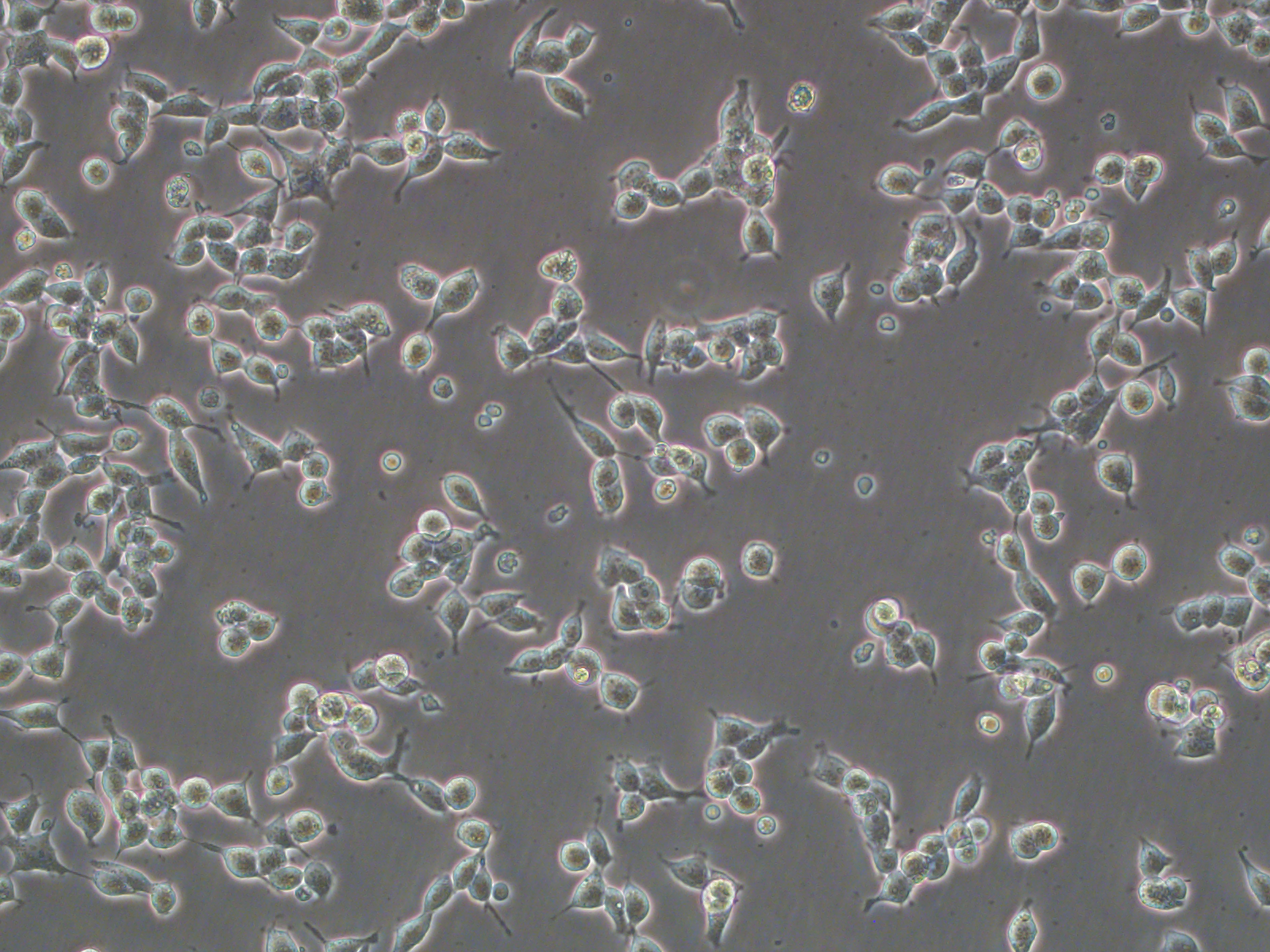

Supplement: Supplemental Information 1 [file peerj-10-13233-s001.zip › figure1/figure 1A/light(200X).tif]

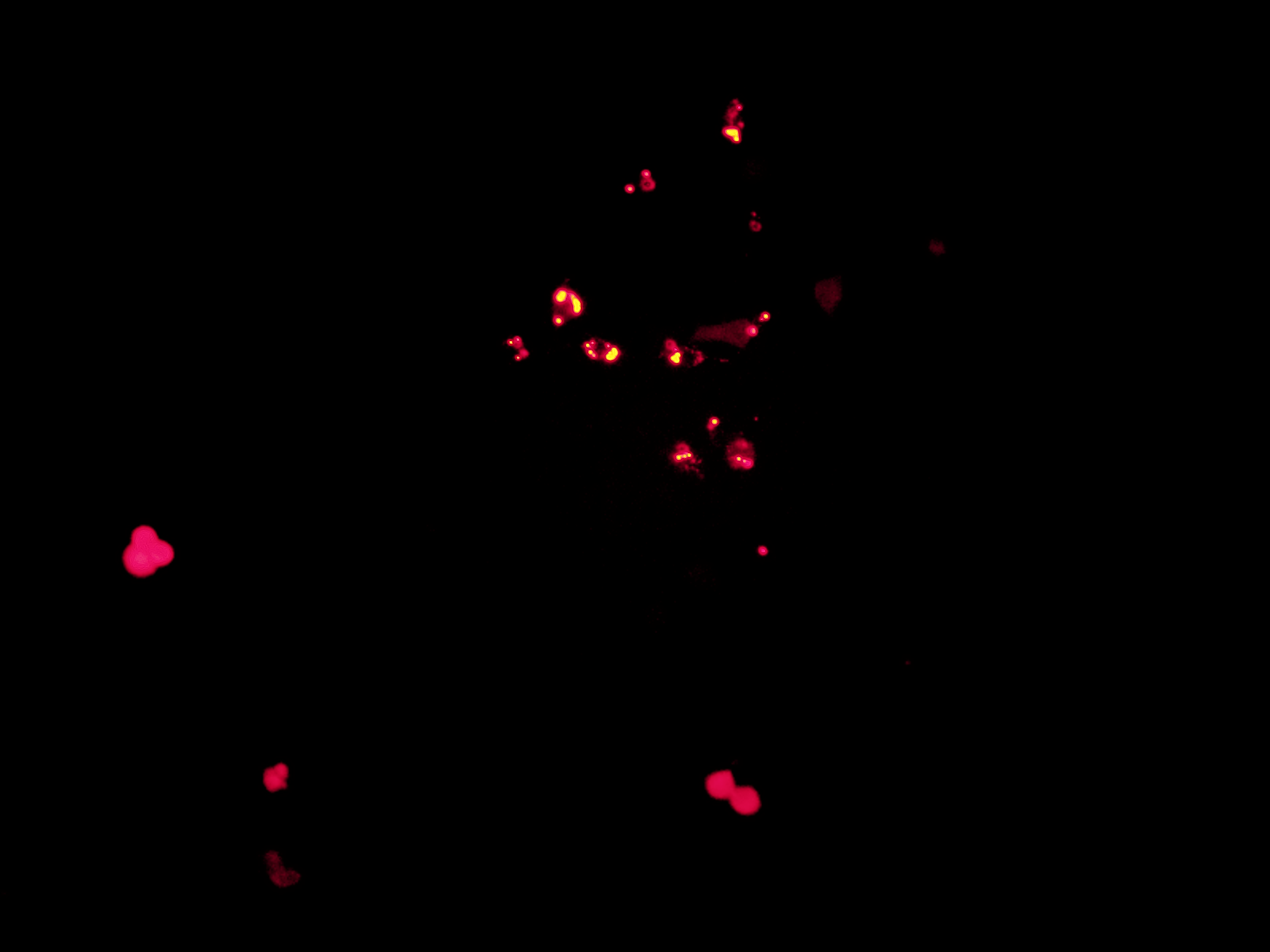

Supplement: Supplemental Information 1 [file peerj-10-13233-s001.zip › figure1/figure 1A/RFP(200X).tif]

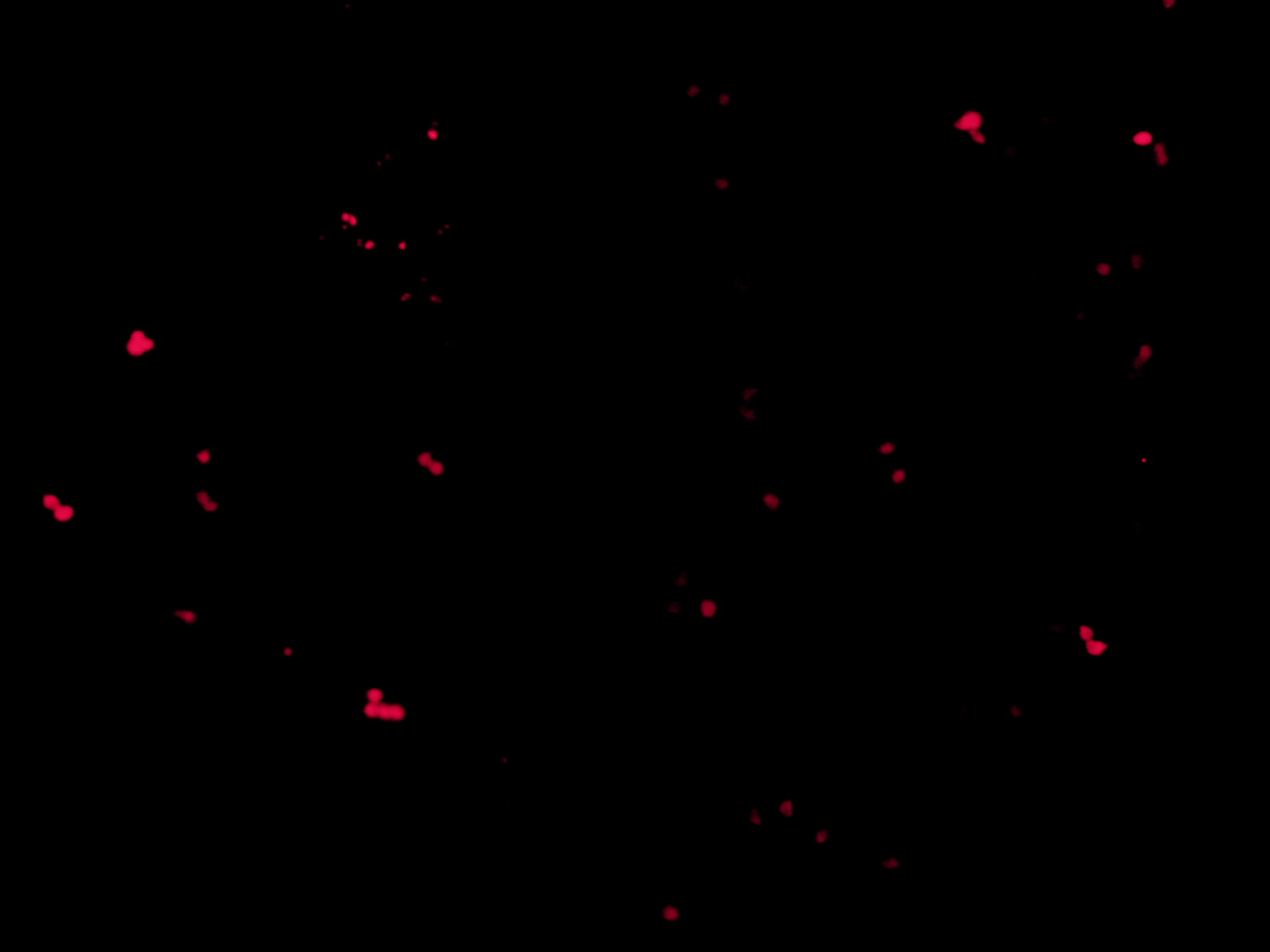

Supplement: Supplemental Information 1 [file peerj-10-13233-s001.zip › figure1/figure 1A/RFP(40X).tif]

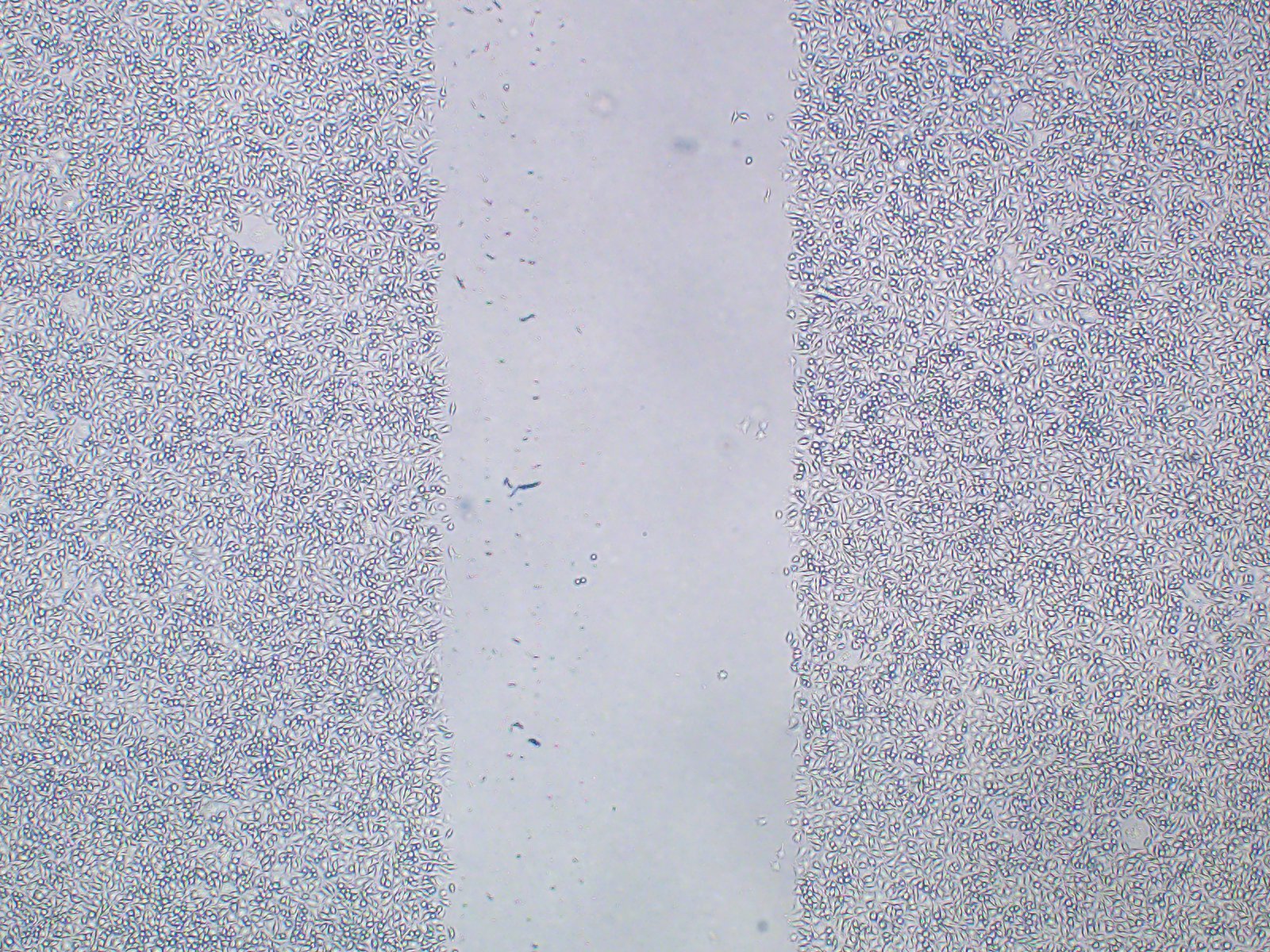

Supplement: Supplemental Information 2 [file peerj-10-13233-s002.zip › figure S1/supplement figure1C/inhibitor-NC 0h.jpg]

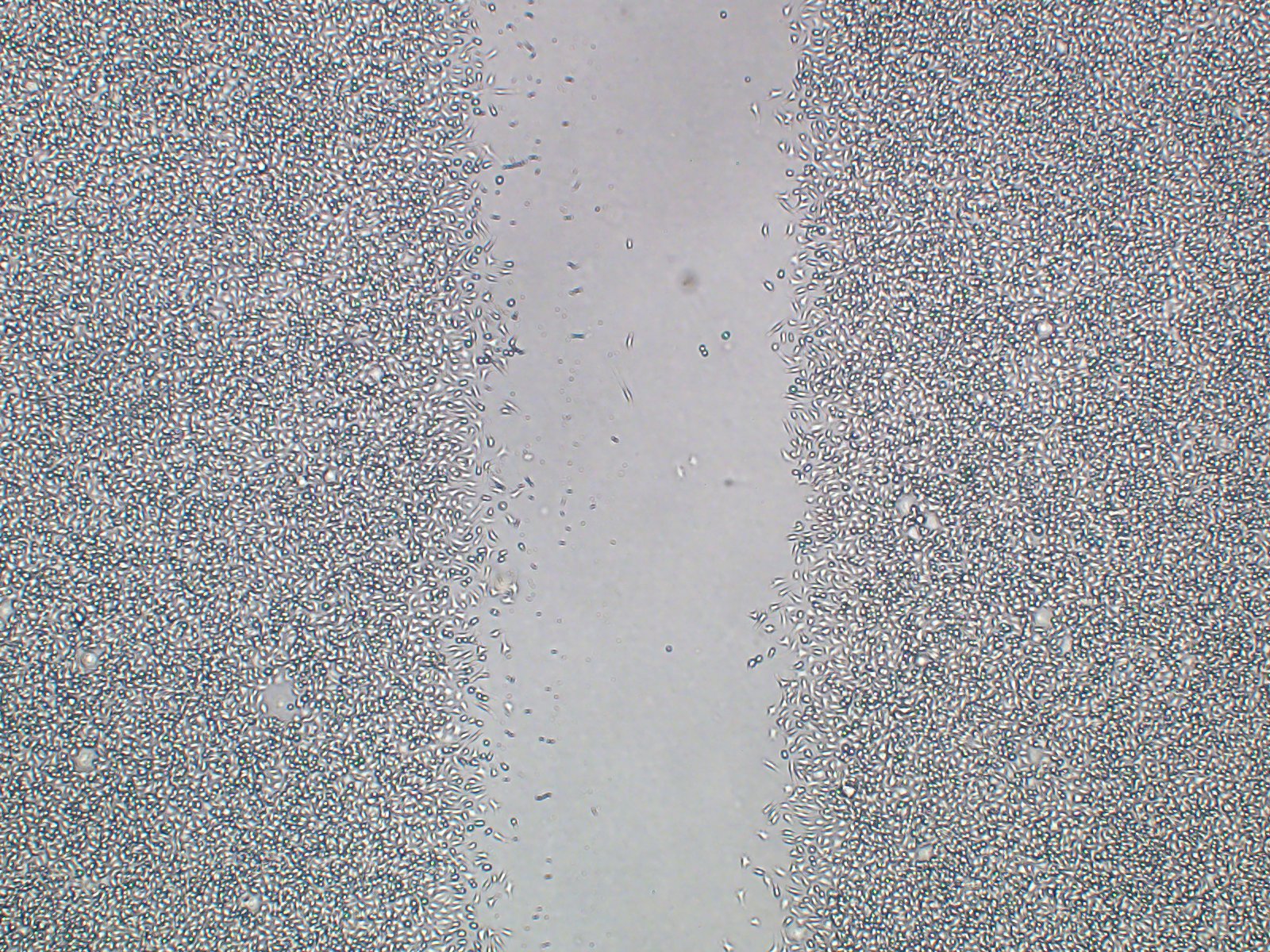

Supplement: Supplemental Information 2 [file peerj-10-13233-s002.zip › figure S1/supplement figure1C/inhibitor-NC 12h.jpg]

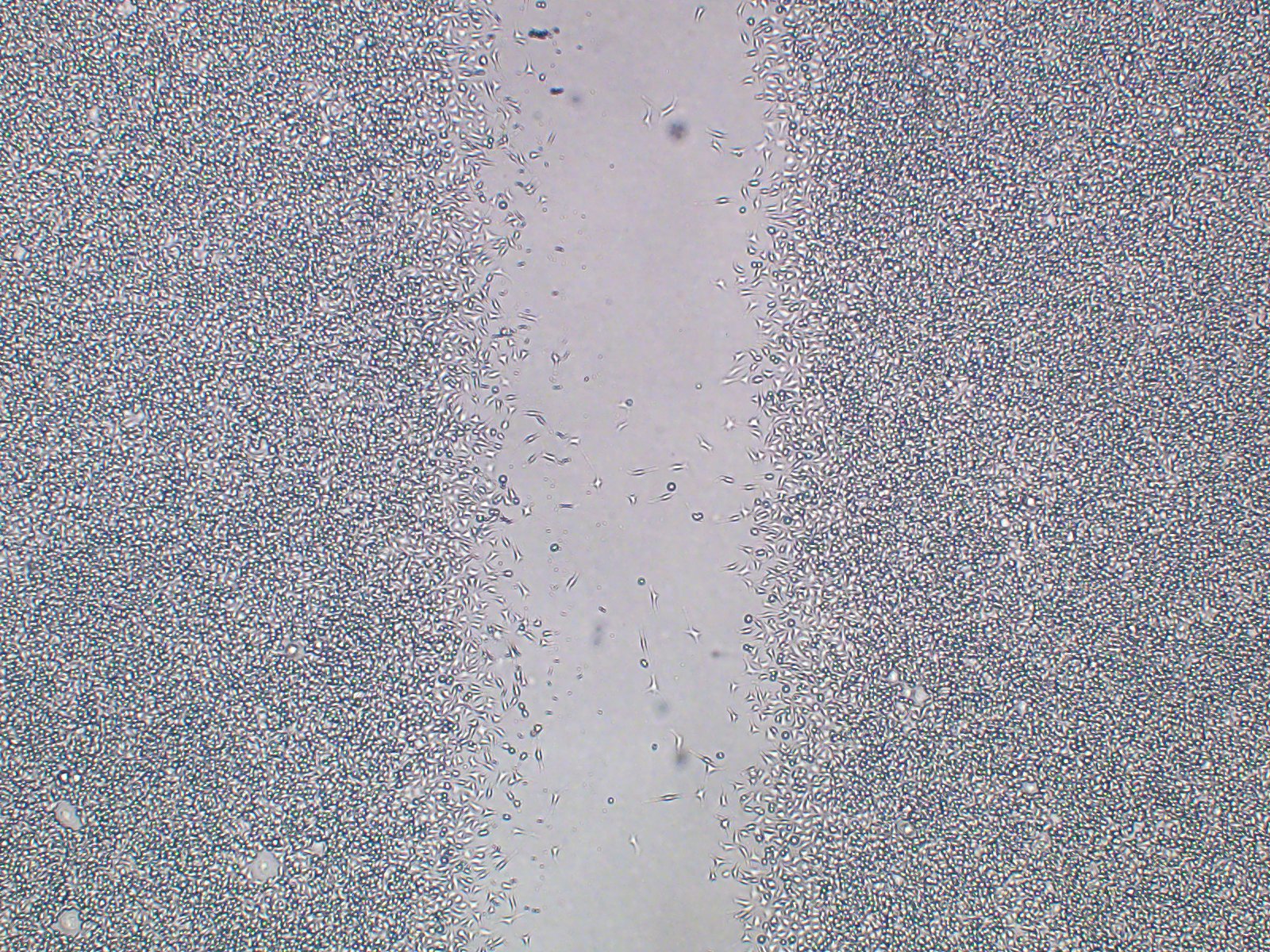

Supplement: Supplemental Information 2 [file peerj-10-13233-s002.zip › figure S1/supplement figure1C/inhibitor-NC 24h.jpg]

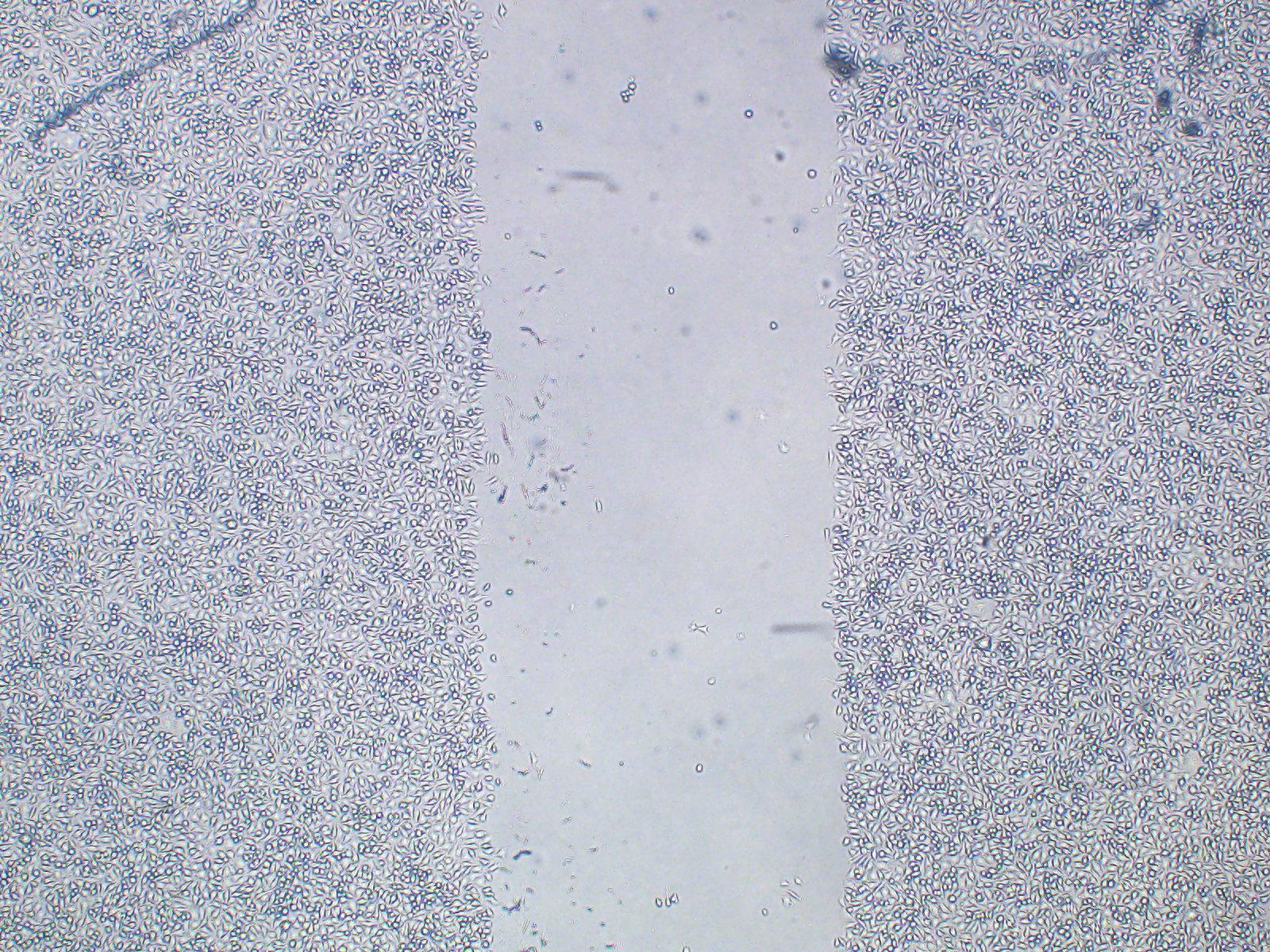

Supplement: Supplemental Information 2 [file peerj-10-13233-s002.zip › figure S1/supplement figure1C/miR-34a-5p inhibitor 0h.jpg]

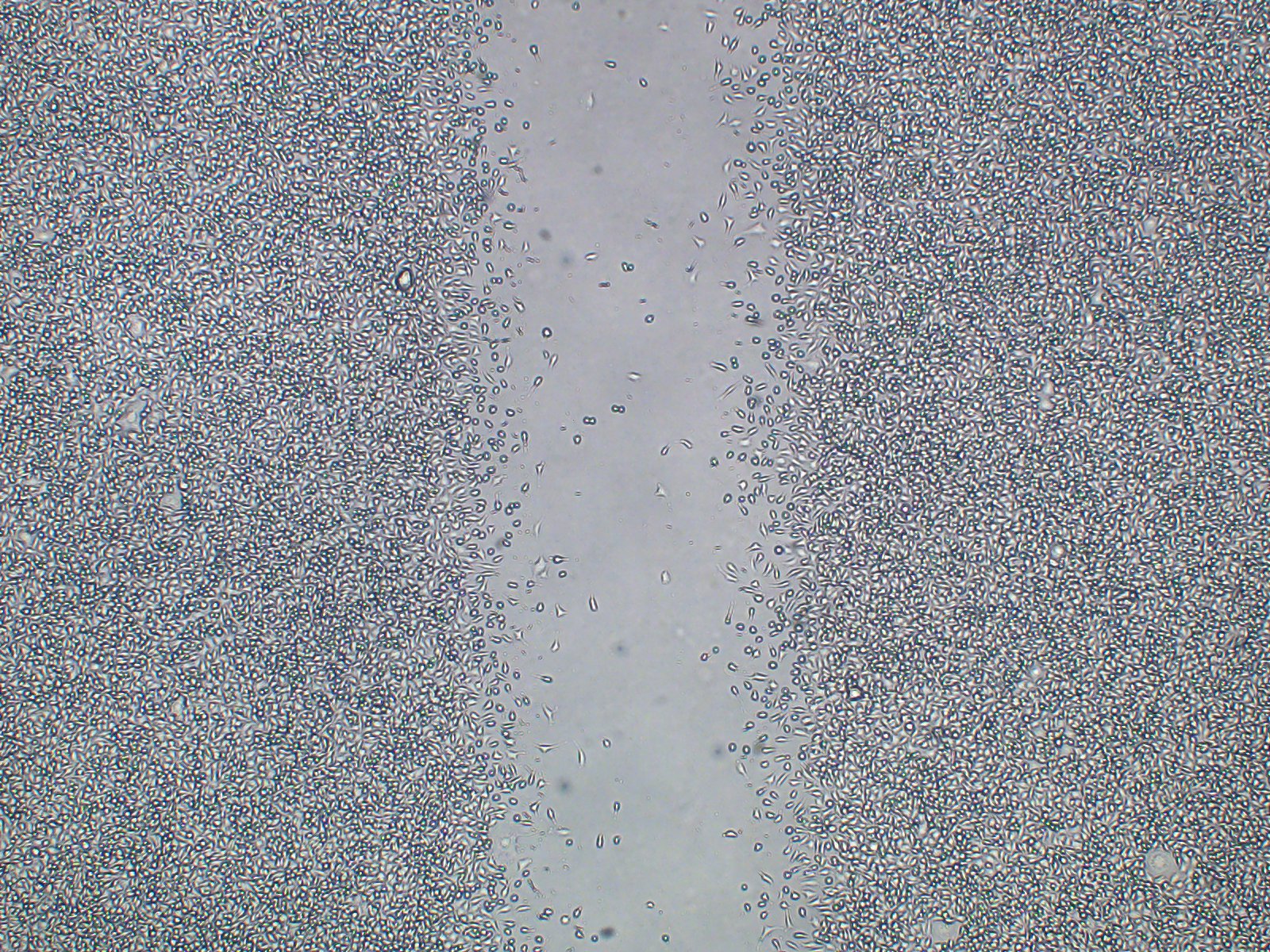

Supplement: Supplemental Information 2 [file peerj-10-13233-s002.zip › figure S1/supplement figure1C/miR-34a-5p inhibitor 12h.jpg]

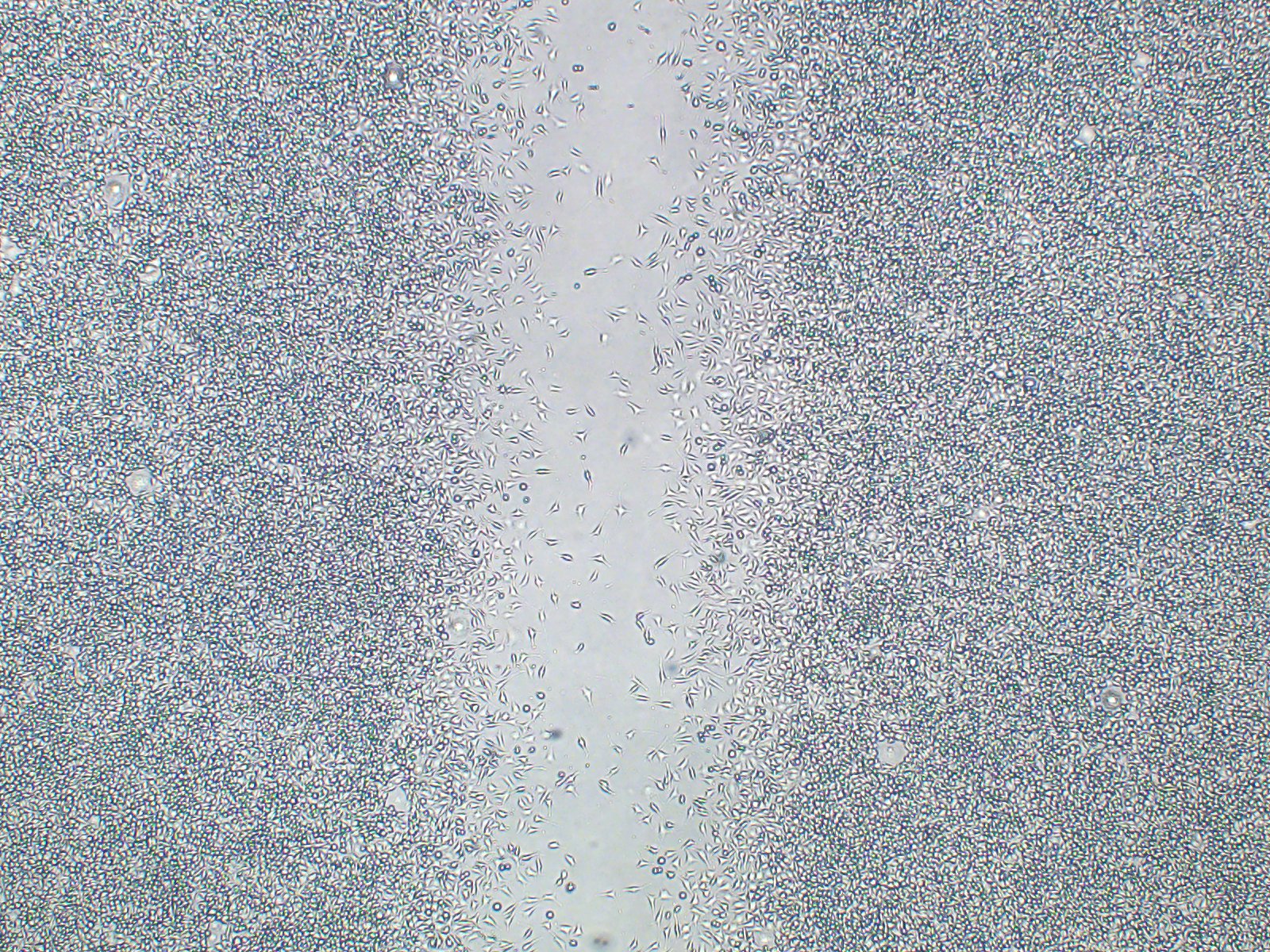

Supplement: Supplemental Information 2 [file peerj-10-13233-s002.zip › figure S1/supplement figure1C/miR-34a-5p inhibitor 24h.jpg]

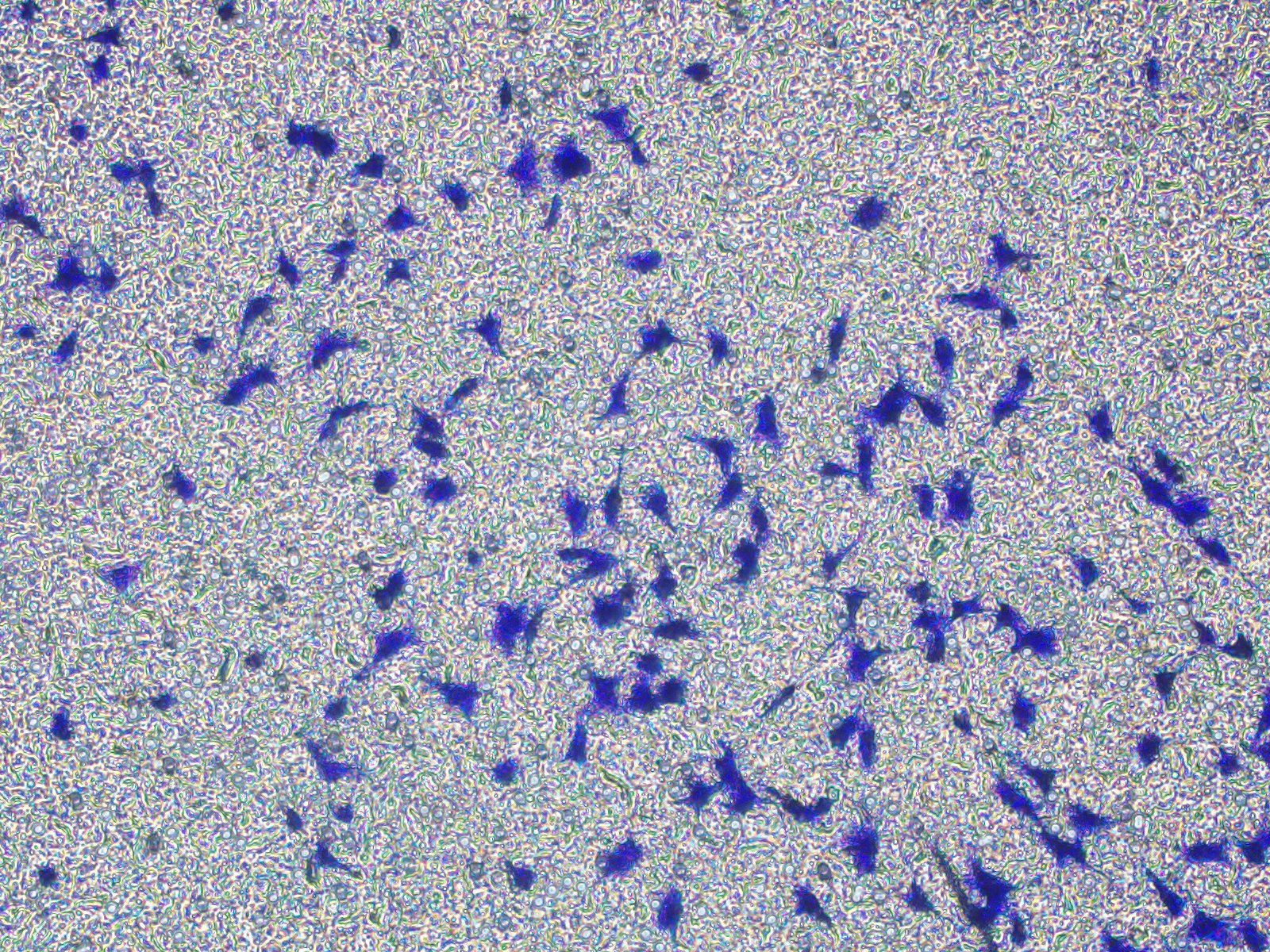

Supplement: Supplemental Information 2 [file peerj-10-13233-s002.zip › figure S1/supplement figure1E/inhibitor-NC.jpg]

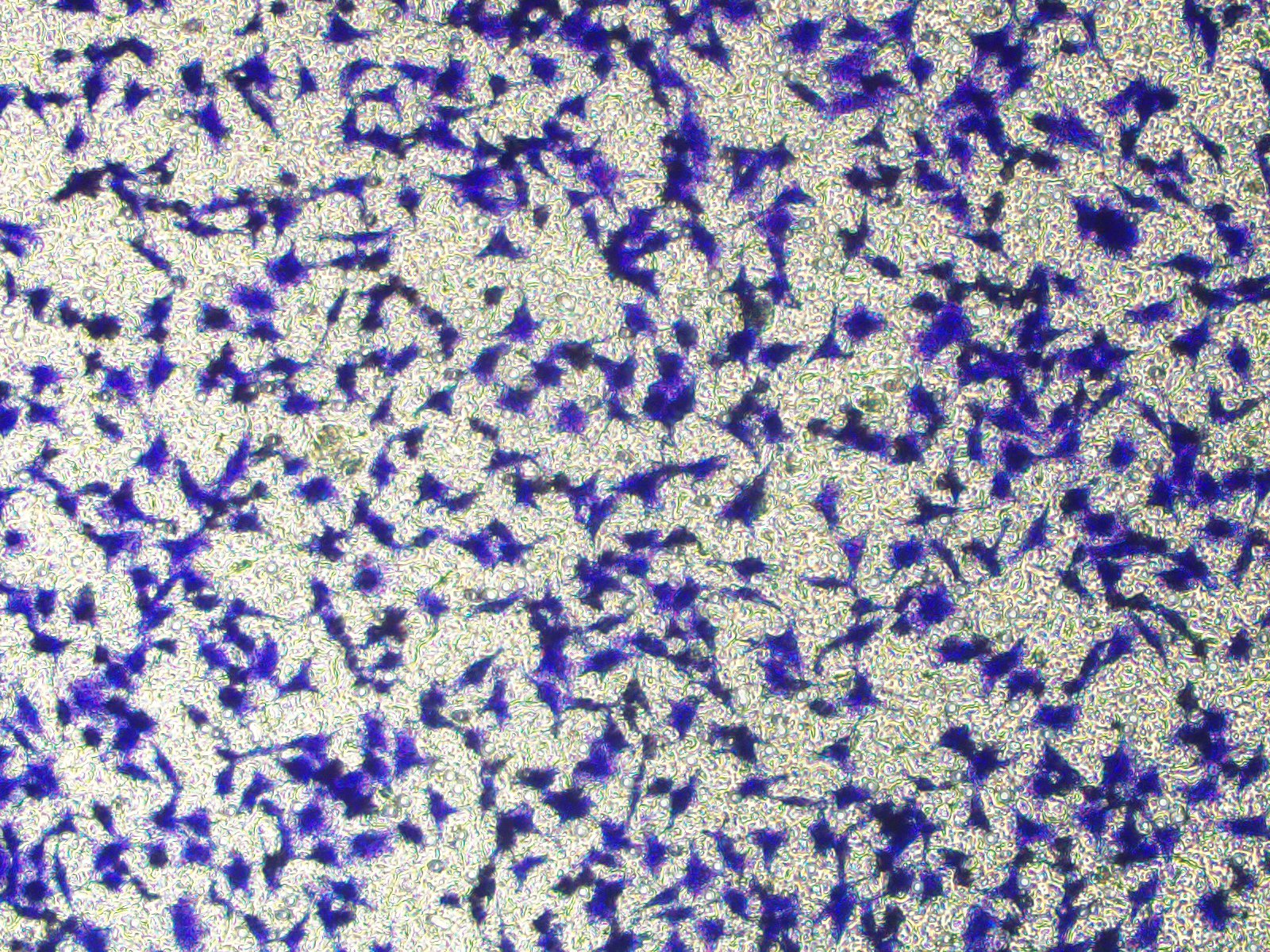

Supplement: Supplemental Information 2 [file peerj-10-13233-s002.zip › figure S1/supplement figure1E/miR-34a-5p inhibitor.jpg]

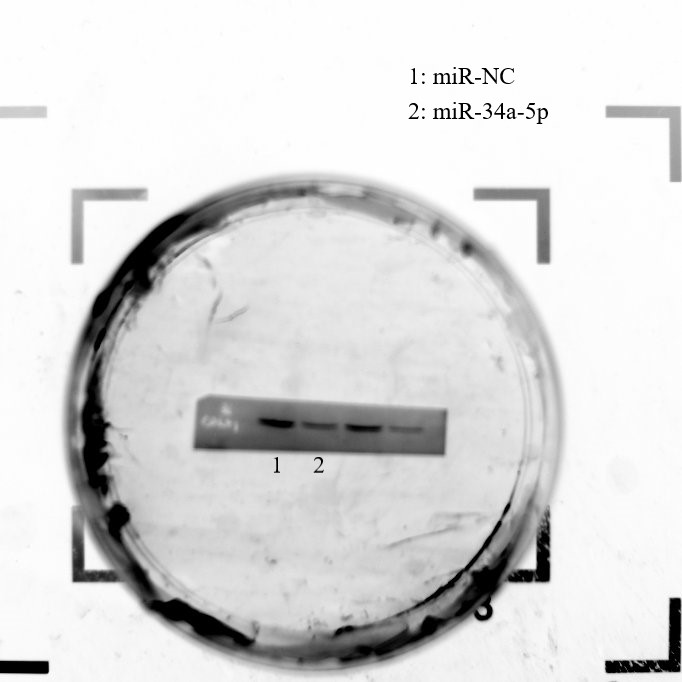

Supplement: Supplemental Information 3 [file peerj-10-13233-s003.zip › figure2/figure2 D/CDK4.jpg]

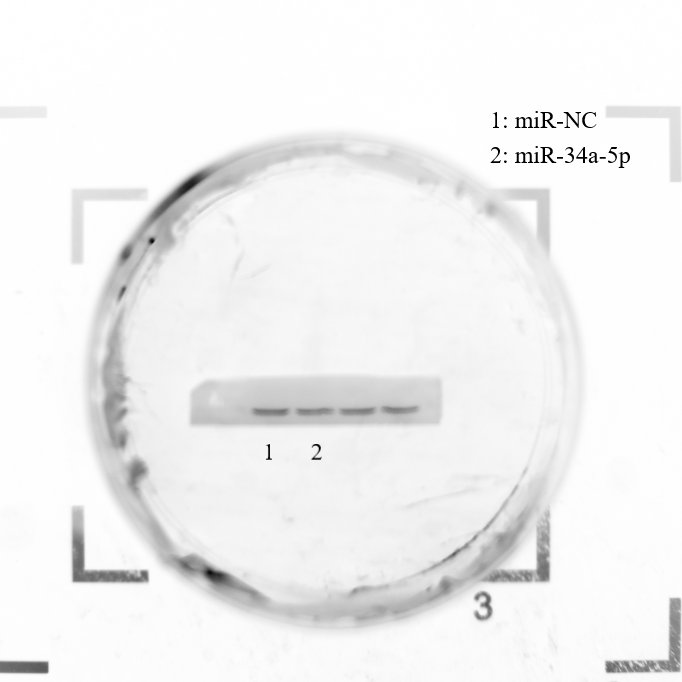

Supplement: Supplemental Information 3 [file peerj-10-13233-s003.zip › figure2/figure2 D/a┬-actin .jpg]

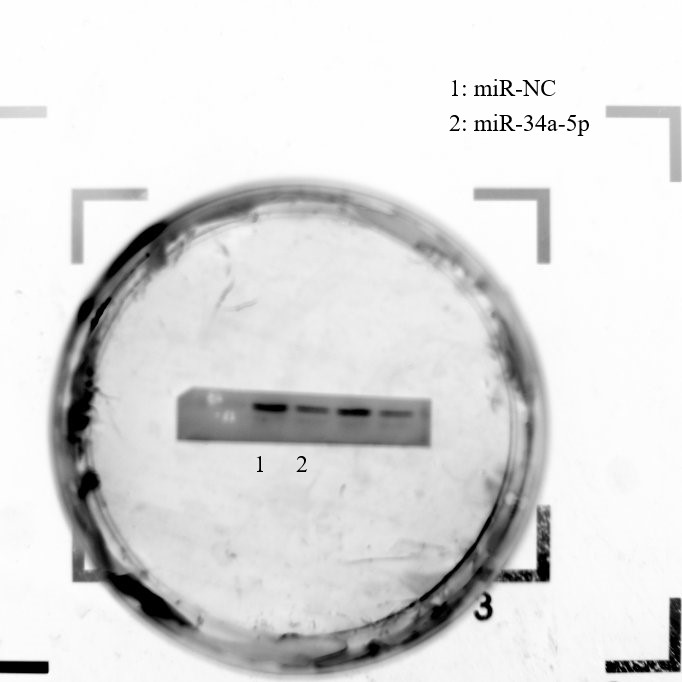

Supplement: Supplemental Information 3 [file peerj-10-13233-s003.zip › figure2/figure2 E/CDK6.jpg]

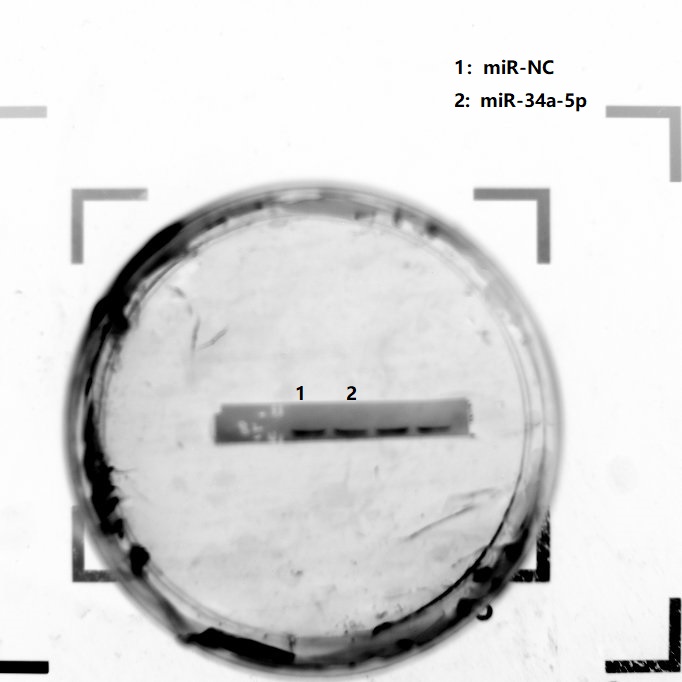

Supplement: Supplemental Information 3 [file peerj-10-13233-s003.zip › figure2/figure2 E/a┬-actin.jpg]

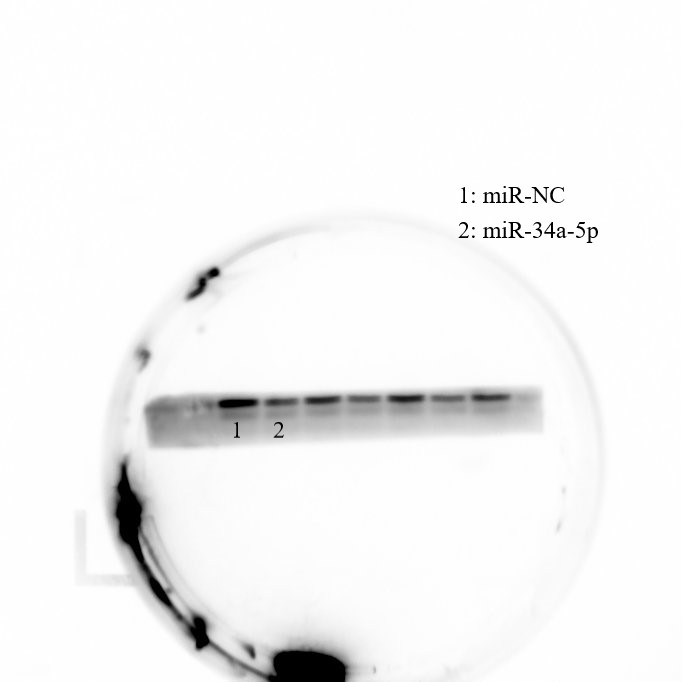

Supplement: Supplemental Information 3 [file peerj-10-13233-s003.zip › figure2/figure2 F/cyclin D1.jpg]

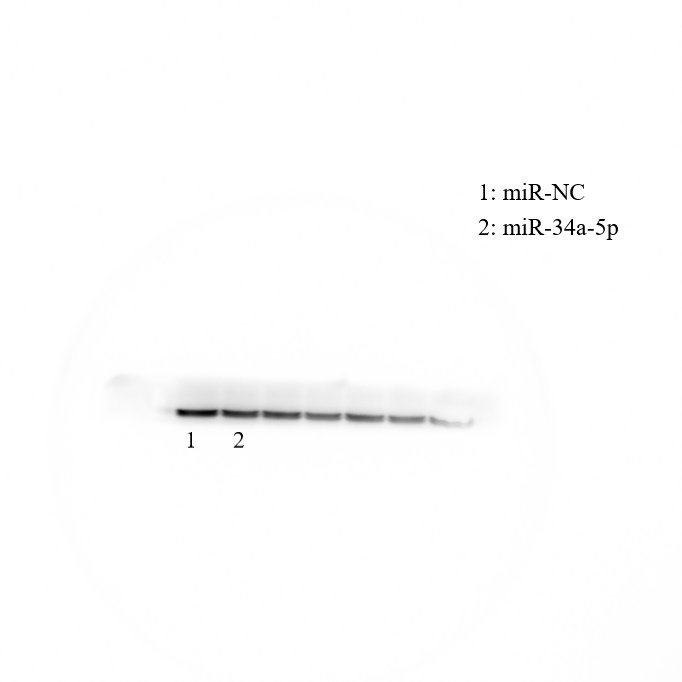

Supplement: Supplemental Information 3 [file peerj-10-13233-s003.zip › figure2/figure2 F/a┬-actin.jpg]

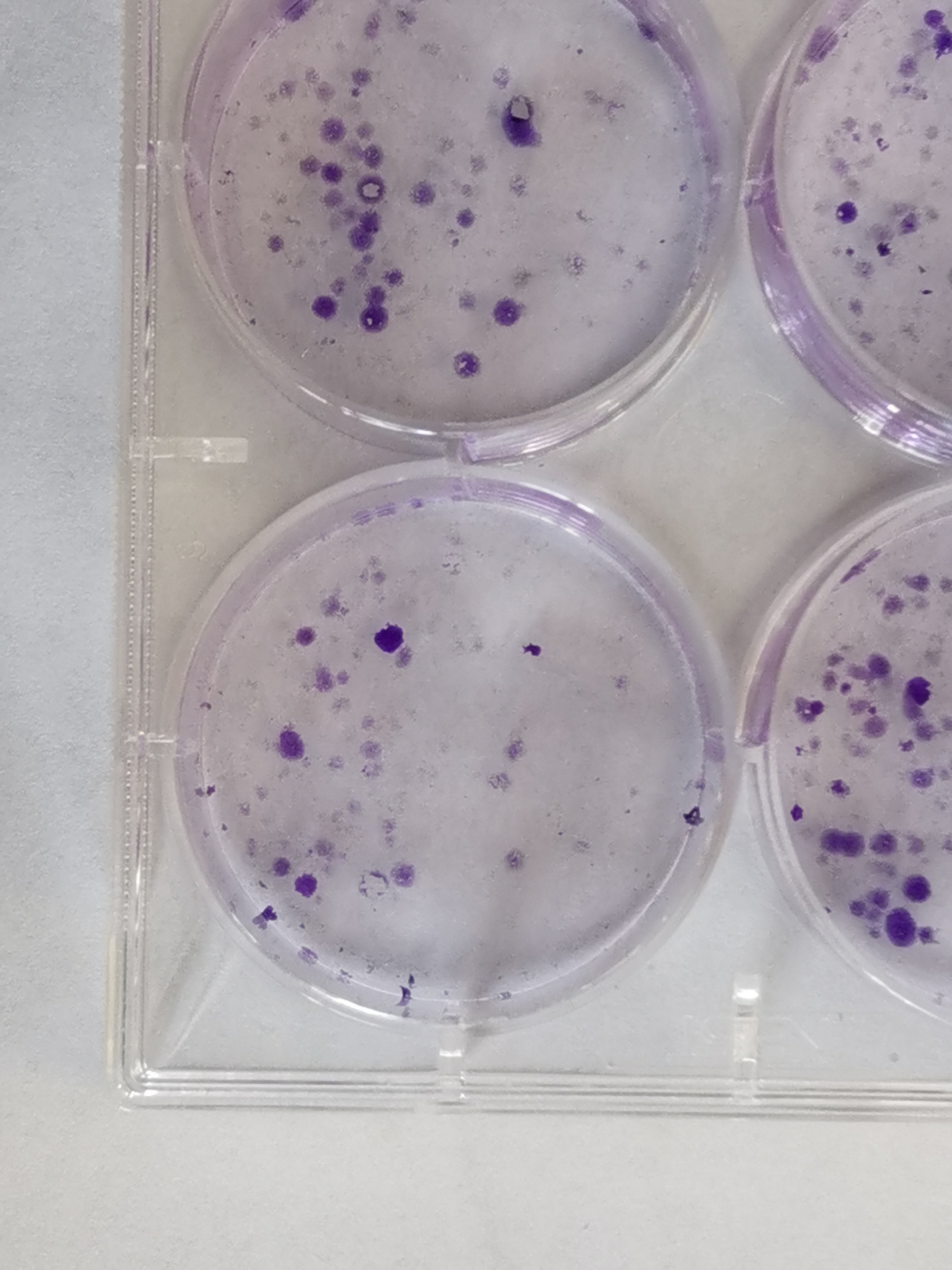

Supplement: Supplemental Information 3 [file peerj-10-13233-s003.zip › figure2/figure2 G/miR-34a-5p.jpg]

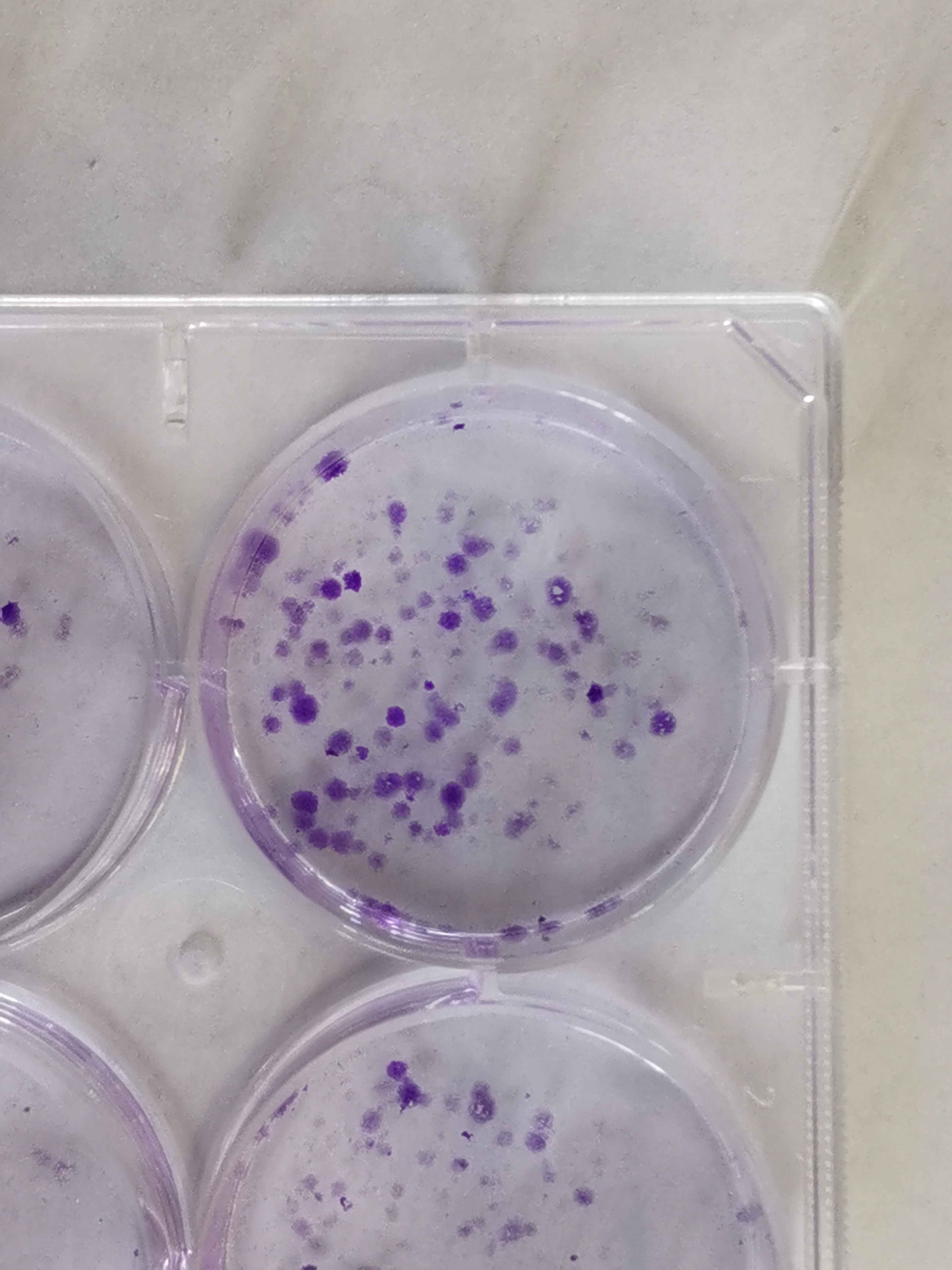

Supplement: Supplemental Information 3 [file peerj-10-13233-s003.zip › figure2/figure2 G/miR-NC.jpg]

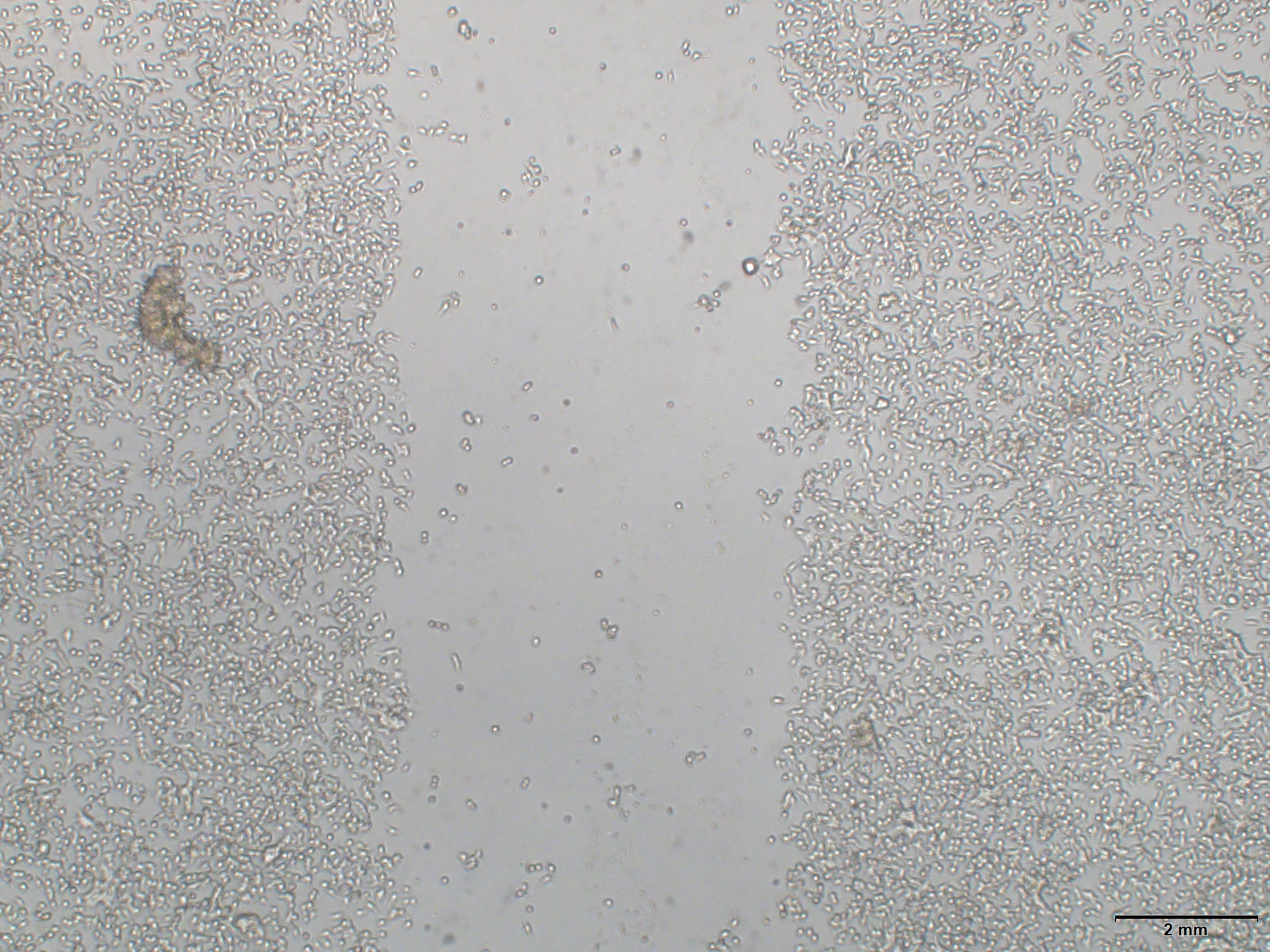

Supplement: Supplemental Information 4 [file peerj-10-13233-s004.zip › figure3/figure3 A/miR-34a-5p(0 h).jpg]

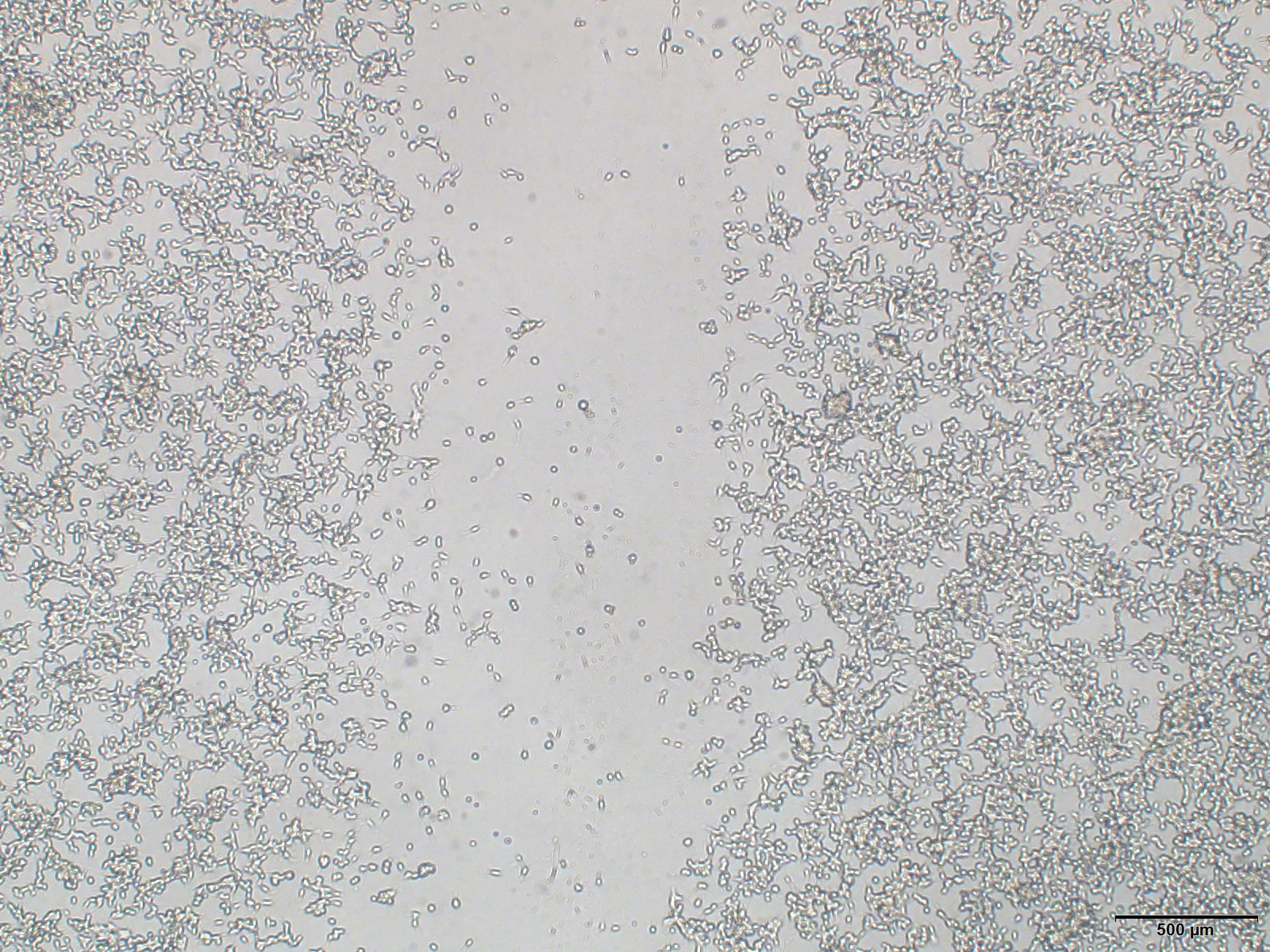

Supplement: Supplemental Information 4 [file peerj-10-13233-s004.zip › figure3/figure3 A/miR-34a-5p(12 h).jpg]

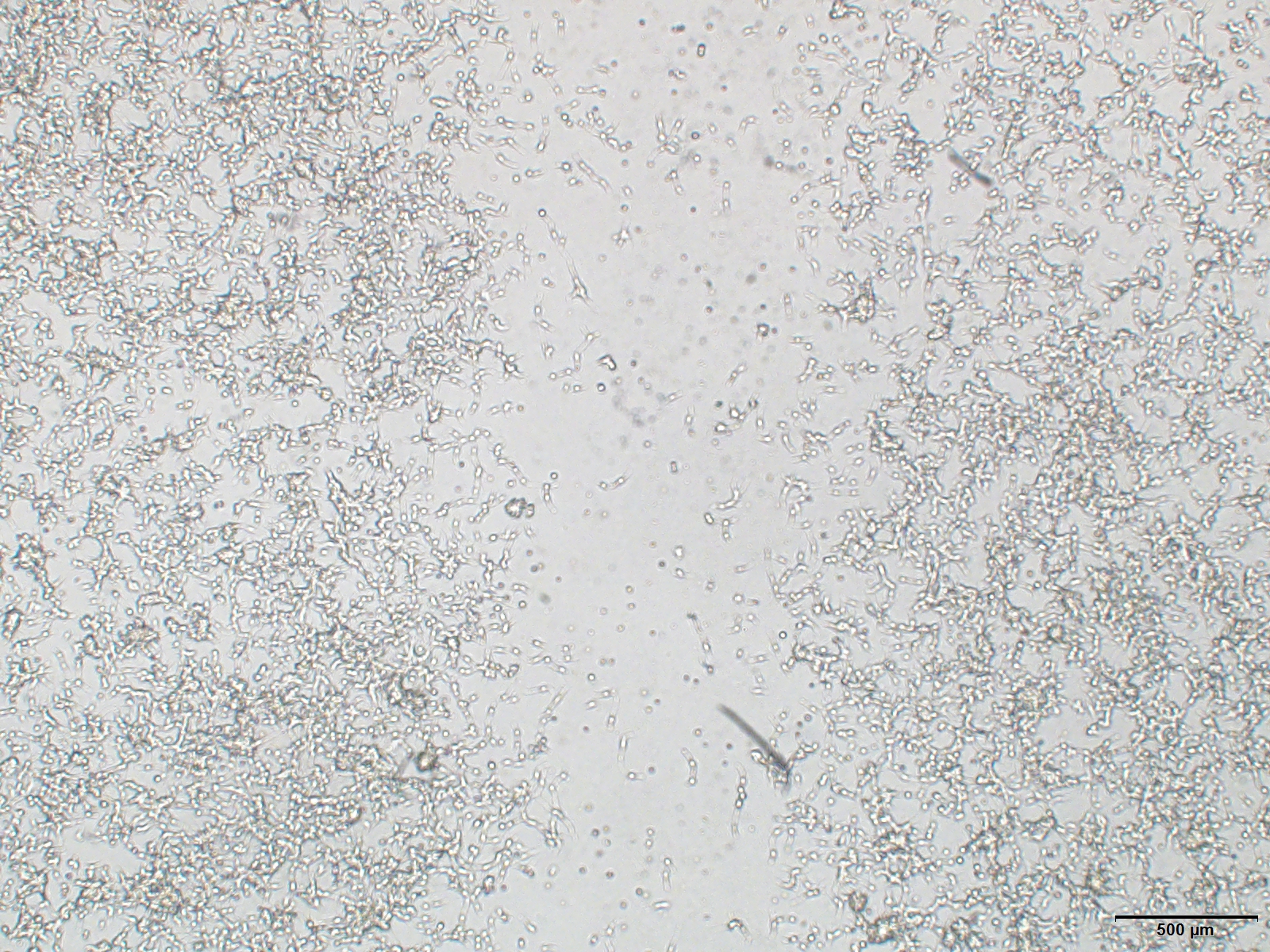

Supplement: Supplemental Information 4 [file peerj-10-13233-s004.zip › figure3/figure3 A/miR-34a-5p(36 h).jpg]

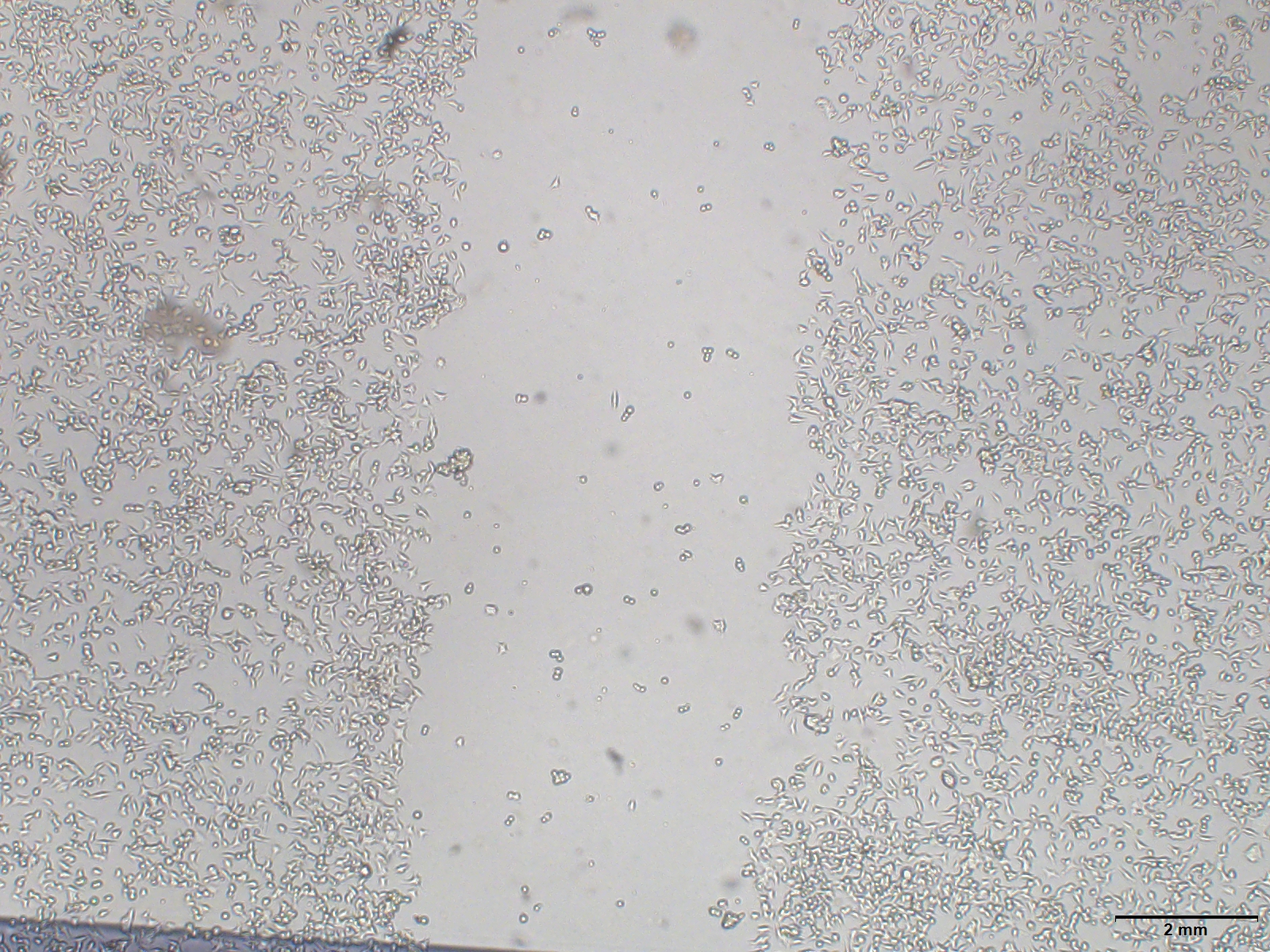

Supplement: Supplemental Information 4 [file peerj-10-13233-s004.zip › figure3/figure3 A/miR-NC(0 h).jpg]

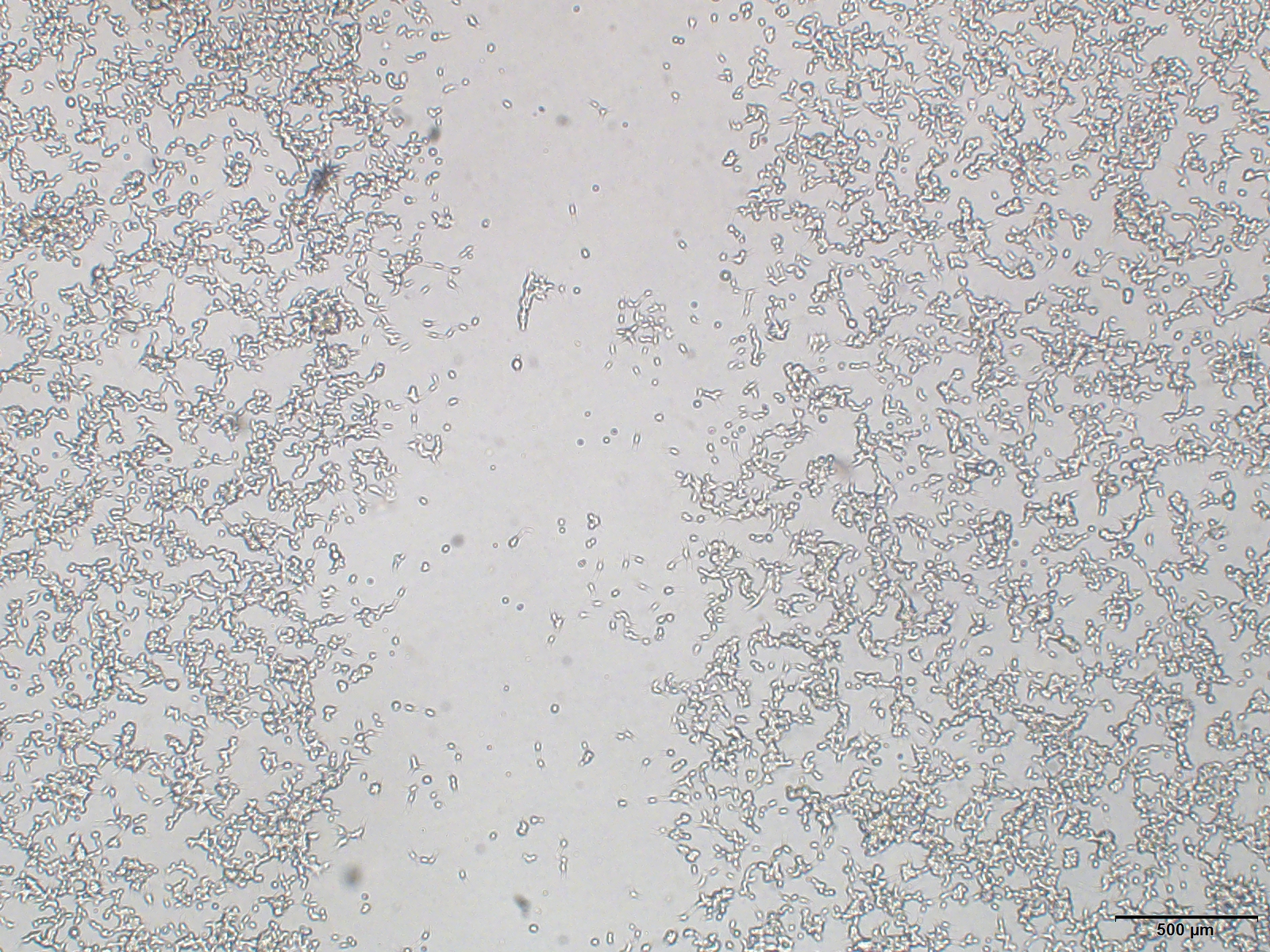

Supplement: Supplemental Information 4 [file peerj-10-13233-s004.zip › figure3/figure3 A/miR-NC(12 h).jpg]

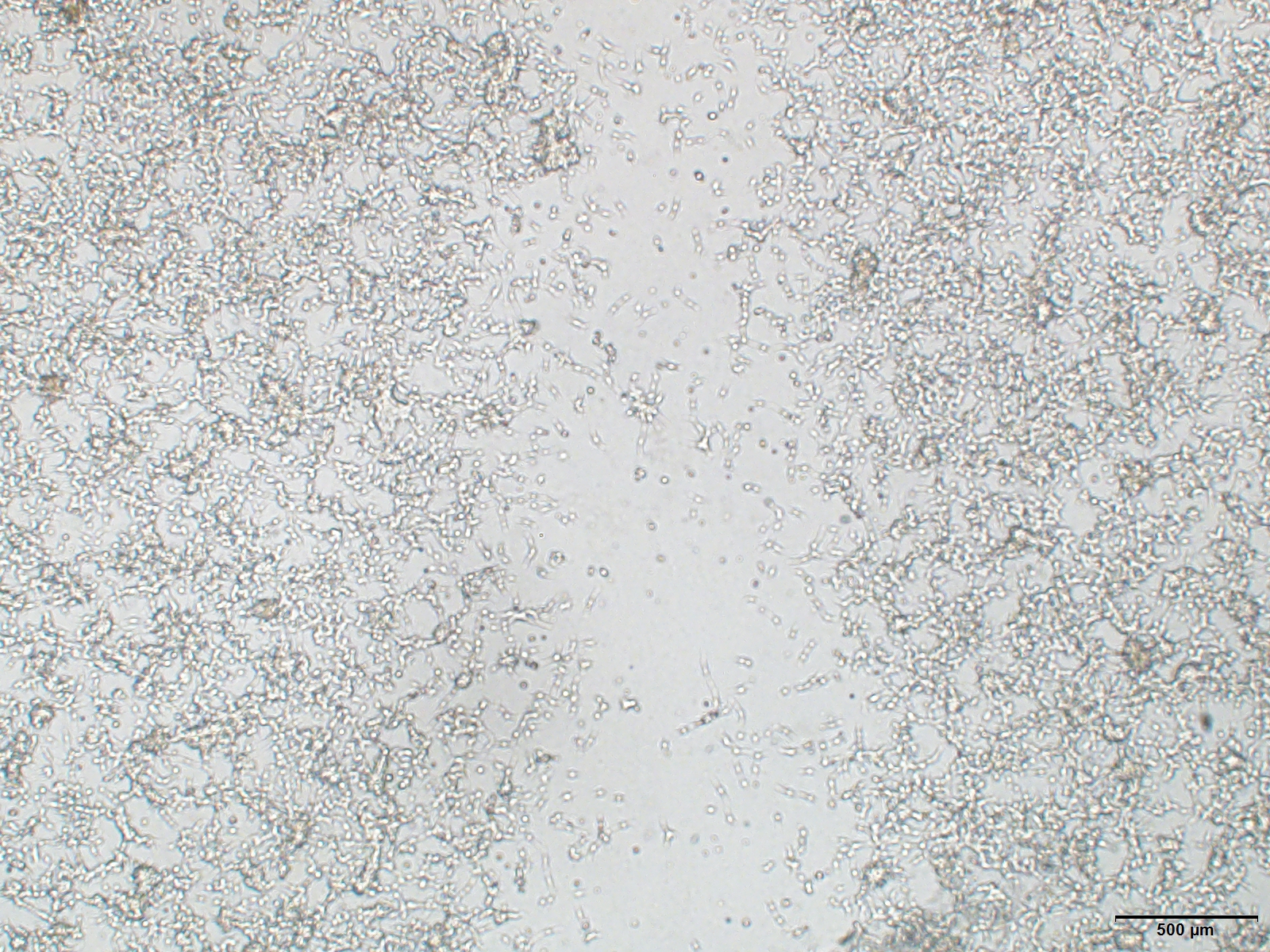

Supplement: Supplemental Information 4 [file peerj-10-13233-s004.zip › figure3/figure3 A/miR-NC(36 h).jpg]

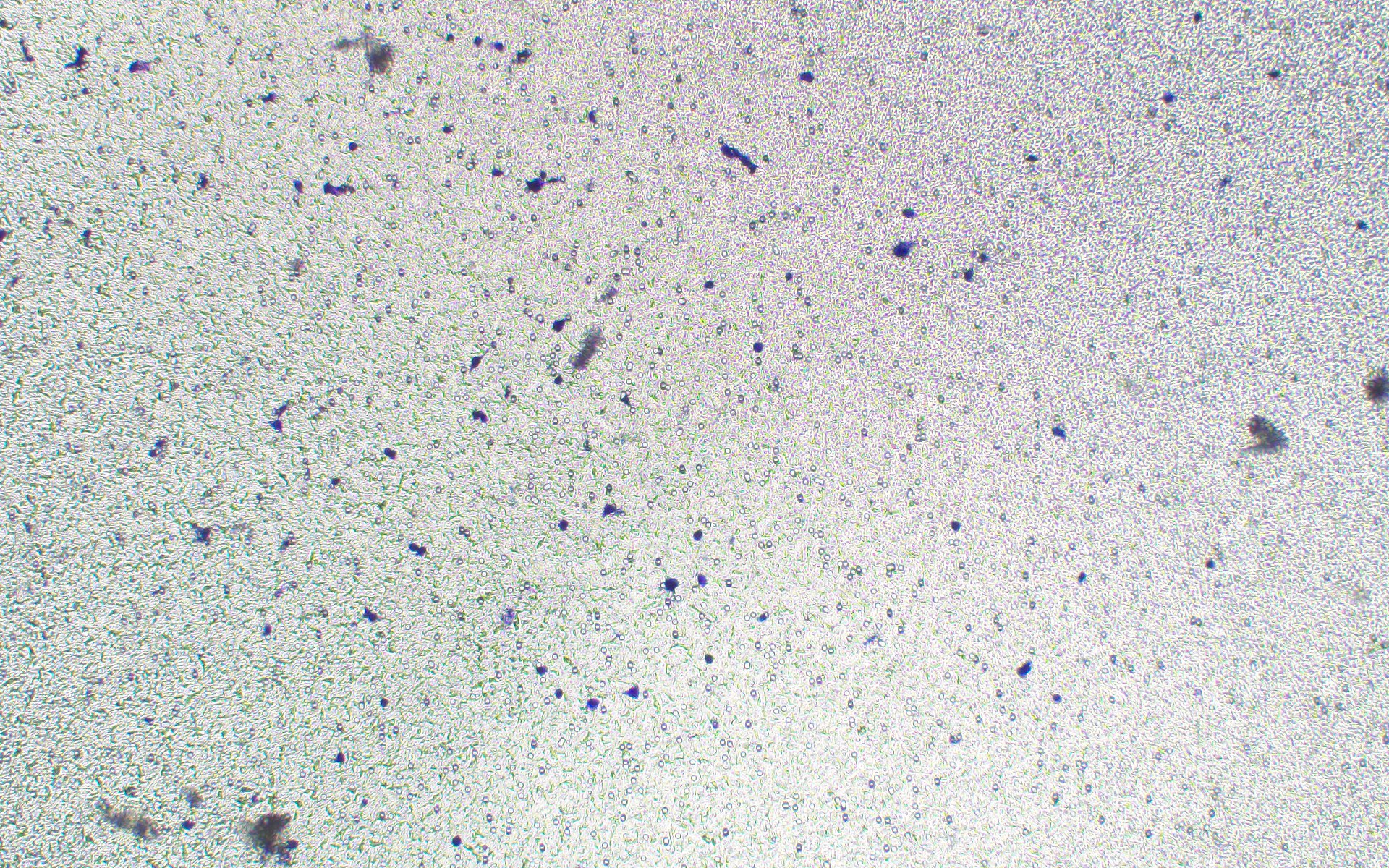

Supplement: Supplemental Information 4 [file peerj-10-13233-s004.zip › figure3/figure3 C/miR-34a-5p.jpg]

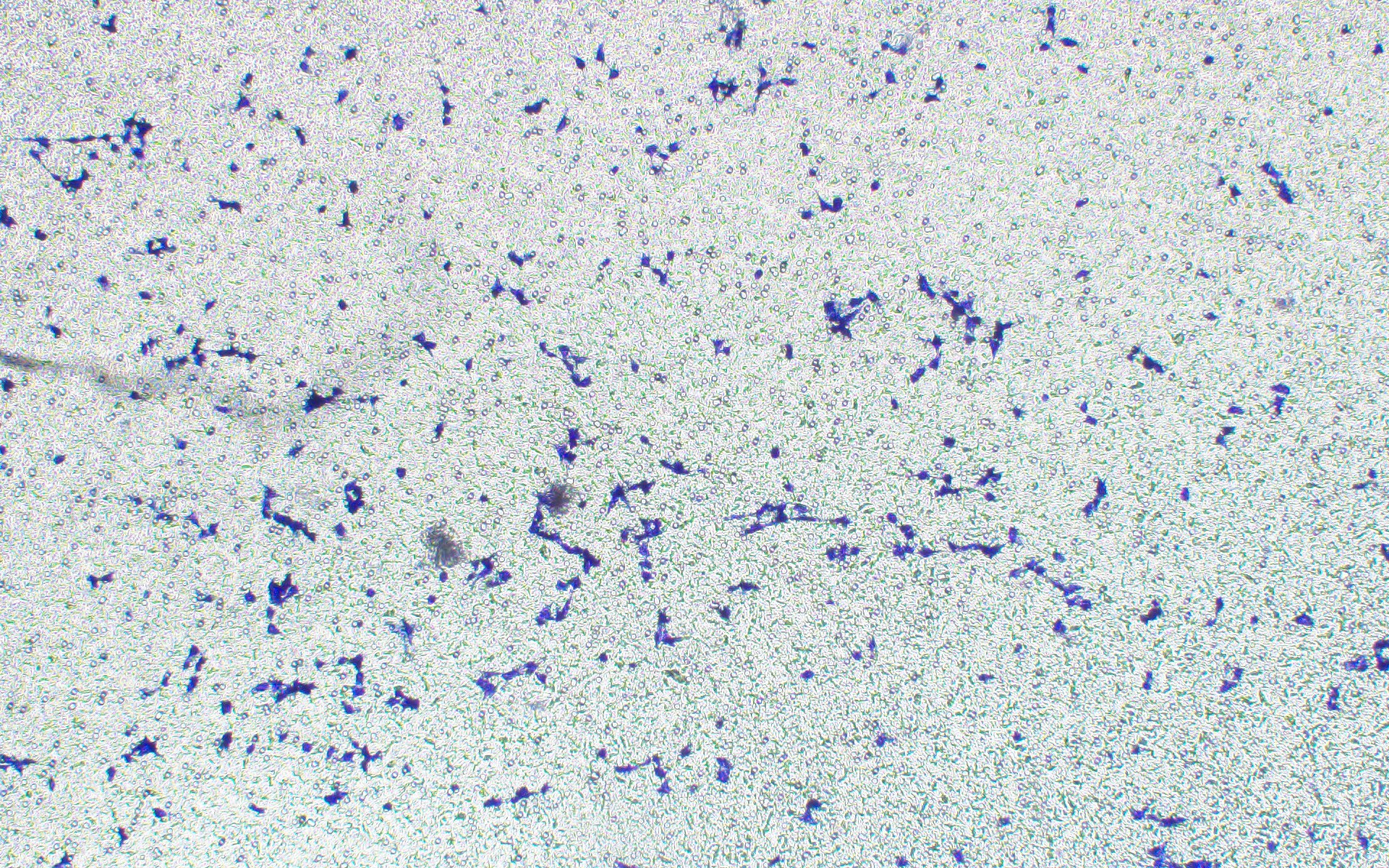

Supplement: Supplemental Information 4 [file peerj-10-13233-s004.zip › figure3/figure3 C/miR-NC.jpg]

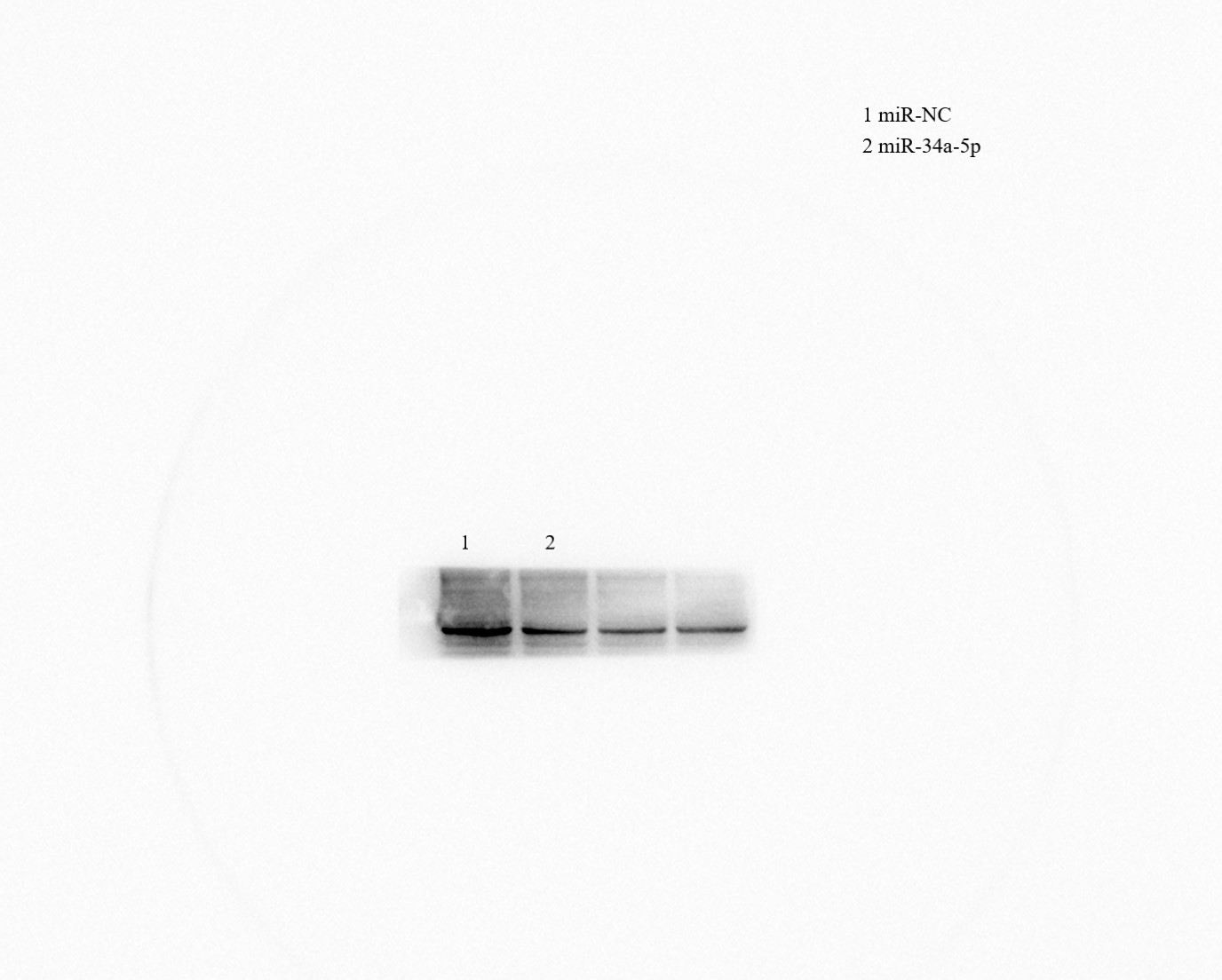

Supplement: Supplemental Information 4 [file peerj-10-13233-s004.zip › figure3/figure3 E/MMP2.jpg]

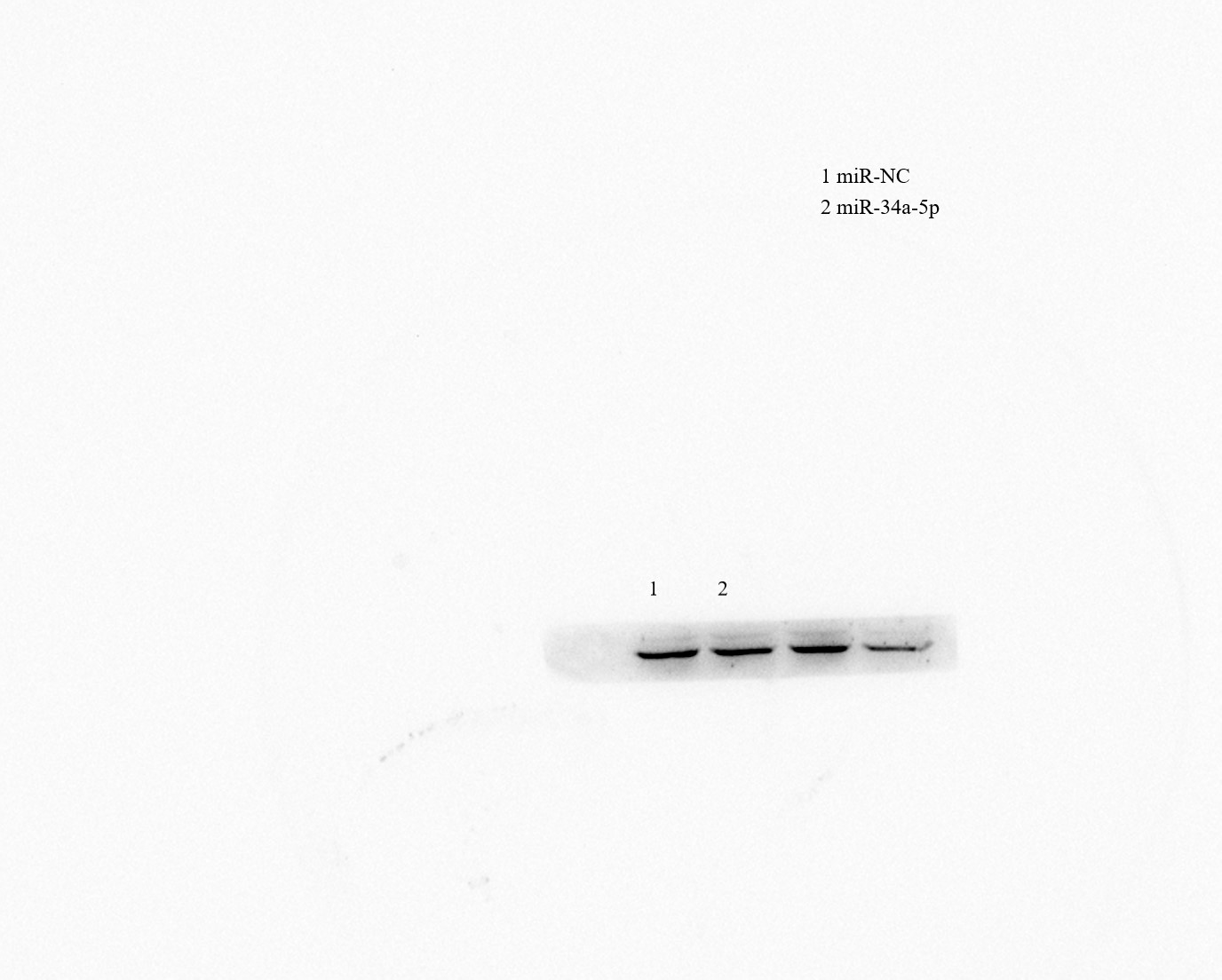

Supplement: Supplemental Information 4 [file peerj-10-13233-s004.zip › figure3/figure3 E/β-actin.jpg]

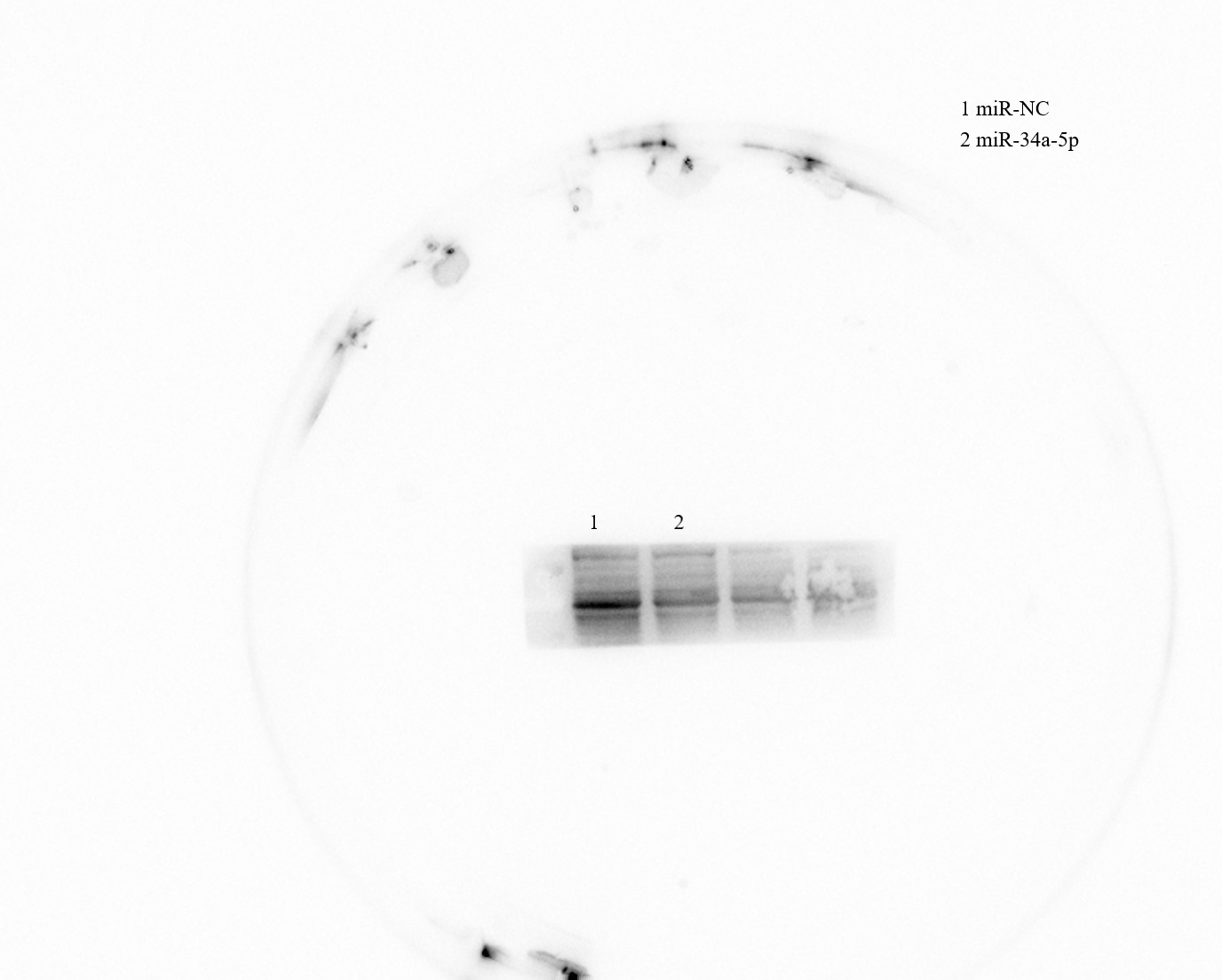

Supplement: Supplemental Information 4 [file peerj-10-13233-s004.zip › figure3/figure3 F/MMP9.jpg]

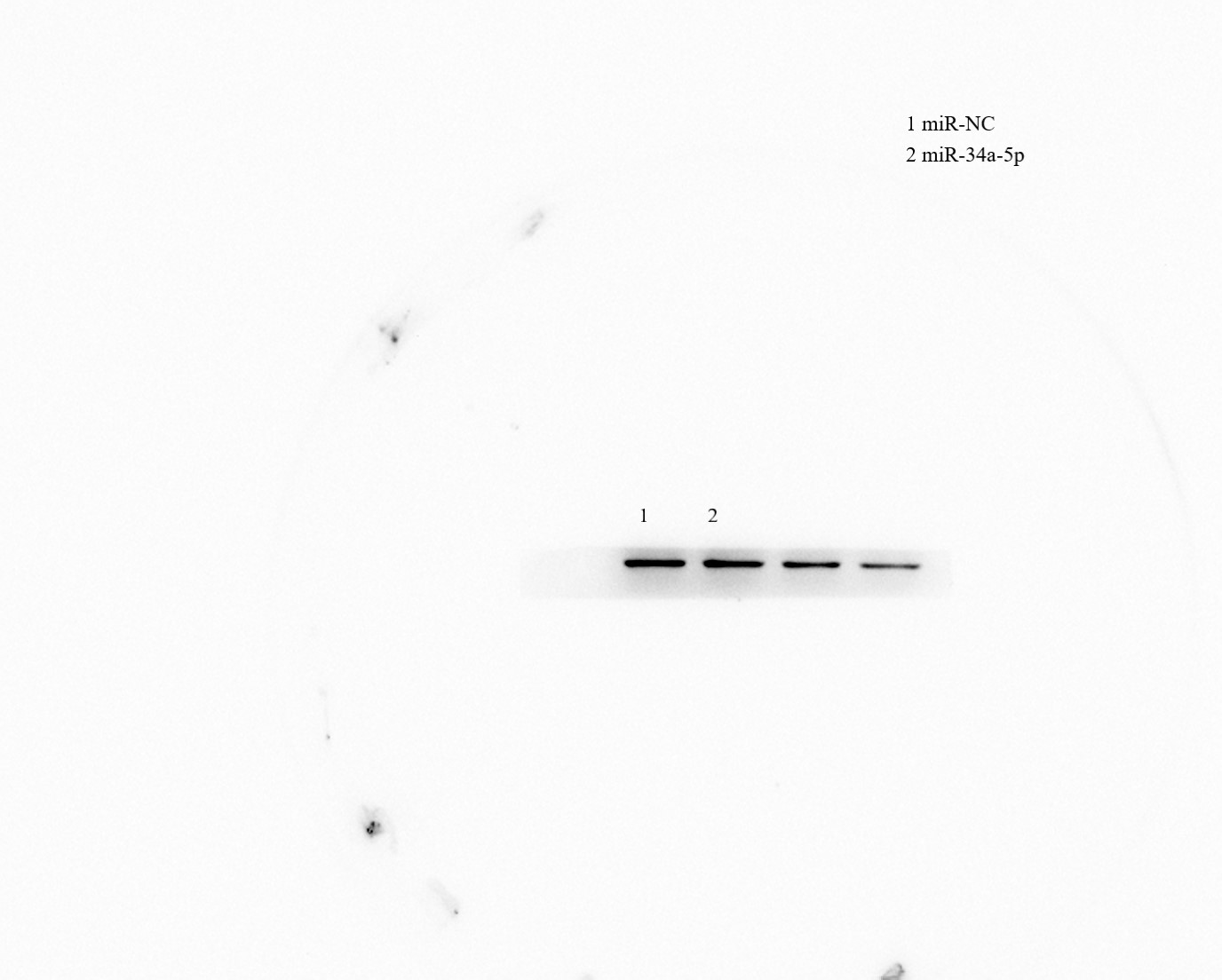

Supplement: Supplemental Information 4 [file peerj-10-13233-s004.zip › figure3/figure3 F/β-actin.jpg]

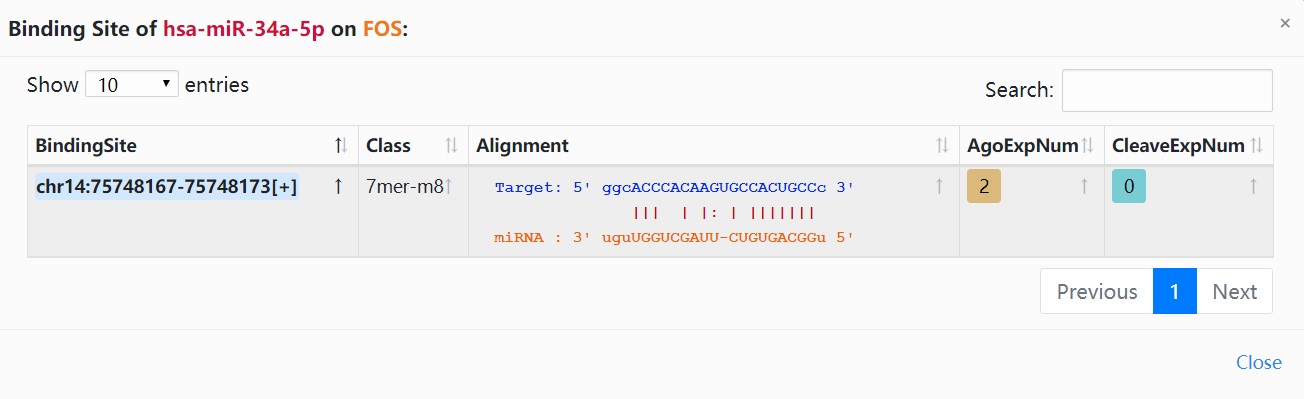

Supplement: Supplemental Information 5 [file peerj-10-13233-s005.zip › figure4/figure4 A/binding sequences .jpg]

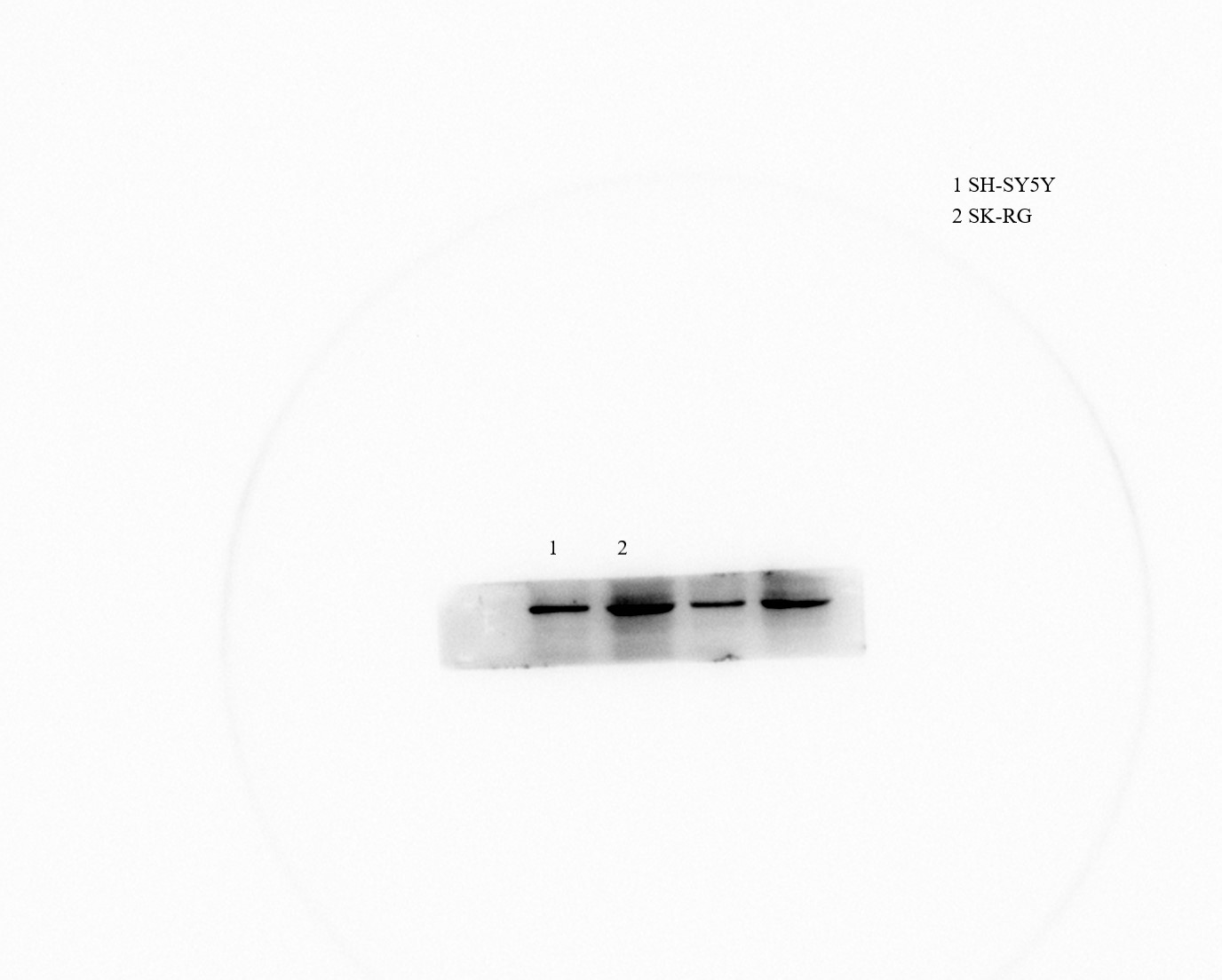

Supplement: Supplemental Information 5 [file peerj-10-13233-s005.zip › figure4/figure4 C/c-fos.jpg]

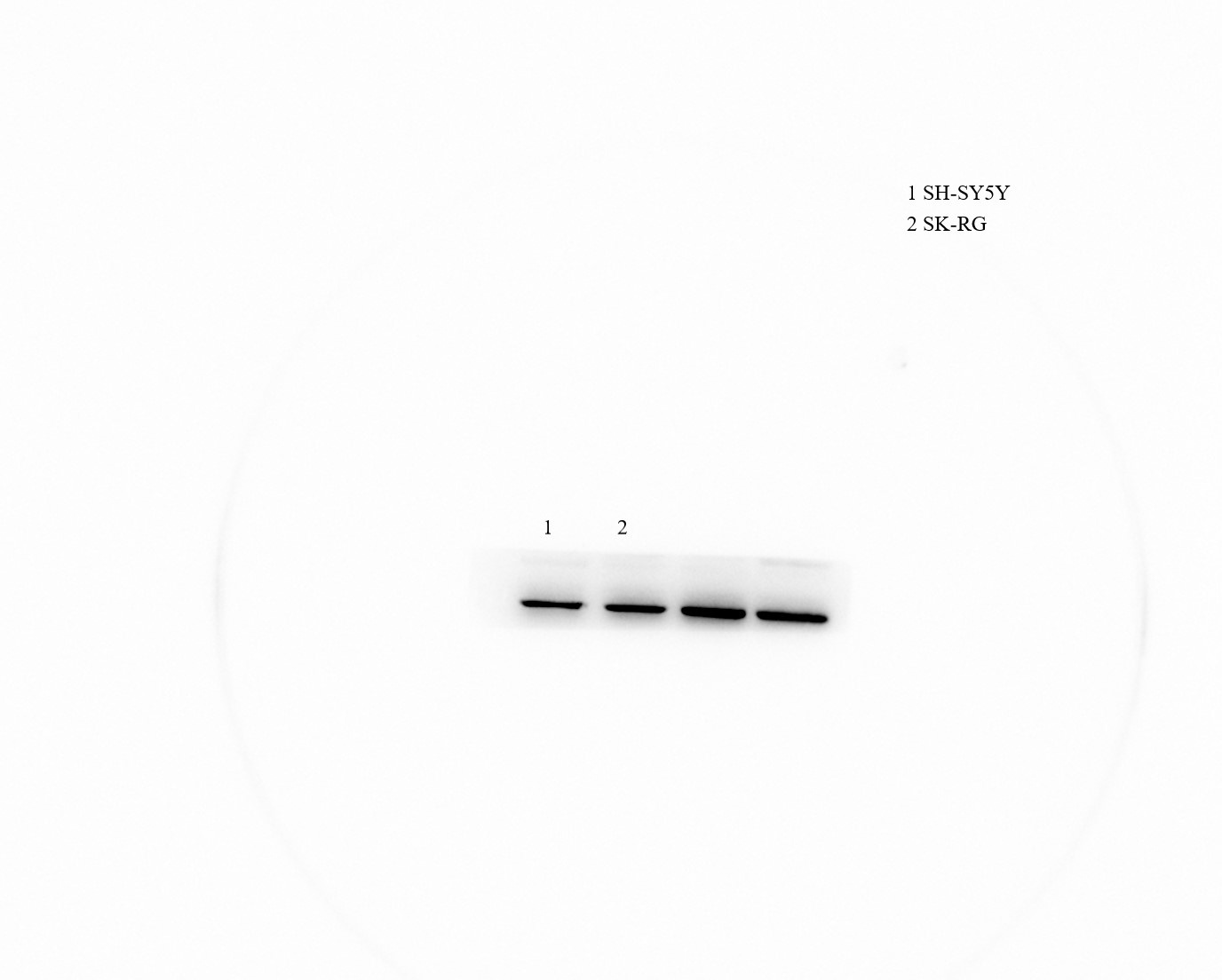

Supplement: Supplemental Information 5 [file peerj-10-13233-s005.zip › figure4/figure4 C/β-actin.jpg]

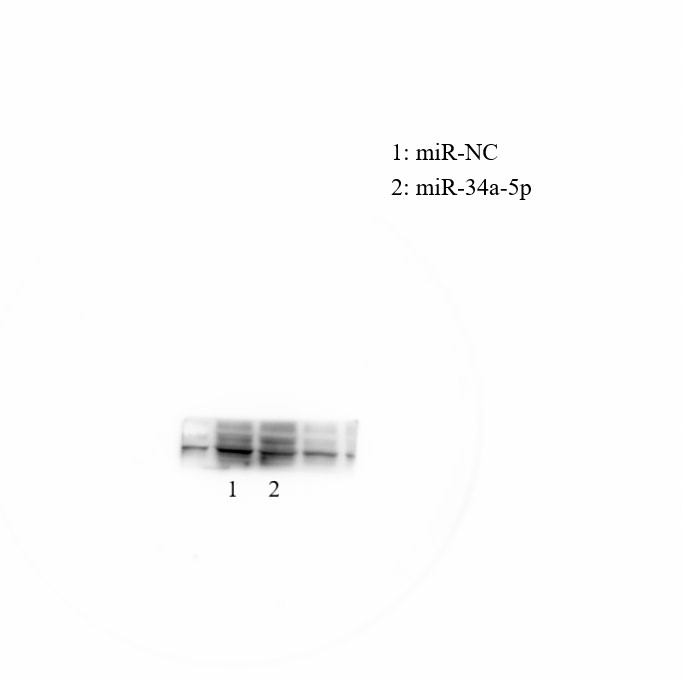

Supplement: Supplemental Information 5 [file peerj-10-13233-s005.zip › figure4/figure4 E/c-fos.jpg]

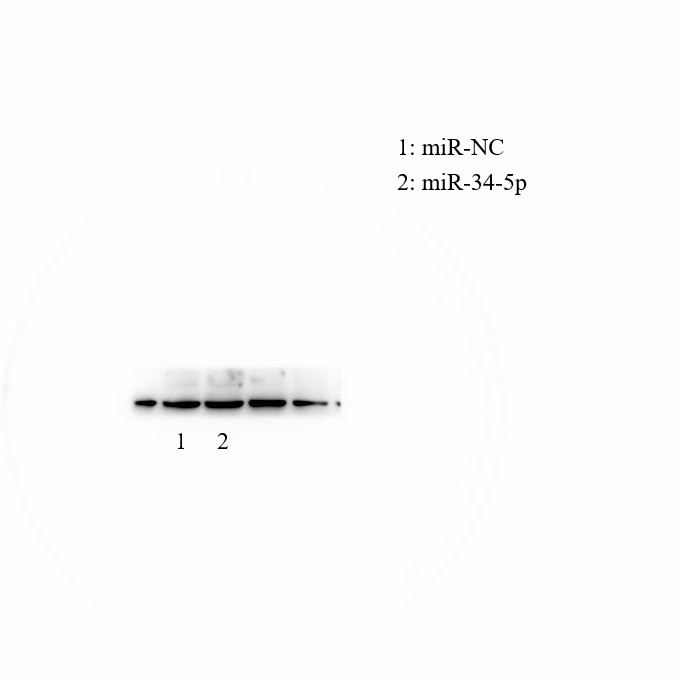

Supplement: Supplemental Information 5 [file peerj-10-13233-s005.zip › figure4/figure4 E/β-actin.jpg]

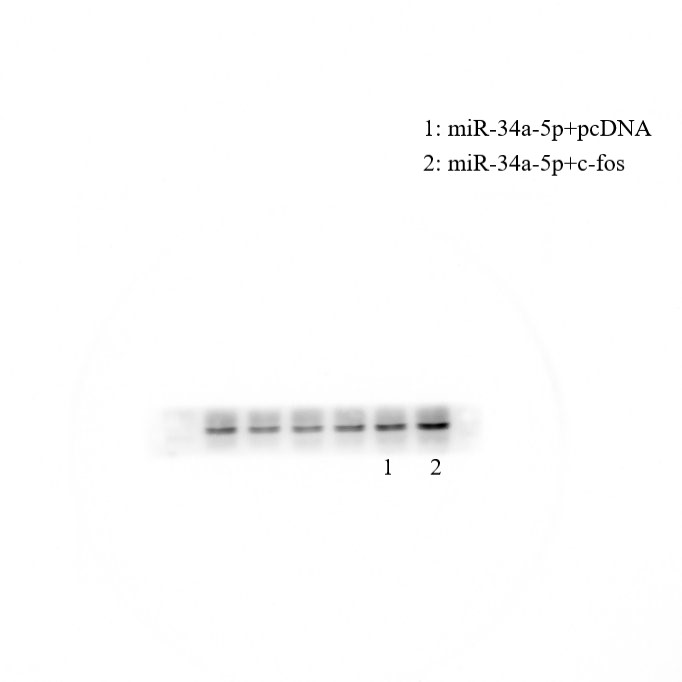

Supplement: Supplemental Information 6 [file peerj-10-13233-s006.zip › figure5/figure5 E/CDK4 .jpg]

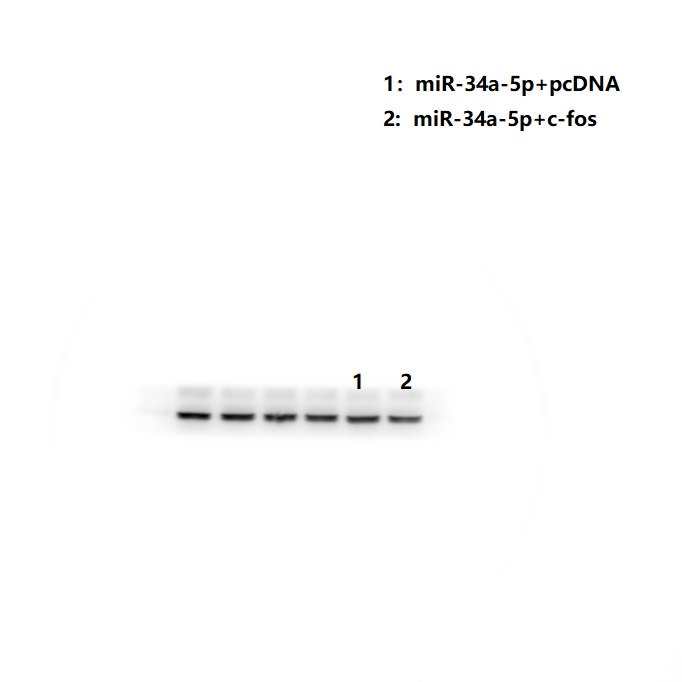

Supplement: Supplemental Information 6 [file peerj-10-13233-s006.zip › figure5/figure5 E/a┬-actin .jpg]

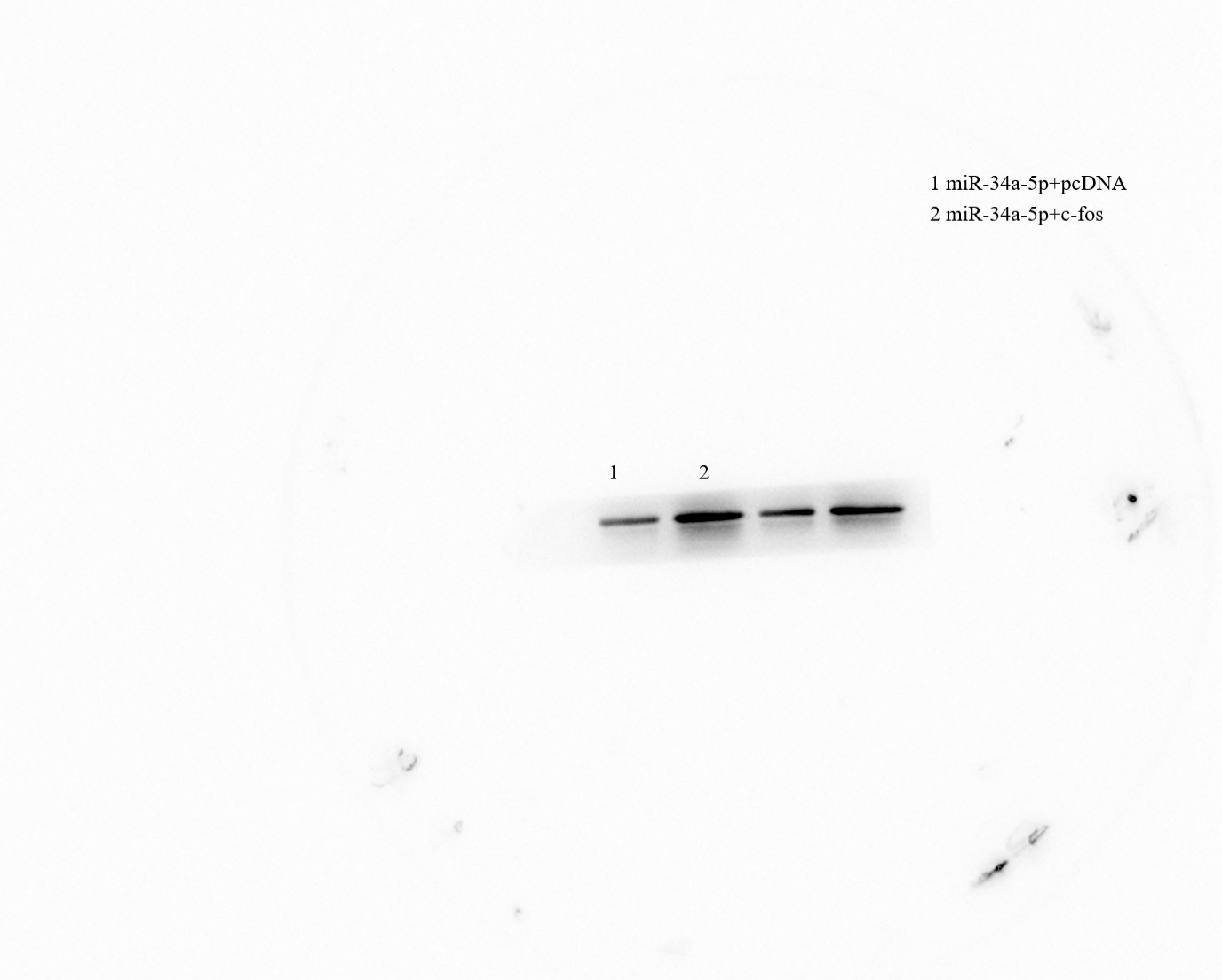

Supplement: Supplemental Information 6 [file peerj-10-13233-s006.zip › figure5/figure5 F/CDK6.jpg]

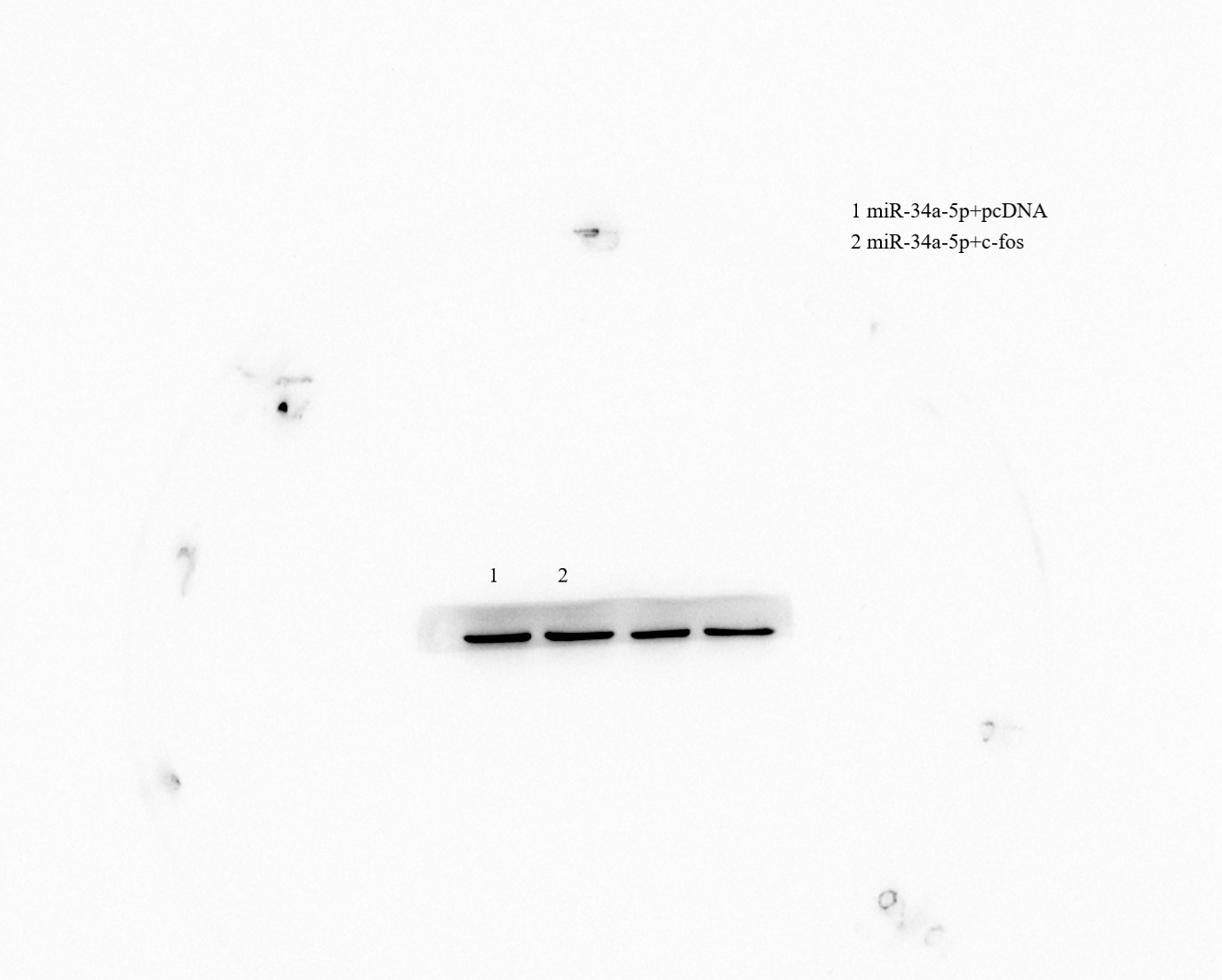

Supplement: Supplemental Information 6 [file peerj-10-13233-s006.zip › figure5/figure5 F/a┬-actin.jpg]

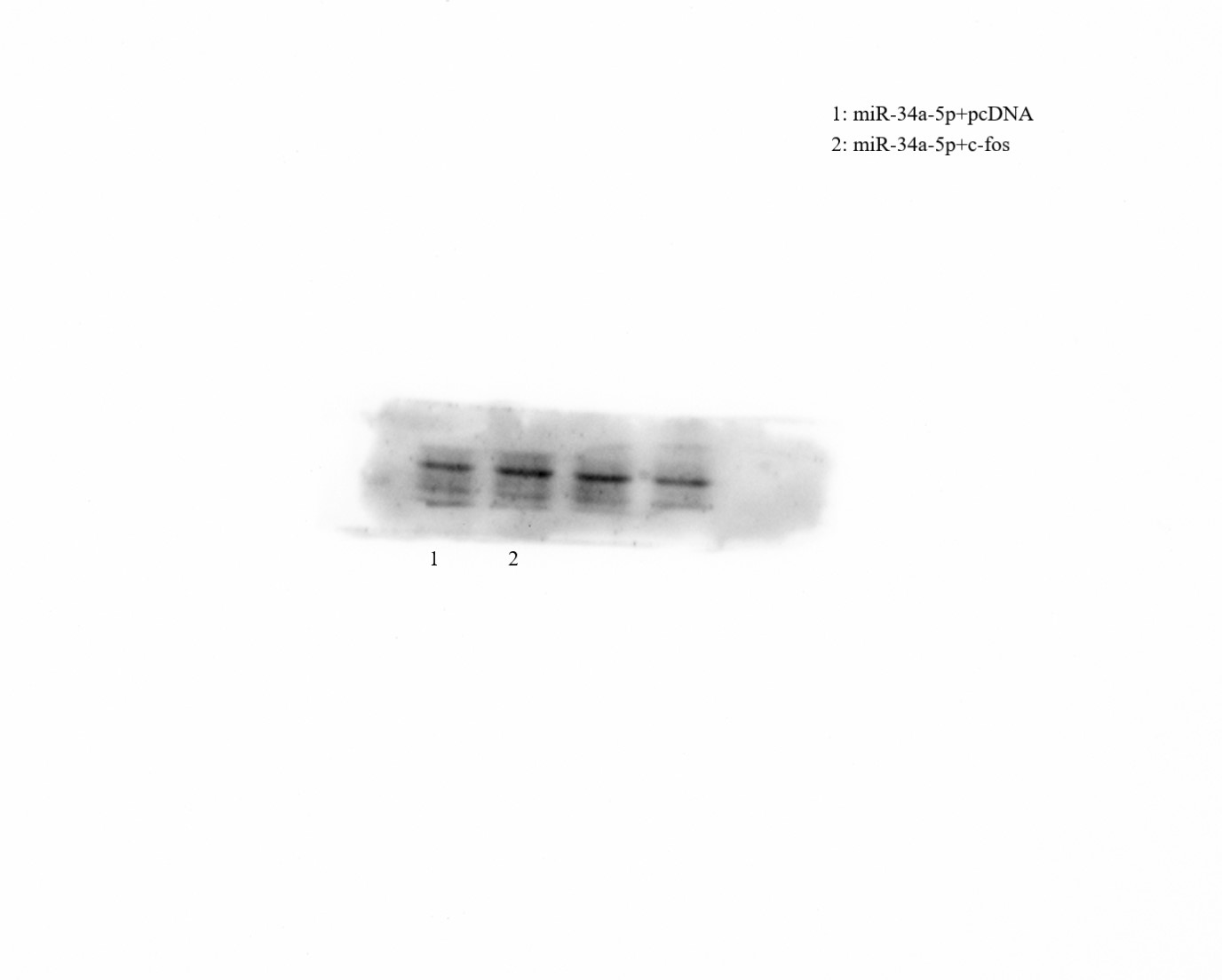

Supplement: Supplemental Information 6 [file peerj-10-13233-s006.zip › figure5/figure5 G/cyclin D1.jpg]

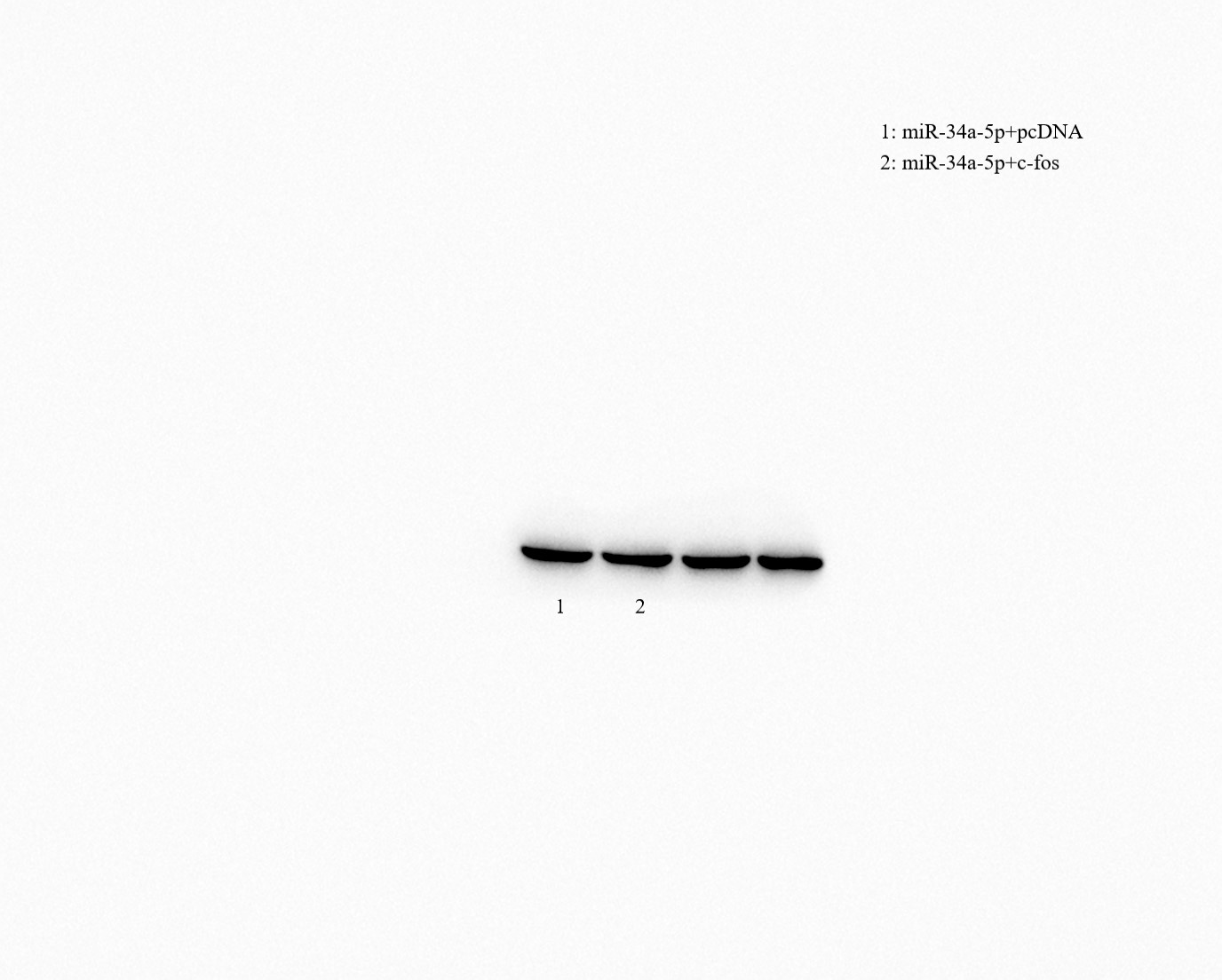

Supplement: Supplemental Information 6 [file peerj-10-13233-s006.zip › figure5/figure5 G/a┬-actin.jpg]

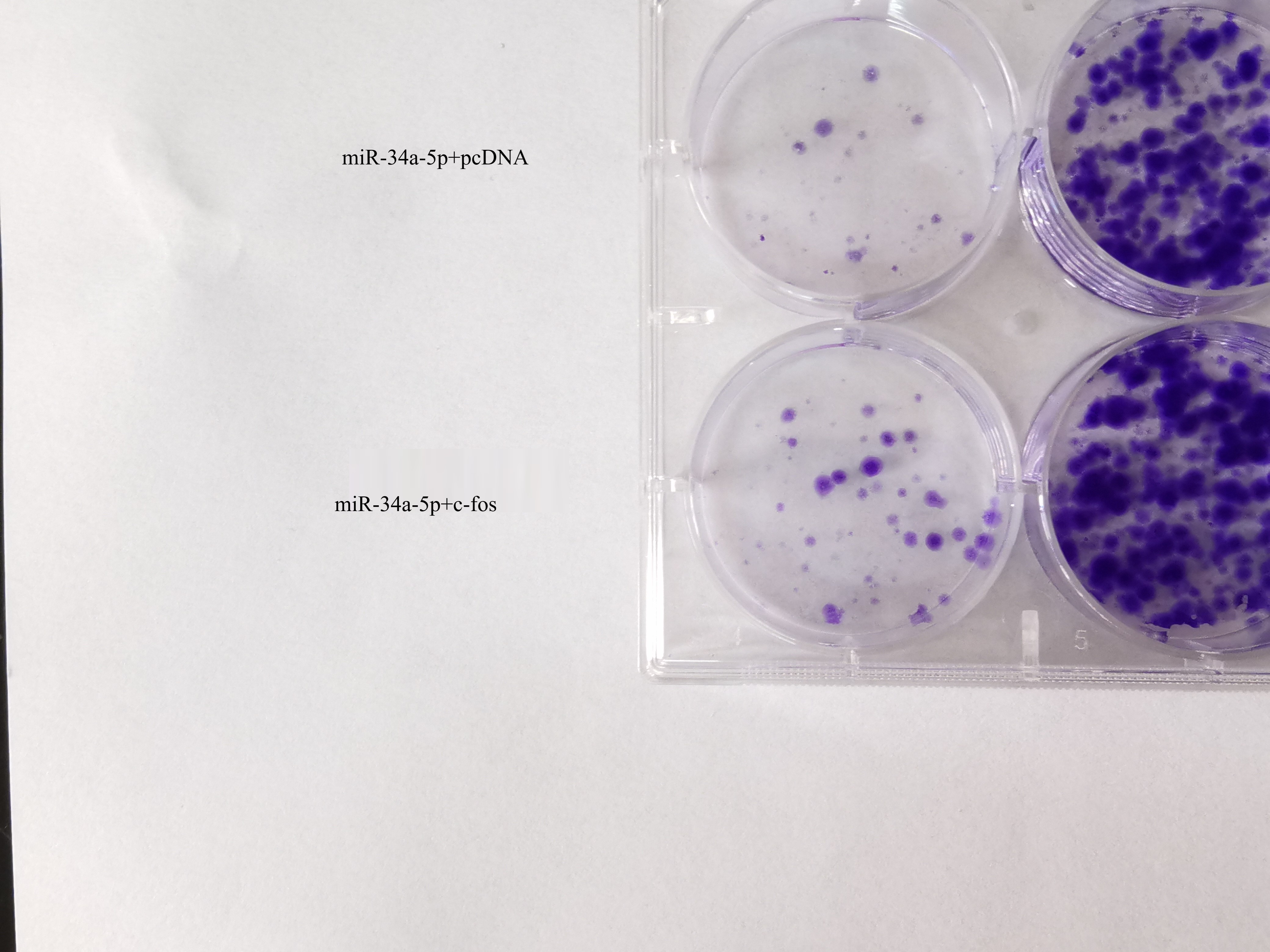

Supplement: Supplemental Information 6 [file peerj-10-13233-s006.zip › figure5/figure5 H/Plate clone.jpg]

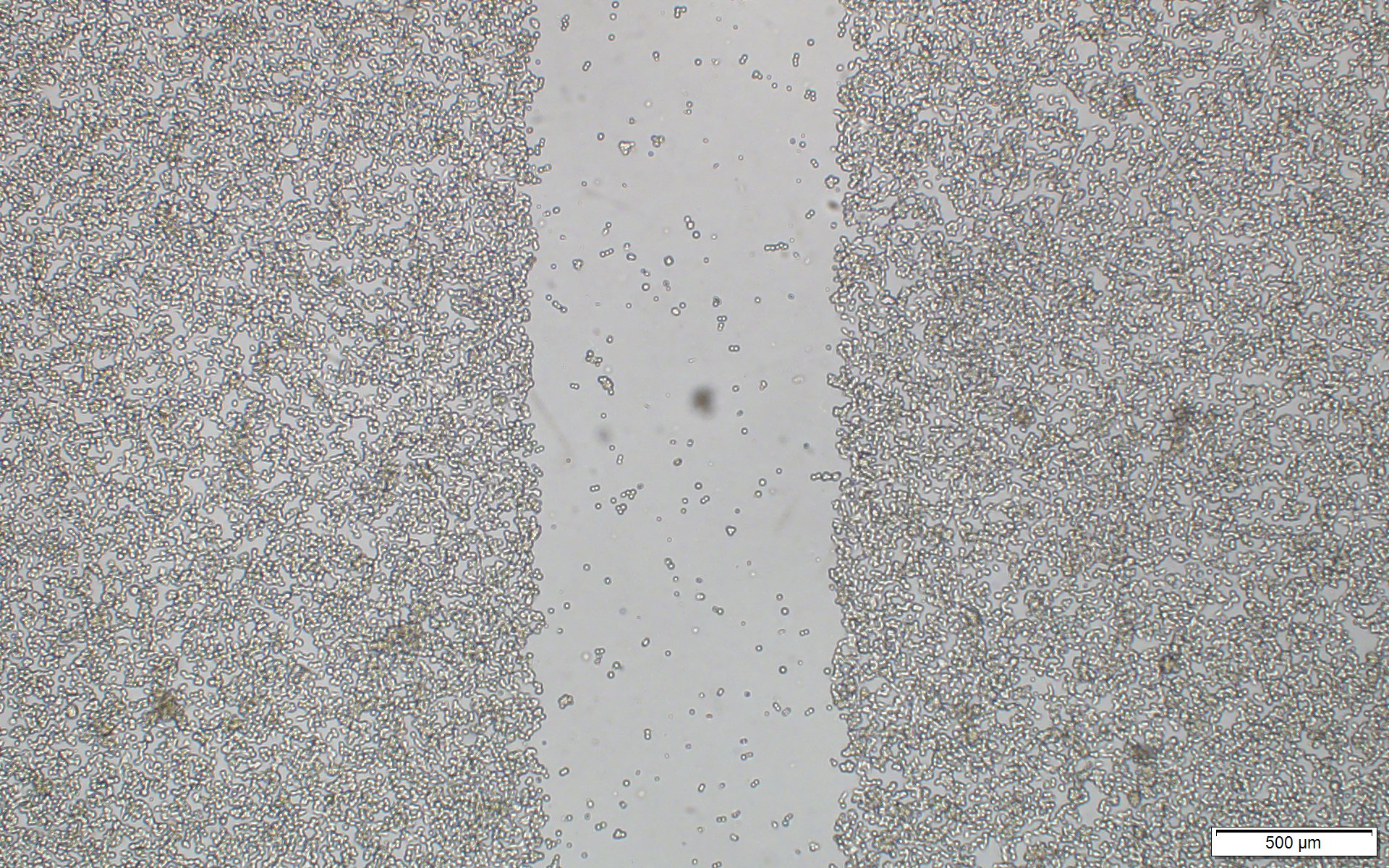

Supplement: Supplemental Information 7 [file peerj-10-13233-s007.zip › figure6/figure6 A/miR-34a-5p+c-fos(0h).jpg]

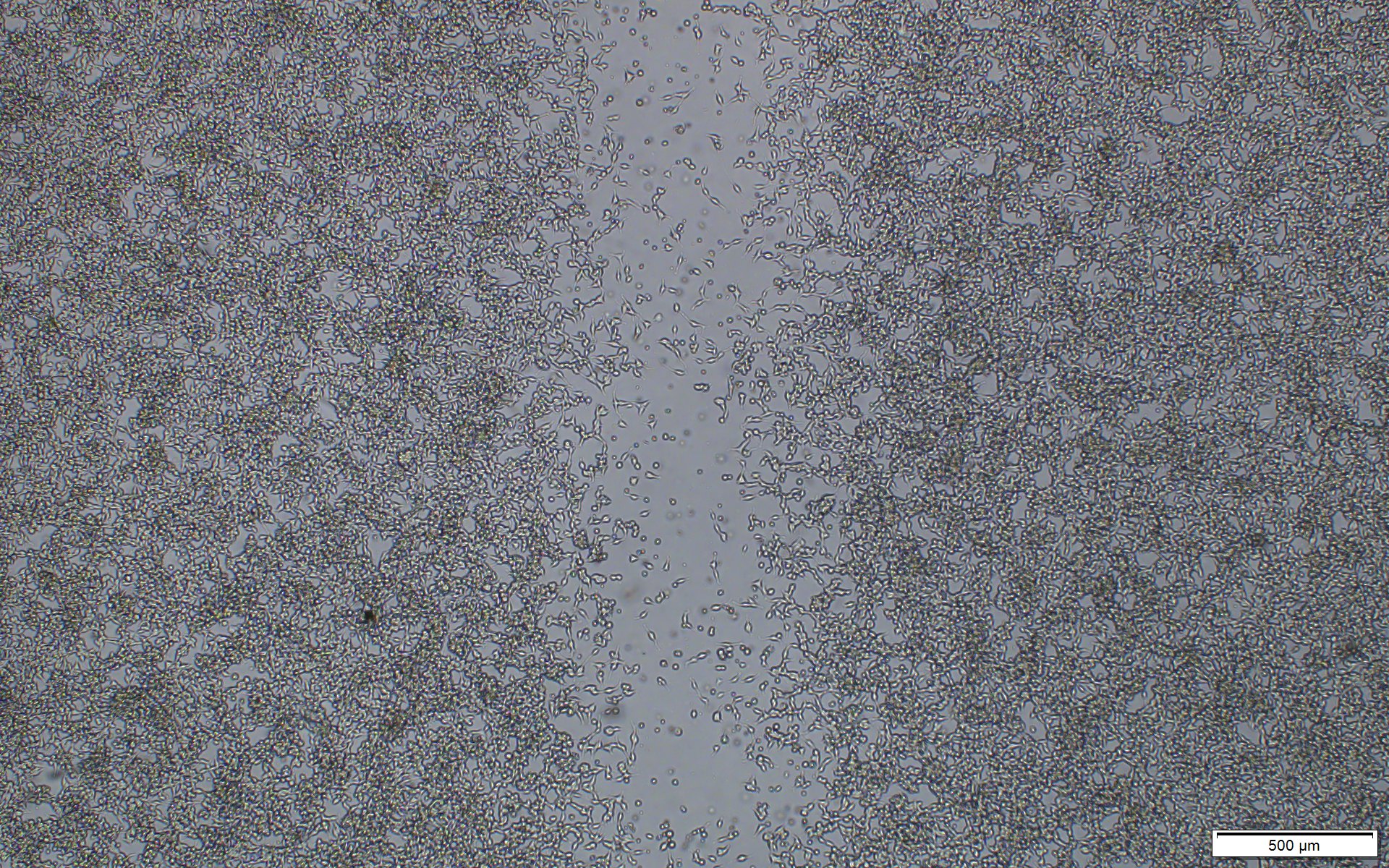

Supplement: Supplemental Information 7 [file peerj-10-13233-s007.zip › figure6/figure6 A/miR-34a-5p+c-fos(36h).jpg]

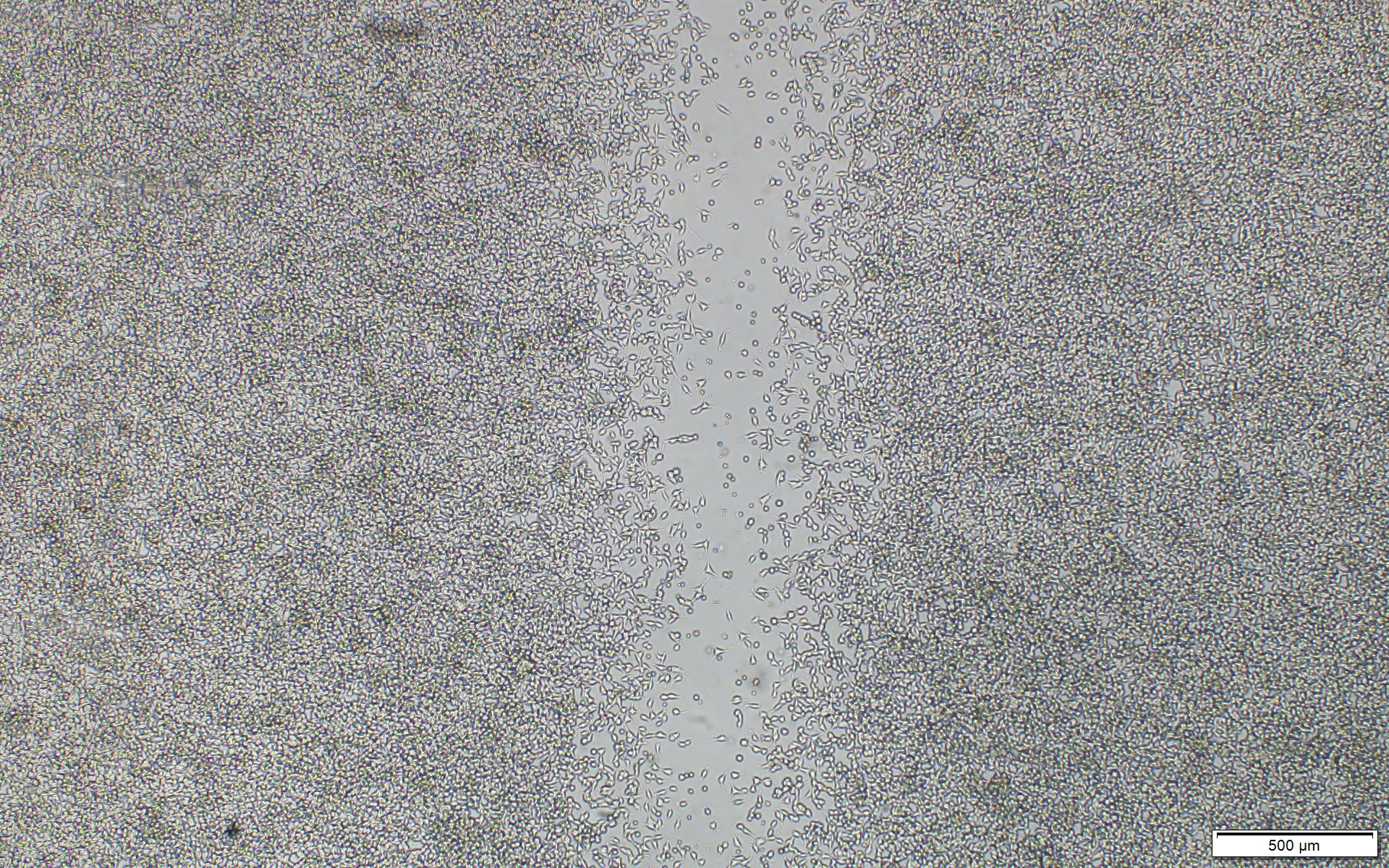

Supplement: Supplemental Information 7 [file peerj-10-13233-s007.zip › figure6/figure6 A/miR-34a-5p+c-fos(48h).jpg]

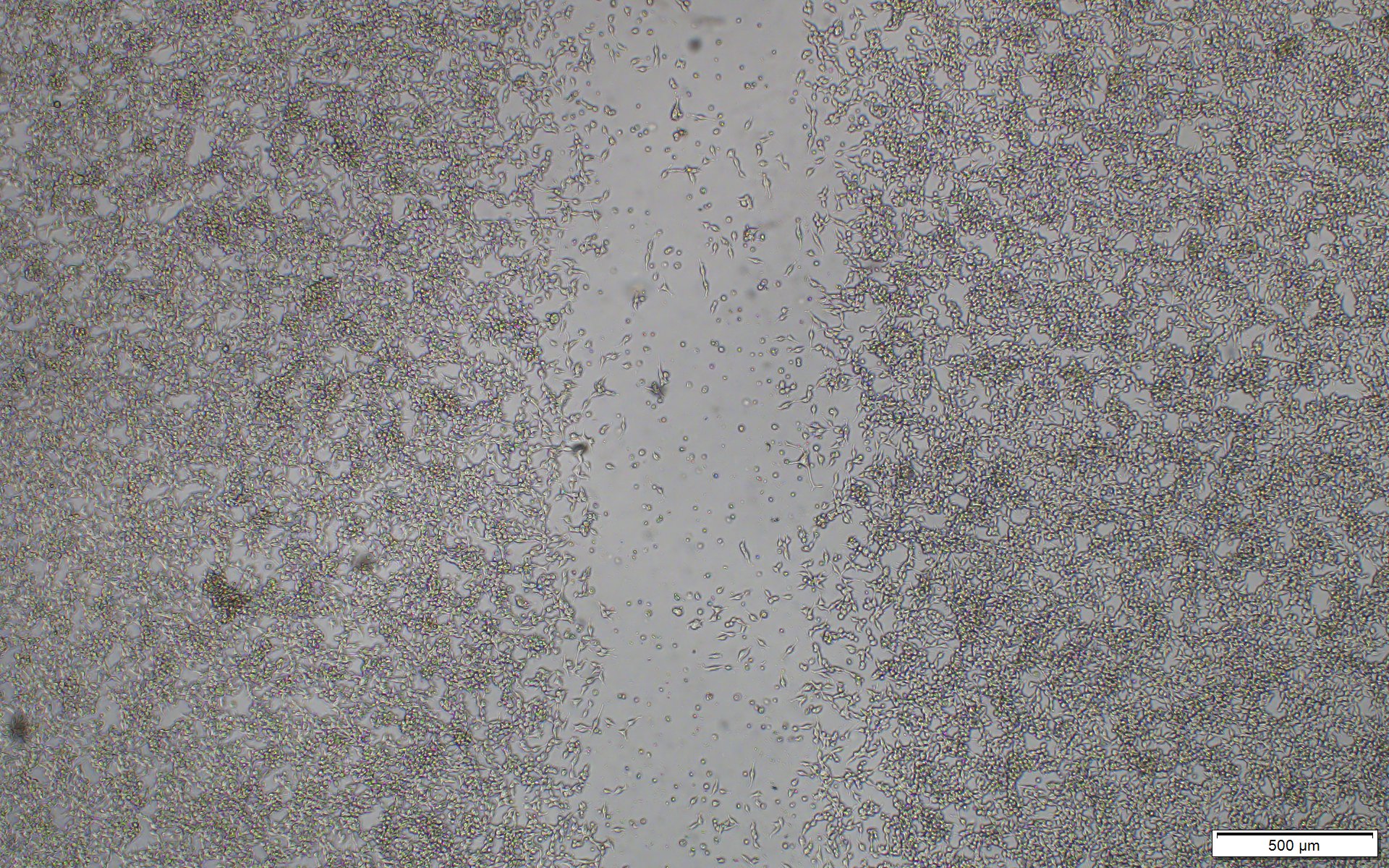

Supplement: Supplemental Information 7 [file peerj-10-13233-s007.zip › figure6/figure6 A/miR-34a-5p+pcDNA(36h).jpg]

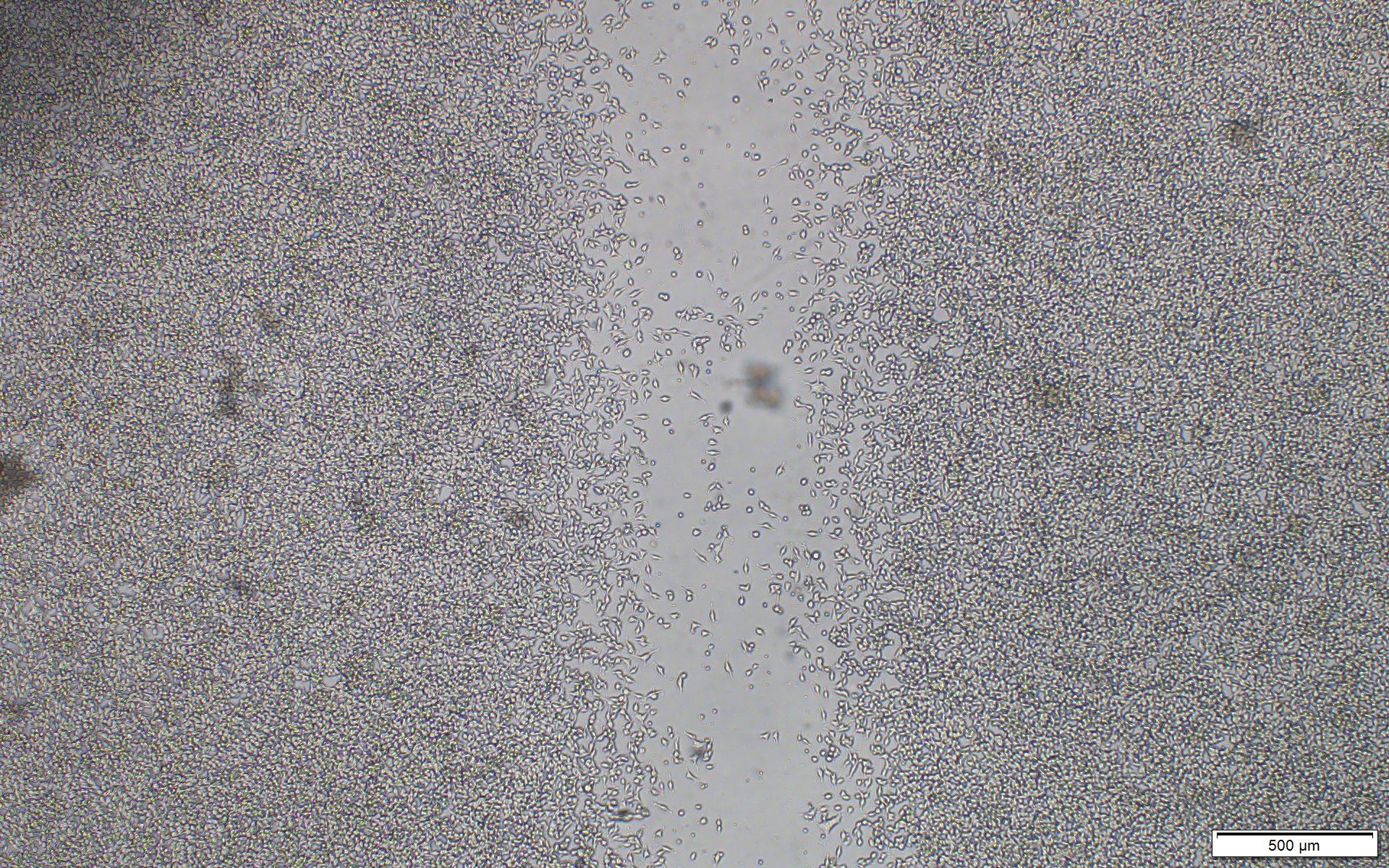

Supplement: Supplemental Information 7 [file peerj-10-13233-s007.zip › figure6/figure6 A/miR-34a-5p+pcDNA(48h).jpg]

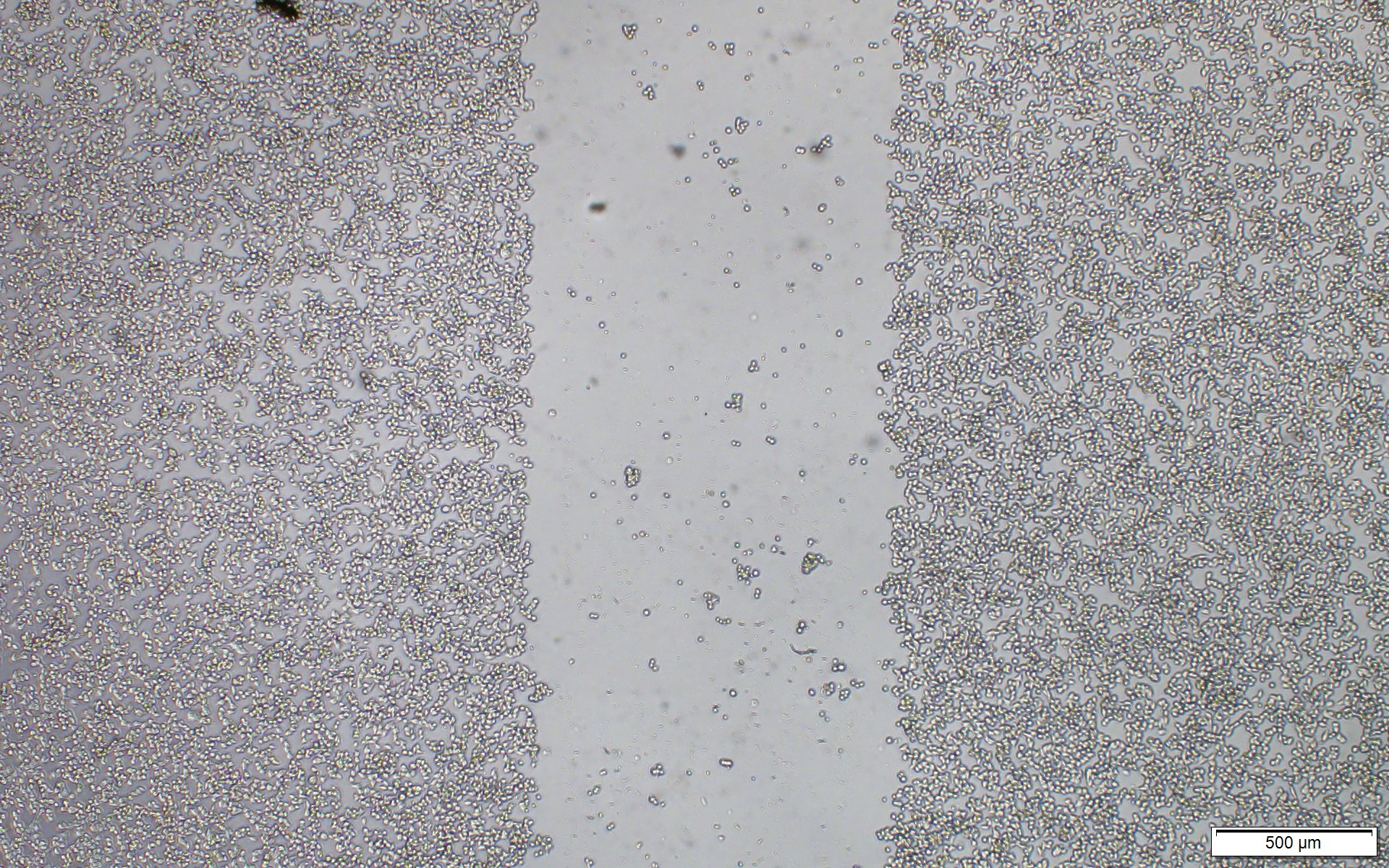

Supplement: Supplemental Information 7 [file peerj-10-13233-s007.zip › figure6/figure6 A/miR-34a-5p+pcDNA(0h).jpg]

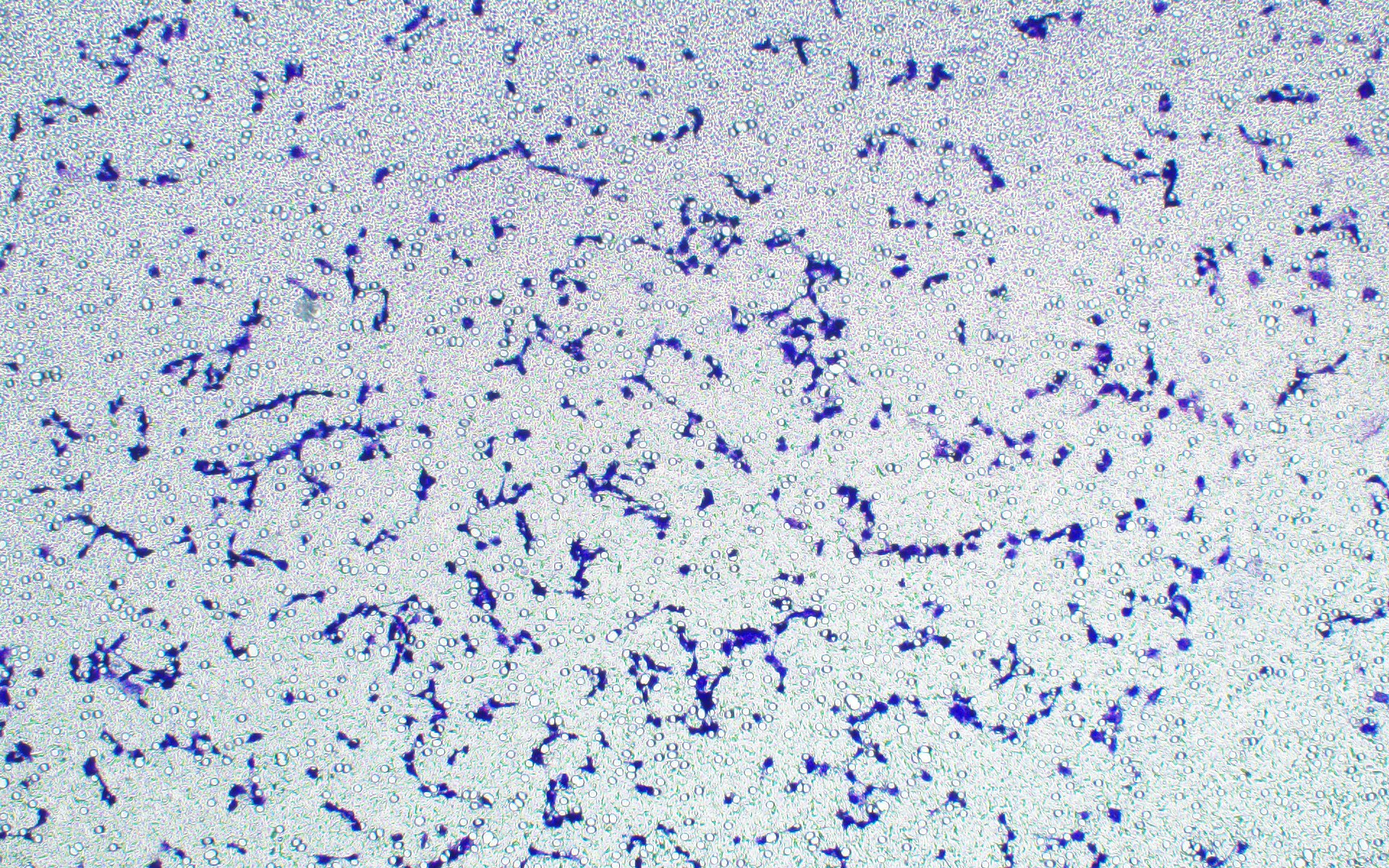

Supplement: Supplemental Information 7 [file peerj-10-13233-s007.zip › figure6/figure6 C/miR-34a-5p+c-fos.jpg]

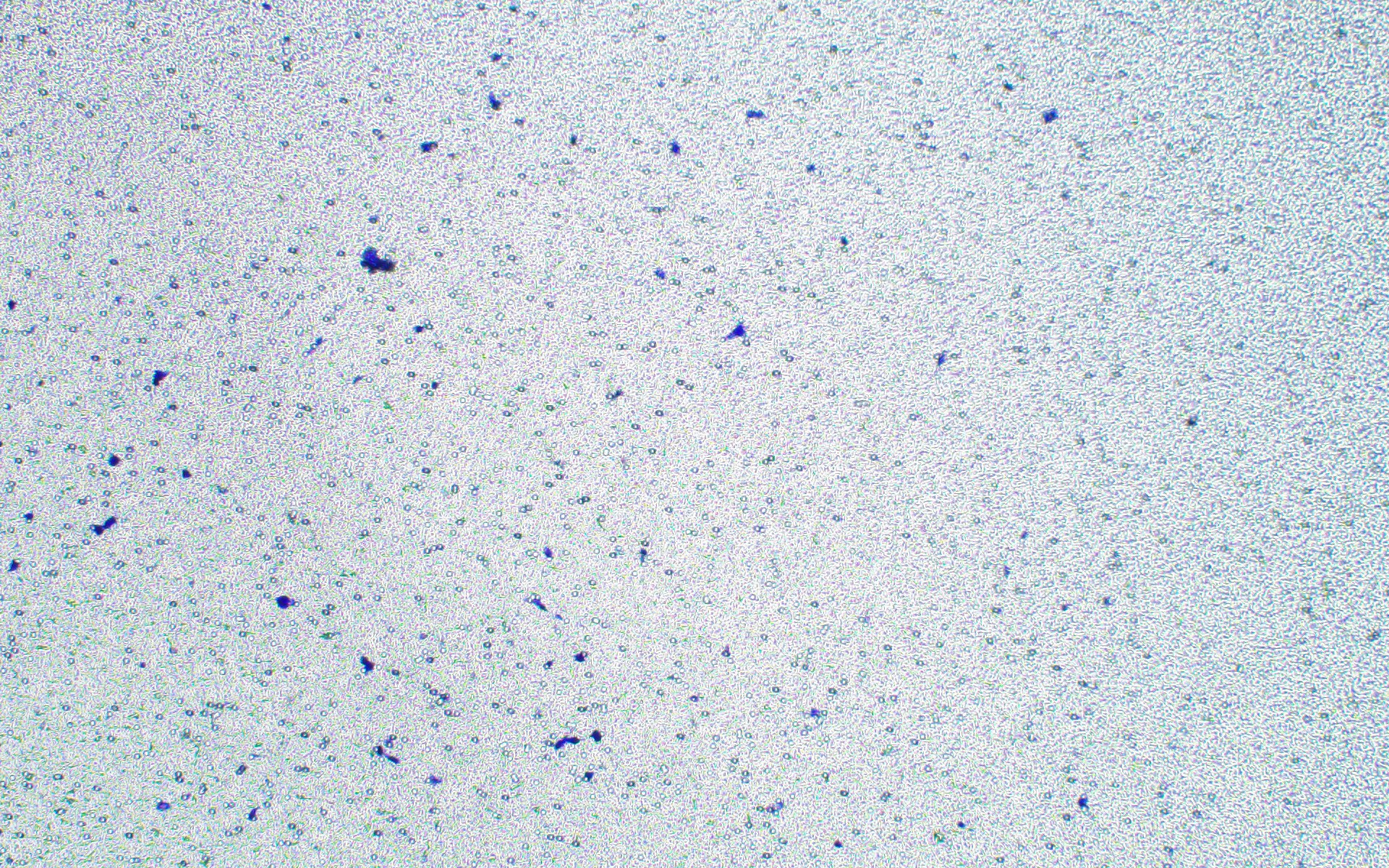

Supplement: Supplemental Information 7 [file peerj-10-13233-s007.zip › figure6/figure6 C/miR-34a-5p+pcDNA.jpg]

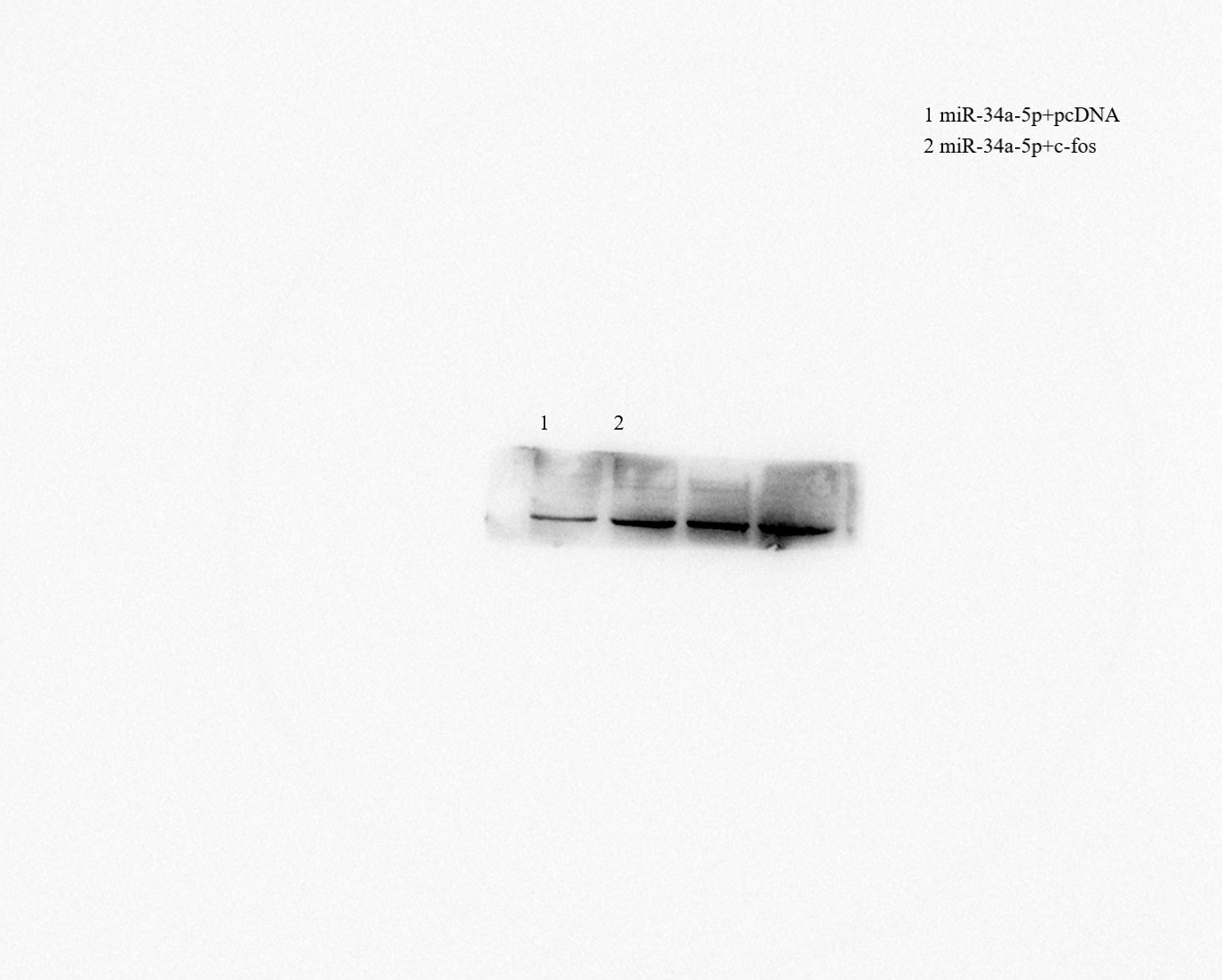

Supplement: Supplemental Information 7 [file peerj-10-13233-s007.zip › figure6/figure6 E/MMP2.jpg]

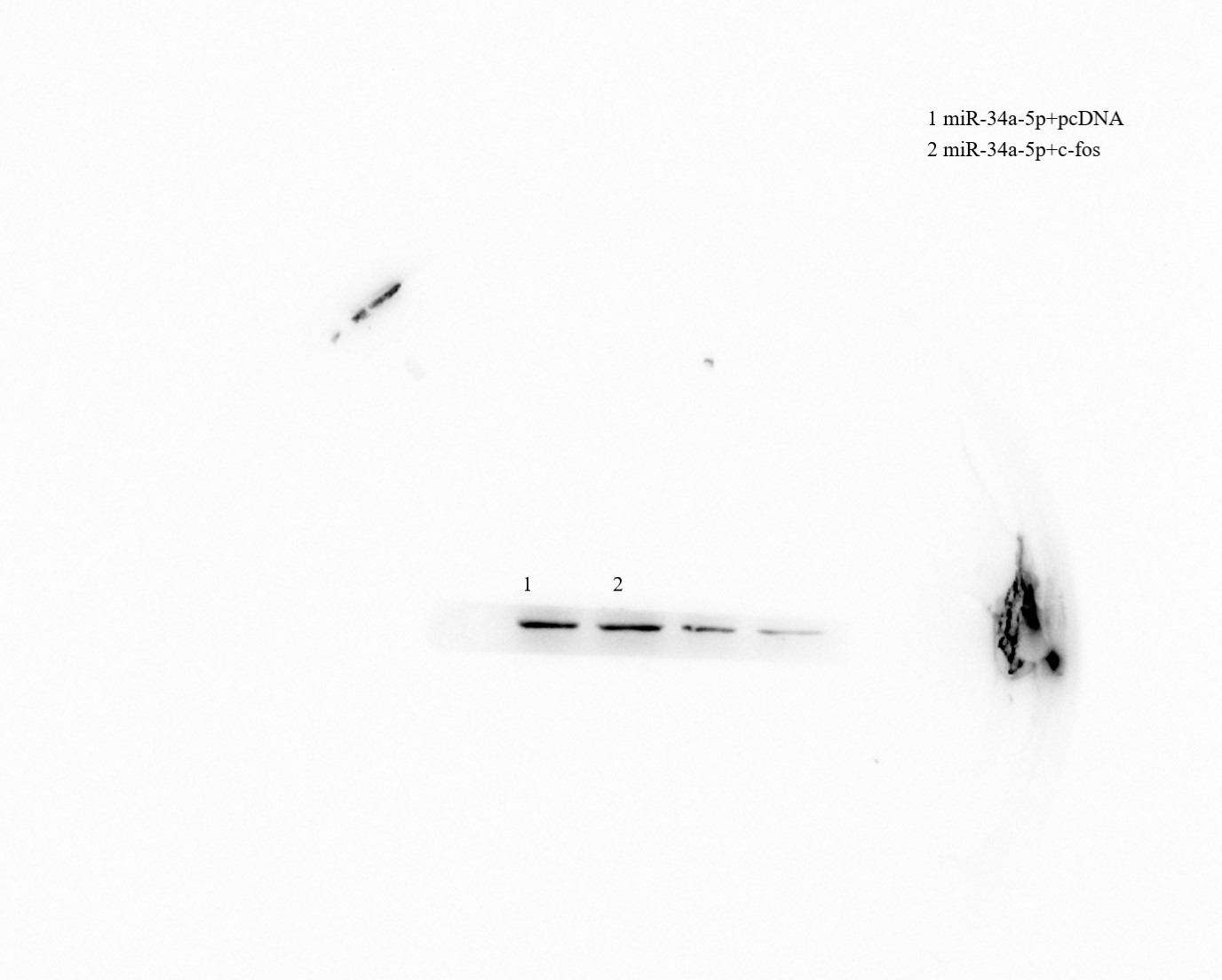

Supplement: Supplemental Information 7 [file peerj-10-13233-s007.zip › figure6/figure6 E/β-actin.jpg]

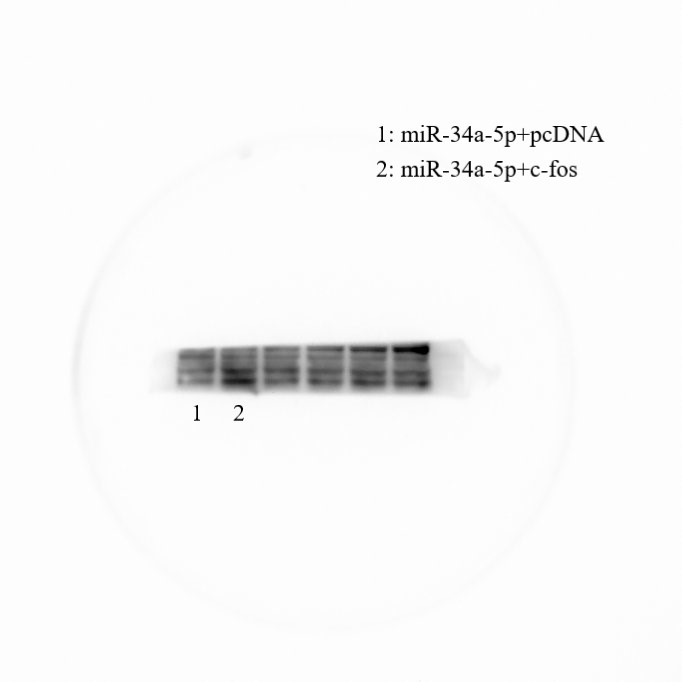

Supplement: Supplemental Information 7 [file peerj-10-13233-s007.zip › figure6/figure6 F/MMP9.jpg]

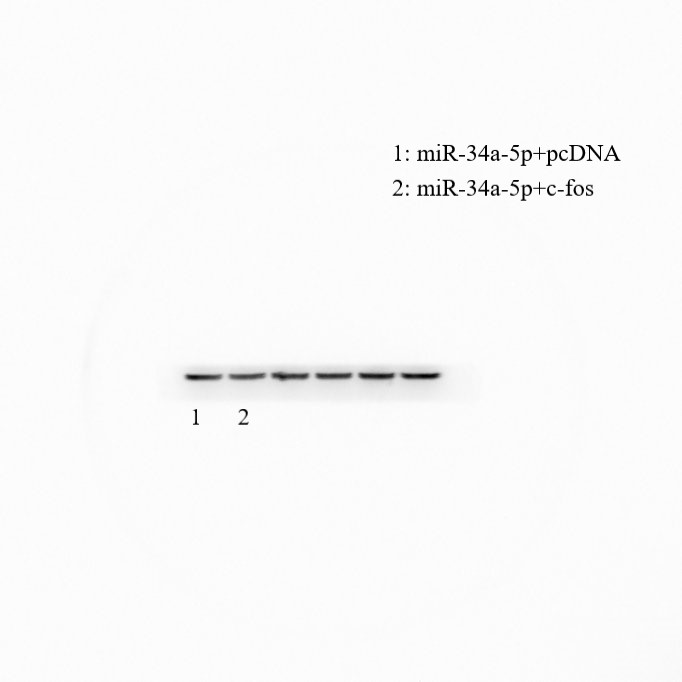

Supplement: Supplemental Information 7 [file peerj-10-13233-s007.zip › figure6/figure6 F/β-actin.jpg]

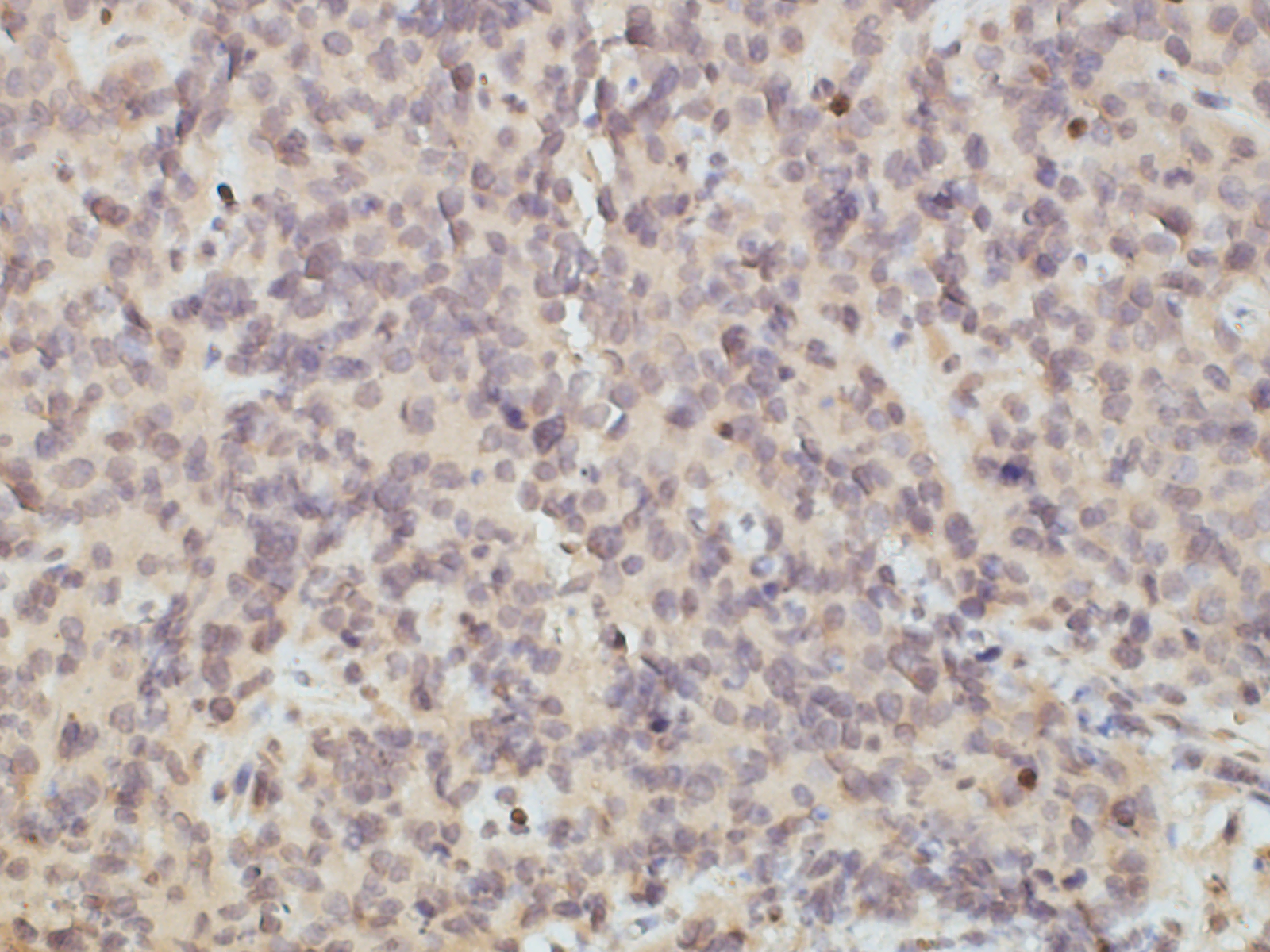

Supplement: Supplemental Information 9 [file peerj-10-13233-s009.zip › figure8/figure8 E/c-fos/miR-34a-5p.tif]

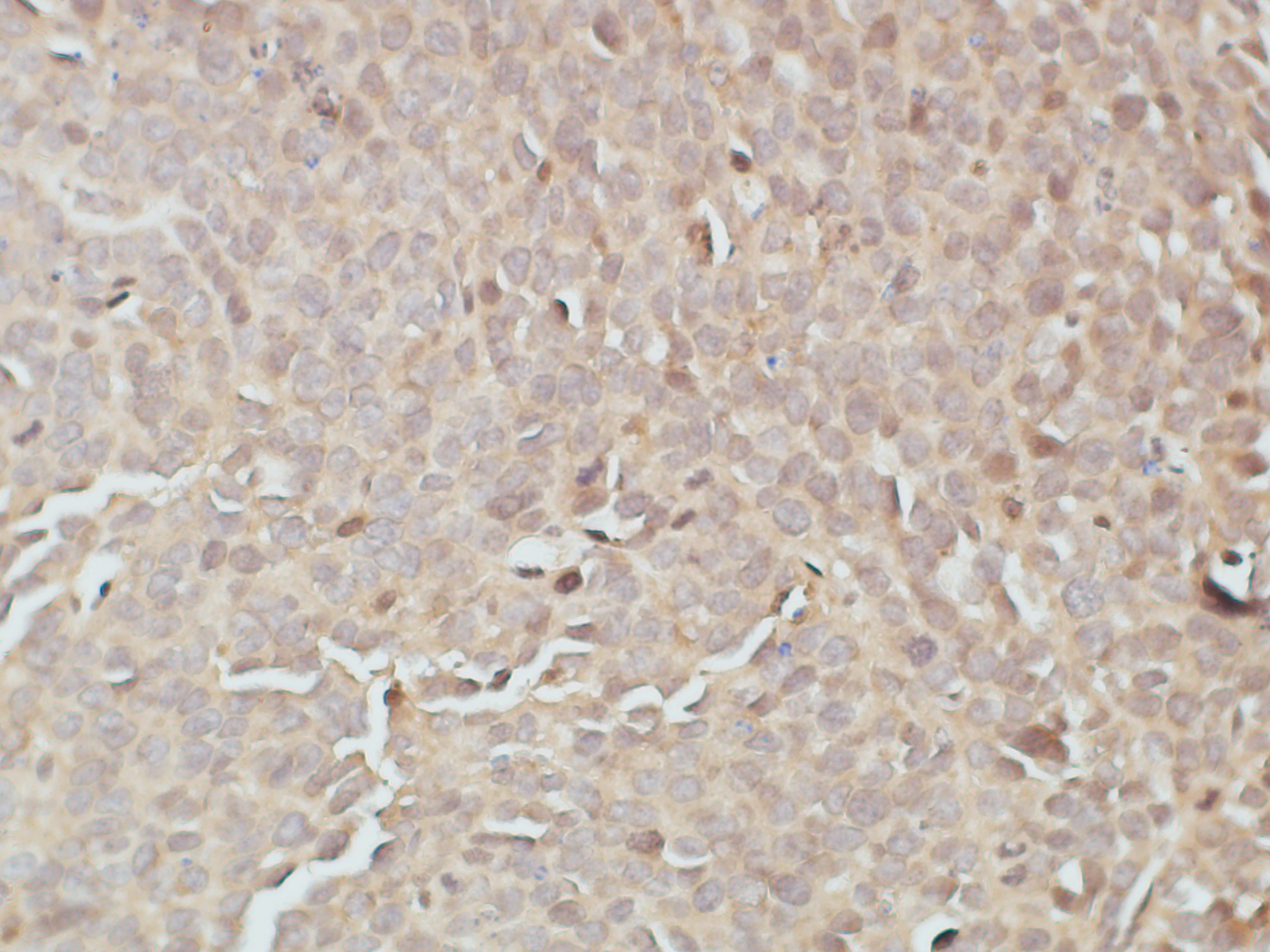

Supplement: Supplemental Information 9 [file peerj-10-13233-s009.zip › figure8/figure8 E/c-fos/miR-NC.tif]

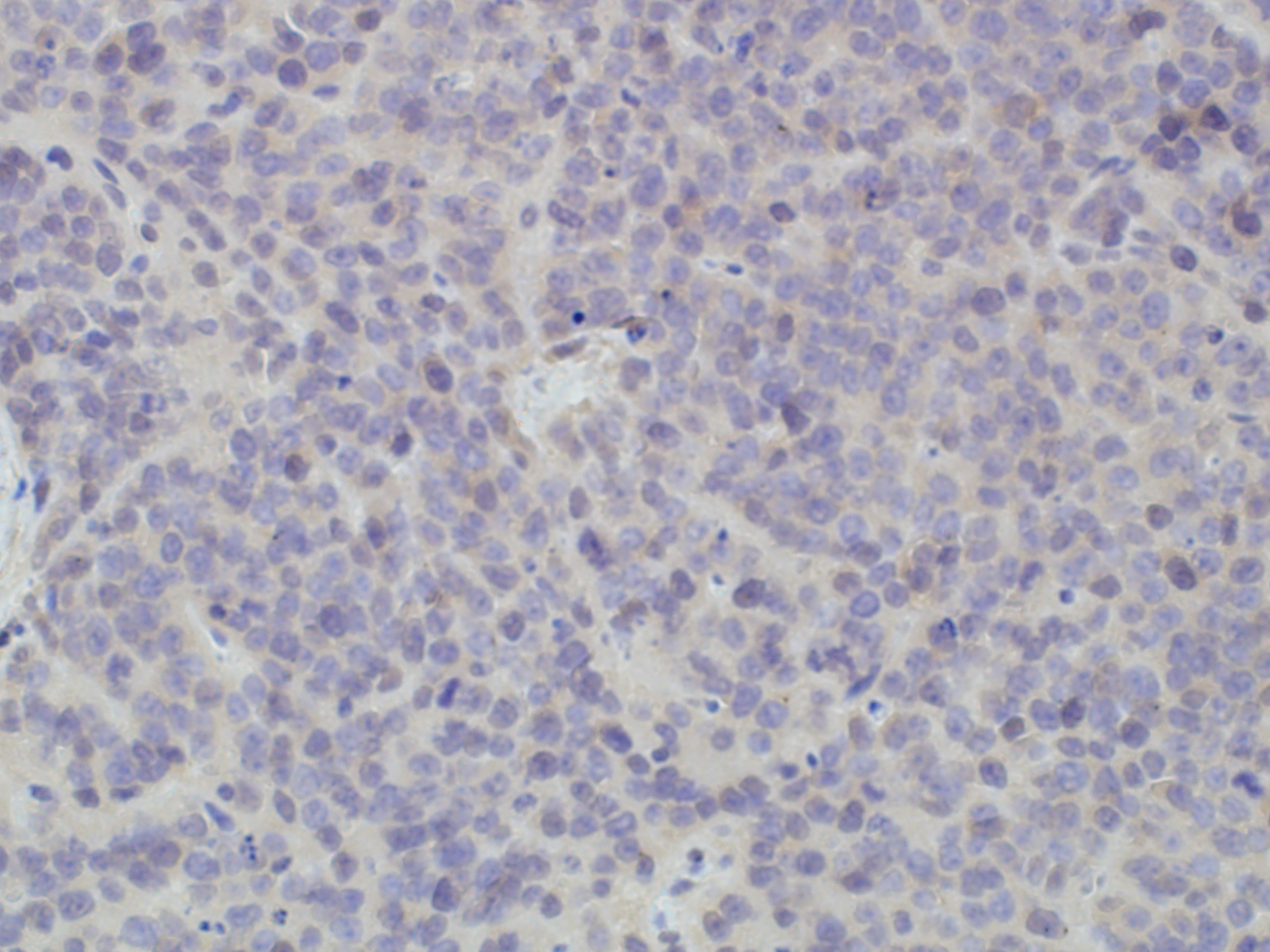

Supplement: Supplemental Information 9 [file peerj-10-13233-s009.zip › figure8/figure8 E/CDK6/miR-34a-5p.tif]

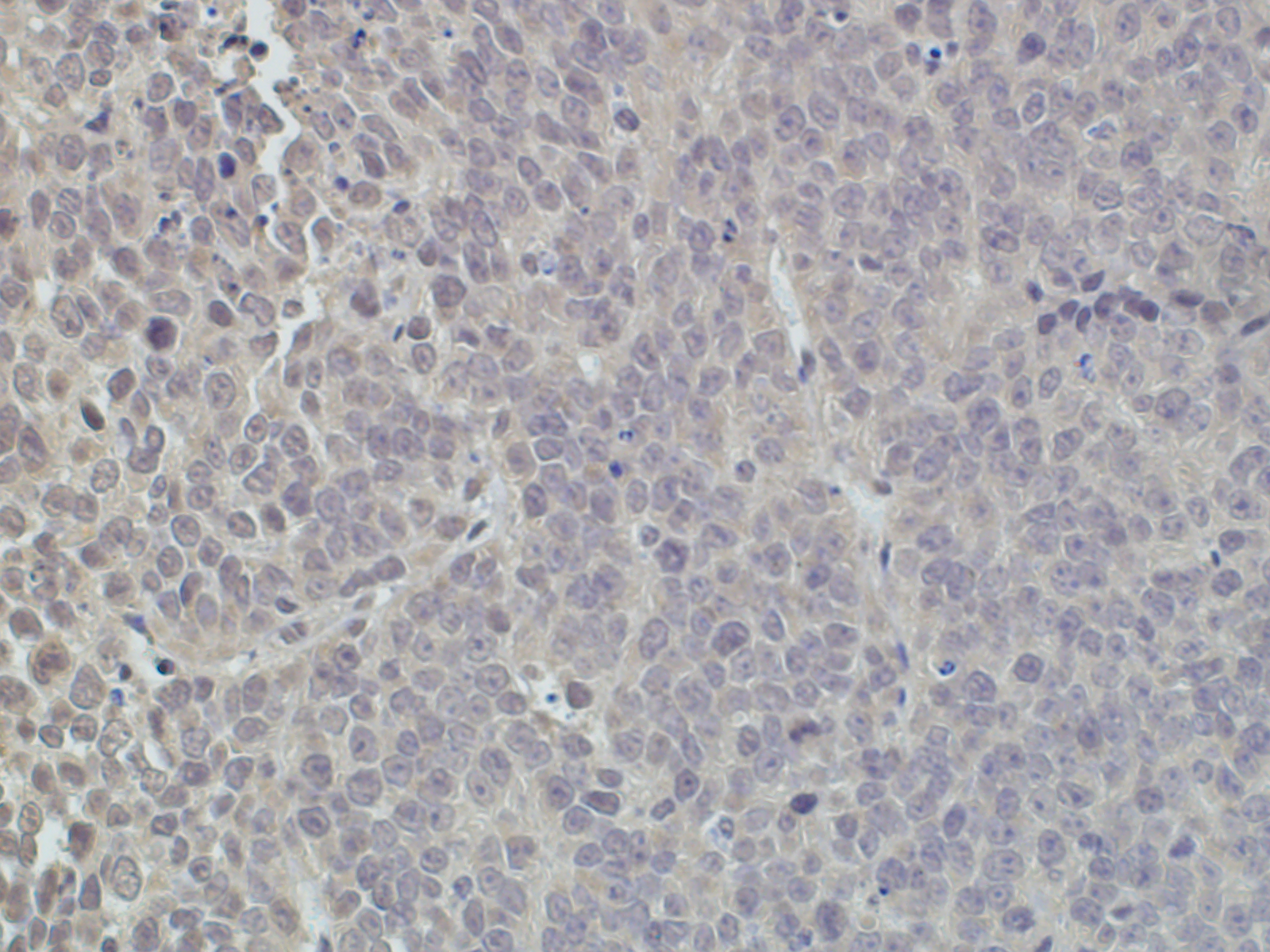

Supplement: Supplemental Information 9 [file peerj-10-13233-s009.zip › figure8/figure8 E/CDK6/miR-NC.tif]

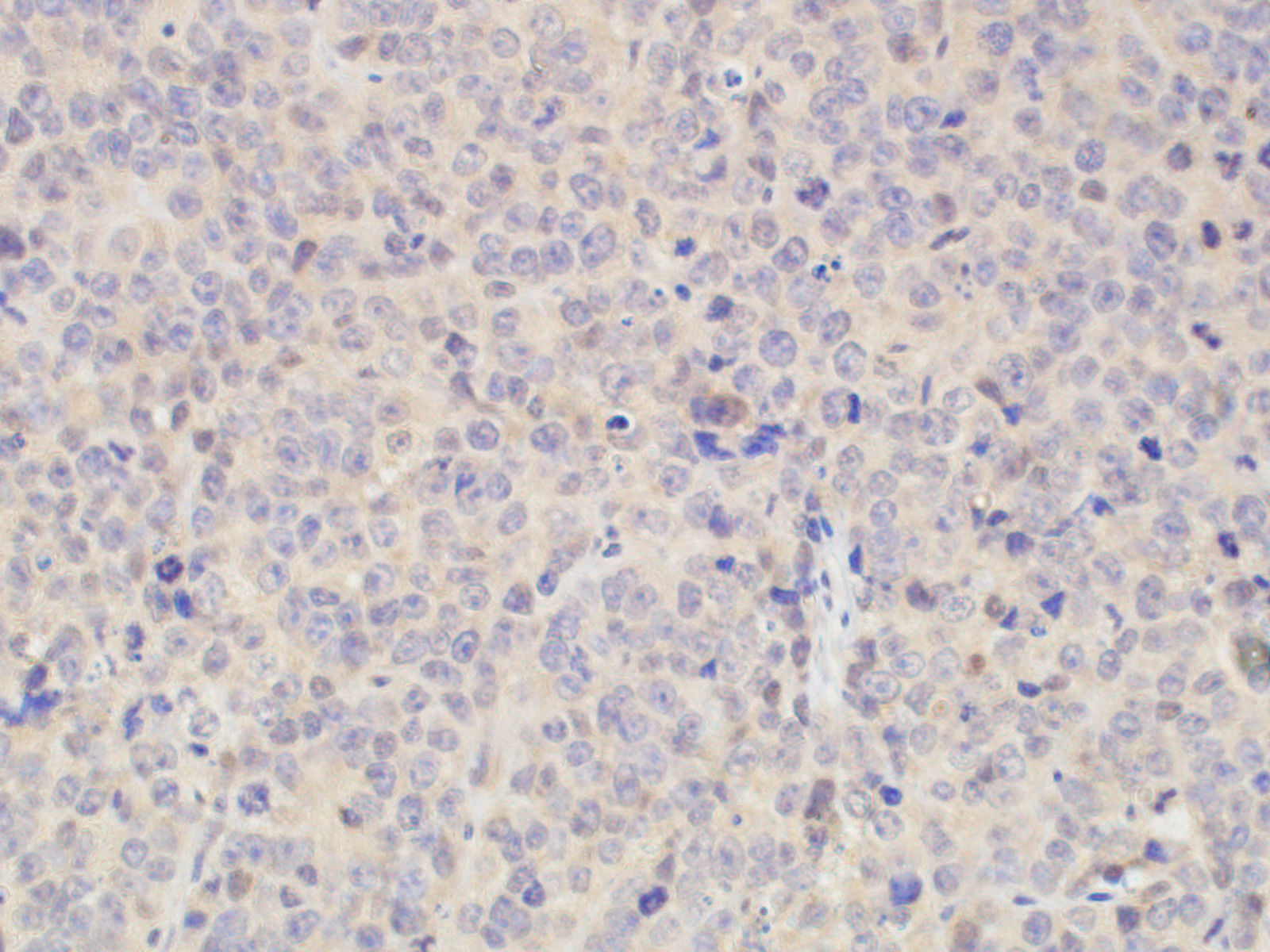

Supplement: Supplemental Information 9 [file peerj-10-13233-s009.zip › figure8/figure8 E/cyclin D1/miR-34a-5p.tif]

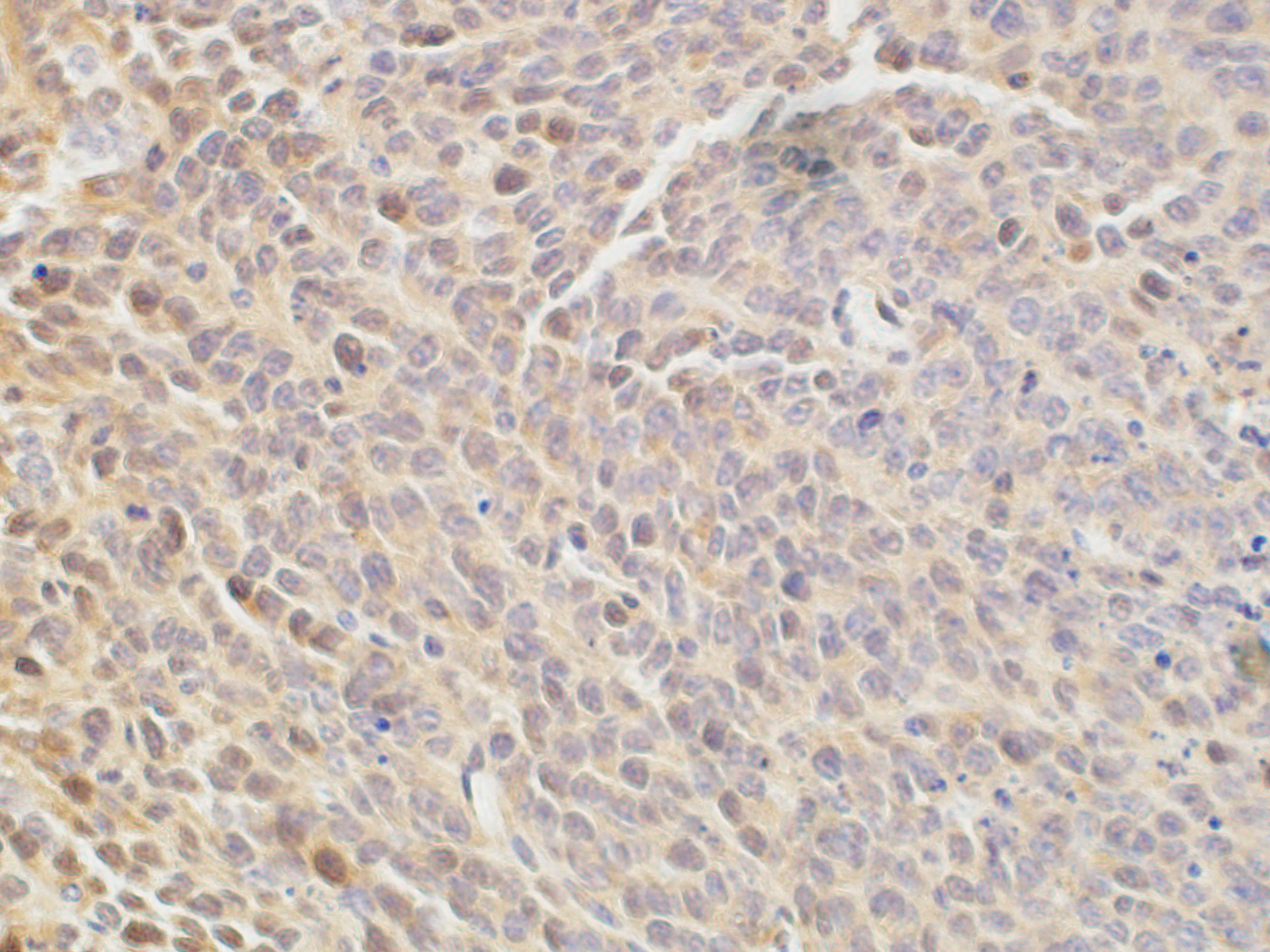

Supplement: Supplemental Information 9 [file peerj-10-13233-s009.zip › figure8/figure8 E/cyclin D1/miR-NC.tif]

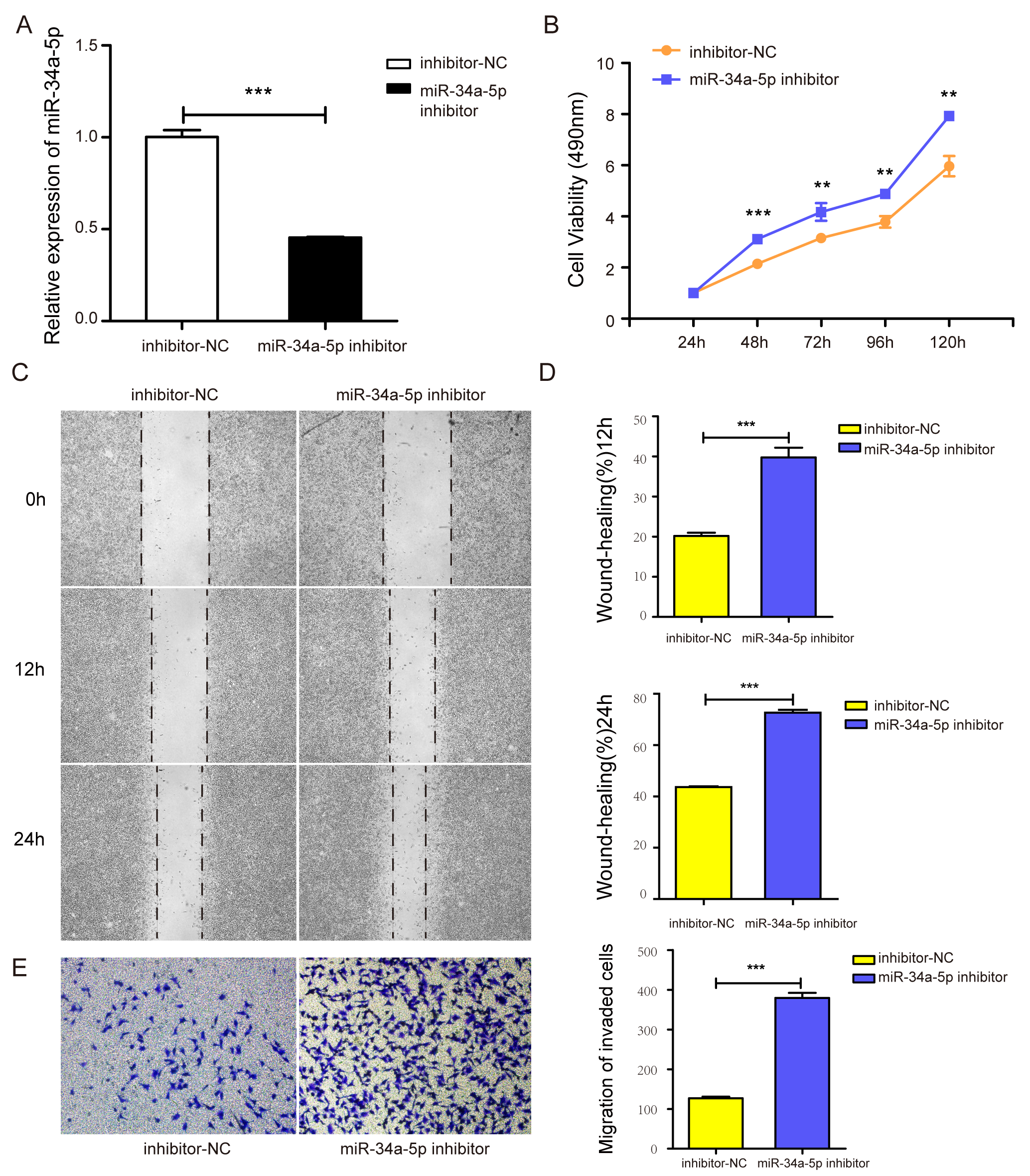

Supplement: Supplemental Information 10 — (A) The level of miR-34a-5p in SH-SY5Y cells transfected with miR-34a-5p inhibitor or inhibitor-NC was analyzed by real-time PCR. (B) The effect of the miR-34a-5p inhibitor on the proliferation of SH-SY5Y cells was detected by the MTT assay. (C–E) The cell migration was detected by the wound healing assay (C and D) and the transwell assay (E). Means ± SD for three independent experiments. *P < 0.05; **P < 0.01; ***P < 0.001. [file peerj-10-13233-s010.png]
